# Global burden and trends of subarachnoid hemorrhage in women aged 55 years and older: A repeated cross-sectional analysis of the GBD 2021 study

**DOI:** 10.1097/MD.0000000000051457

**Published:** 2026-11-06

**Authors:** Mei Zhu, Zhihua Cheng

**Affiliations:** a Department of Endocrinology, The Eighth People’s Hospital of Hefei, Hefei, Anhui, China; b Department of Neurosurgery, The Fourth Affiliated Hospital of Anhui Medical University, Hefei, Anhui, China.

**Keywords:** Global Burden of Disease study, health inequalities, postmenopausal women, sociodemographic index, subarachnoid hemorrhage

## Abstract

Subarachnoid hemorrhage (SAH) is a highly fatal stroke subtype with high mortality and disability. Women aged 55 years and older, approximating the postmenopausal population, carry a substantial share of this burden, yet global estimates for this group remain poorly defined. In this repeated cross-sectional analysis of Global Burden of Disease 2021 data, we examined incidence, prevalence, mortality, and disability-adjusted life years (DALYs) among women aged 55 years and older in 204 countries and territories from 1990 to 2021. Rates were directly age-standardized to the World Health Organization World Standard Population, and trends were summarized using the estimated annual percentage change from log-linear regression. We decomposed demographic and epidemiological drivers. Socioeconomic inequality was assessed with the concentration index and the slope index of inequality against the sociodemographic index (SDI). Between 1990 and 2021, the absolute number of incident and prevalent cases increased sharply, whereas deaths and DALYs declined slightly, even as age-standardized rates fell (all 4 rate trends declined significantly; estimated annual percentage change for the incidence rate = −1.65, 95% confidence interval = −1.77 to −1.52). The burden increased sharply after age 70. East Asia, particularly China, saw the sharpest declines in incidence and mortality rates, whereas India, Indonesia, and sub-Saharan Africa saw notable increases in absolute case numbers. Demographic shifts, mainly population aging and growth, drove the rising burden. Improvement in high-SDI regions explained little. After age standardization, none of the 4 rates was clearly correlated with SDI. Inequality analyses showed absolute gaps that narrowed for deaths and DALYs but widened slightly for prevalence, with prevalent cases concentrated in higher-SDI countries and deaths and DALYs in lower-SDI countries. The absolute burden of SAH in women aged 55 years and older is increasing because of population aging and socioeconomic disparities. Priorities should include targeted blood pressure screening and control for this age group in primary care, standardized emergency neurosurgical referral pathways, and expanded access to noninvasive neurovascular imaging in low and low-middle SDI regions.

## 1. Introduction

Subarachnoid hemorrhage (SAH), a devastating cerebrovascular disorder that accounts for only a small percentage of strokes, has disproportionately high morbidity, mortality, and long-term disability.^[1]^ Patients often have sudden catastrophic neurological deficits and social and economic consequences.^[2,3]^ Improvements in neurosurgical clipping, endovascular coiling, and neurocritical care have improved survival, but SAH mortality remains among the highest stroke subtypes, and survivors may sustain cognitive and functional impairment for years.^[4]^ Postmenopausal women have been proposed as a particularly vulnerable group. Mechanistic and experimental work indicates that the abrupt decline in circulating estrogen after menopause may promote endothelial dysfunction, inflammation, and extracellular matrix degeneration, thereby favoring aneurysm formation and rupture.^[5–7]^ These mechanisms are established in vascular biology; however, they cannot be assumed to account for the epidemiological distribution described here. Epidemiological data show that women have a higher incidence and worse outcomes than men, and the disparities are more evident with older age.^[8]^ Most global and regional studies focus on general populations and rarely consider postmenopausal women as a high-risk group. This leaves important gaps in understanding how aging and hormonal status affect disease burden.

In the last 20 years, clinical and epidemiological studies have shown that reproductive history and estrogen deficiency are associated with SAH risk.^[9–11]^ Observational data show that earlier menopause and shorter reproductive years increase aneurysmal rupture.^[9,11]^ Circulating estradiol, which falls sharply after menopause, increases the risk of vascular rupture.^[12]^ Genetic data show that estrogen receptor variants can influence the risk for postmenopausal women.^[13]^ These biological data are still not incorporated into population epidemiology.^[9]^ Socioeconomic factors affect both risk and access to care.^[1,14]^ The sociodemographic index (SDI) combining income, education, and fertility has been useful for defining global disparities of noncommunicable disease, but its relationship to SAH burden in postmenopausal women has not been evaluated.^[1,15]^ Lack of integrated approaches to biological vulnerability and socioeconomic inequality hinders targeted prevention and intervention.^[16]^ These clinical, epidemiological, and genetic findings are, however, still derived at the individual level, and the extent to which they shape the population-level burden described here cannot be determined by the present design.

A global SAH burden assessment of postmenopausal women is needed.^[15]^ Global Burden of Disease (GBD) 2021 provides standardized epidemiological estimates of 371 diseases in 204 countries and territories from 1990 to 2021, providing the ideal source for such estimations.^[17]^ Using GBD 2021, we assessed the incidence, prevalence, mortality, and disability-adjusted life years (DALYs) of postmenopausal women 55+ years old as a proxy for postmenopausal women.^[18]^ We measured temporal trends using incidence, prevalence, mortality, and DALY rates using estimated annual percentage changes (EAPCs). Age-specific and regional patterns were examined, the association between SDI and SAH burden was assessed, and decomposition and inequality analysis of demographic and epidemiological drivers were performed.^[18]^ Because menopausal status is not recorded in GBD, we use age 55 years and older throughout as an operational, age-based proxy for the postmenopausal population; this definition is not a biological classification. Combining this population-level assessment with clinical and vascular-age information provides the first global estimate of SAH burden in this group and informs public health policy, resource allocation, and age- and gender-specific stroke prevention and management strategy.

## 2. Materials and methods

### 2.1. Data sources

We used GBD 2021 data to estimate the SAH burden of postmenopausal women (aged 55 years and older) between 1990 and 2021 as measured by incidence, prevalence, deaths, and DALYs. The GBD 2021 provides standardized epidemiological estimates for 371 diseases and injuries in 204 countries and territories.^[19]^ All data are available on the Global Health Data Exchange. Since the GBD does not record menopausal status directly, we used age 55 years and older as an operational, age-based proxy for postmenopausal status, following the approach of previous GBD epidemiological studies; this proxy does not capture individual variation in the timing of menopause, nor premature or early menopause.^[20,21]^

### 2.2. Case definition

GBD defined SAH by International Classification of Diseases-9 (430) and 10 (I60) codes, based on population surveys, hospital data, mortality surveillance, and model estimates.^[17]^ GBD uses integrated hierarchical modeling to harmonize mortality and morbidity estimates, and a “fatal–nonfatal distribution” adjustment to compare regions.

### 2.3. SDI and stratification

Regional differences and socioeconomic correlations of SAH burden were measured using SDI and 21 GBD regions. SDI is a composite index of per capita income, mean years of education, and total fertility rate, divided into 5 quintiles (high, high-middle, middle, low-middle, and low).^[17,19]^ For decomposition and inequality, we used SDI to rank countries and stratify disease burdens at different social development levels.

### 2.4. Statistical analysis

All analyses and visualizations were done in R version 4.4.2 (R Foundation for Statistical Computing, Vienna, Austria). The main R packages used were dplyr, tidyr, ggplot2, and data.table. Rates for women aged 55 years and older were obtained by direct standardization to the World Health Organization (WHO) World Standard Population (WHO 2000–2025), renormalized within the 55 years and older age range and applied to the 5-year age-specific GBD estimates for that range; the quantity reported throughout is therefore a rate standardized within the 55 years and older population, and not an all-age standardized rate. Trends were summarized as the EAPC. EAPC was obtained using a log-linear regression model fitted to all annual values from 1990 to 2021: ln(y) = α + βx + ε, where y is the age-standardized rate and x is the calendar year. EAPC was calculated as EAPC = 100 × (e^β^ − 1), and its 95% confidence interval (95% CI) was obtained from the standard error of β. An EAPC >0 is an increasing trend; an EAPC <0 is a decreasing trend. If the 95% CI includes 0, the trend is not statistically significant. Because GBD estimates are modeled outputs rather than independent observations, these intervals describe the fit of the log-linear trend and should not be interpreted as sampling-based confidence intervals.

To identify drivers of burden change, we used the Andreev decomposition method and partitioned differences in cases and DALYs between 1990 and 2021 into 3 components: age effect, population growth effect, and epidemiological change.^[22]^

The associations between SAH burden and SDI were correlated using Spearman’s rank correlation, and we summarize results as the correlation coefficient (ρ) and *P* value.^[23]^

To examine whether the decline in SAH burden tracked changes in cardiovascular risk-factor exposure, we additionally analyzed the summary exposure value (SEV) for smoking and for high systolic blood pressure among women aged 55 years and older, from the same GBD 2021 release, for the global level, the 21 GBD regions, and 204 countries and territories (1990–2021). The SEV is a risk-standardized measure of population exposure that compares the observed distribution of exposure with a theoretical minimum-risk distribution, so that a higher value indicates greater population exposure. SEV estimates were correlated with the age-standardized SAH rates using the Spearman rank correlation described above, both pooled across location-years and, for the change analysis, across the 21 GBD regions using the EAPC of each quantity; correlations additionally controlling for the SDI and for calendar year were obtained by rank partial correlation (reported in the “Results” section).

Socioeconomic inequality was evaluated using the concentration index (CI) and the slope index of inequality (SII).^[24]^ The CI was computed as the population-weighted Wagstaff CI of the age-standardized rate, with countries ranked by SDI and weighted by the female population aged 55 years and older; a negative value indicates that disease burden is concentrated in low-SDI countries and a positive value indicates that it is concentrated in high-SDI countries. The SII was estimated by population-weighted least squares regression of the age-standardized rate on the same population-weighted mid-rank of SDI, using the same female population aged 55 years and older weights as the CI, and represents the absolute difference in burden between the highest and the lowest SDI. Because SDI is not available for Taiwan, China in the GBD 2021 SDI file, the inequality analyses were based on 203 countries, whereas the rate tables include all 204 countries and territories.

Rates are given per 100,000 population, and estimates are 95% uncertainty intervals (UIs).

### 2.5. Ethics statement

This study was based exclusively on publicly available, de-identified aggregate estimates from the GBD 2021 study. No individual-level participant data were accessed, and no human participants or animals were involved. In accordance with the institutional policy of The Fourth Affiliated Hospital of Anhui Medical University, secondary analyses of openly available, nonidentifiable aggregate data of this kind are exempt from ethics committee review and from the requirement for informed consent.

## 3. Results

### 3.1. Global burden of SAH in postmenopausal women

Between 1990 and 2021, the number of SAH incident cases worldwide increased from 125,234 (95% UI: 102,816–152,368) to 189,786 (95% UI: 159,916–225,999), with an EAPC of 1.01% (95% CI: 0.84–1.18). The number of prevalent cases increased from 1,278,117 (95% UI: 1,133,251–1,448,200) to 2,406,800 (95% UI: 2,172,485–2,684,226), with an EAPC of 2.05% (95% CI: 1.97–2.13). DALYs decreased from 3,258,391 (95% UI: 2,271,519–4,013,050) to 3,154,388 (95% UI: 2,808,234–3,577,128), with an EAPC of −0.59% (95% CI: −0.90 to −0.27). Deaths decreased from 146,479 (95% UI: 95,664–184,176) to 143,240 (95% UI: 123,111–166,293), with an EAPC of −0.61% (95% CI: −0.92 to −0.29; Table 1). Although the absolute number of cases increased, the rates of all key indicators declined significantly. The age-standardized incidence rate dropped from 34.39 per 100,000 (95% UI: 24.10–47.71) to 22.99 (95% UI: 16.61–31.02) with an EAPC of −1.65% (95% CI: −1.77 to −1.52). The age-standardized prevalence rate decreased from 353.63 (95% UI: 299.81–413.99) to 301.73 (95% UI: 263.73–345.08) with an EAPC of −0.56% (95% CI: −0.58 to −0.53). The overall disease burden was also greatly reduced. The age-standardized DALY rate fell from 898.63 (95% UI: 627.81–1116.06) to 390.85 (95% UI: 342.53–450.60) with an EAPC of −3.15% (95% CI: −3.40 to −2.89); the mortality rate fell from 39.86 (95% UI: 26.35–50.32) to 16.78 (95% UI: 14.28–19.85) with an EAPC of −3.31% (95% CI: −3.58 to −3.04; Table 1 and Table S1, Supplemental Digital Content 1).

**Table 1:** Case counts (95% UI) and EAPC (95% CI) for SAH outcomes in postmenopausal women across GBD regions, 1990–2021.

Location	Incidence (95% UI)			Prevalence (95% UI)			DALYs (95% UI)			Deaths (95% UI)		
	1990	2021	EAPC	1990	2021	EAPC	1990	2021	EAPC	1990	2021	EAPC
Global	125,234 (102,816, 152,368)	189,786 (159,916, 225,999)	1.01 (0.84, 1.18)	1,278,117 (1,133,251, 1,448,200)	2,406,800 (2,172,485, 2,684,226)	2.05 (1.97, 2.13)	3,258,391 (2,271,519, 4,013,050)	3,154,388 (2,808,234, 3,577,128)	−0.59 (−0.90, −0.27)	146,479 (95,664, 184,176)	143,240 (123,111, 166,293)	−0.61 (−0.92, −0.29)
Andean Latin America	753 (627, 895)	1808 (1534, 2141)	2.98 (2.88, 3.08)	12,285 (11,439, 13,230)	30,397 (28,478, 32,236)	2.96 (2.93, 2.99)	13,889 (10,045, 18,084)	34,592 (28,522, 42,186)	2.91 (2.72, 3.10)	552 (387, 752)	1475 (1173, 1823)	3.21 (3.02, 3.40)
Australasia	594 (513, 692)	993 (843, 1175)	1.70 (1.64, 1.76)	7645 (7041, 8277)	15,062 (13,990, 16,080)	2.31 (2.26, 2.35)	11,008 (10,112, 11,989)	12,881 (11,324, 14,282)	0.50 (0.39, 0.61)	498 (453, 542)	646 (537, 719)	0.81 (0.69, 0.93)
Caribbean	683 (564, 825)	1335 (1120, 1594)	2.07 (2.02, 2.12)	11,832 (11,055, 12,643)	23,165 (21,720, 24,531)	2.13 (2.09, 2.16)	13,410 (10,162, 16,207)	22,805 (17,913, 28,804)	1.69 (1.63, 1.76)	536 (392, 657)	927 (724, 1180)	1.73 (1.67, 1.80)
Central Asia	1161 (988, 1370)	1805 (1581, 2079)	1.51 (1.26, 1.76)	10,623 (9671, 11,635)	16,727 (15,479, 18,028)	1.21 (0.86, 1.57)	18,345 (16,257, 21,761)	33,821 (30,441, 37,615)	1.85 (1.60, 2.10)	787 (689, 967)	1520 (1359, 1689)	2.23 (1.96, 2.50)
Central Europe	3604 (3119, 4190)	4748 (4313, 5257)	0.84 (0.75, 0.94)	34,016 (30,471, 37,642)	35,215 (31,754, 38,918)	0.15 (0.07, 0.24)	74,198 (68,646, 80,794)	67,978 (62,232, 73,799)	−0.33 (−0.59, −0.08)	3034 (2800, 3293)	3363 (3034, 3671)	0.33 (0.05, 0.60)
Central Latin America	2333 (1919, 2860)	7874 (6662, 9355)	4.07 (4.02, 4.12)	42,177 (38,817, 46,214)	126,067 (116,400, 136,072)	3.64 (3.61, 3.67)	32,920 (31,039, 34,979)	116,101 (103,601, 130,499)	4.28 (4.09, 4.46)	1271 (1204, 1339)	4943 (4336, 5563)	4.70 (4.46, 4.94)
Central Sub-Saharan Africa	378 (282, 497)	868 (679, 1109)	2.53 (2.44, 2.61)	6070 (5542, 6683)	14,230 (13,161, 15,348)	2.61 (2.51, 2.72)	6425 (3644, 13,771)	13,942 (8345, 31,108)	2.35 (2.28, 2.41)	222 (112, 522)	499 (266, 1234)	2.45 (2.38, 2.53)
East Asia	40,994 (33,233, 50,759)	45,364 (38,134, 54,193)	−0.46 (−0.88, −0.05)	250,591 (211,348, 296,945)	427,708 (371,803, 497,882)	1.65 (1.43, 1.88)	1,727,079 (770,956, 2,208,648)	815,829 (597,001, 1,069,370)	−3.68 (−4.53, −2.82)	82,374 (33,920, 106,017)	39,862 (27,902, 53,719)	−3.73 (−4.64, −2.82)
Eastern Europe	7649 (6243, 9384)	9663 (8196, 11,338)	0.53 (0.29, 0.77)	69,014 (58,867, 81,926)	81,127 (69,986, 94,255)	0.45 (0.34, 0.57)	139,203 (128,077, 151,180)	165,387 (148,665, 182,428)	−0.18 (−1.02, 0.66)	6442 (5891, 7036)	8660 (7668, 9609)	0.27 (−0.76, 1.31)
Eastern Sub-Saharan Africa	1283 (967, 1654)	2385 (1855, 3056)	1.85 (1.71, 1.99)	20,297 (18,229, 22,706)	38,809 (35,515, 42,258)	2.08 (1.94, 2.21)	19,638 (11,365, 47,761)	32,863 (19,418, 81,422)	1.59 (1.40, 1.77)	692 (362, 1880)	1165 (603, 3221)	1.62 (1.44, 1.80)
High-income Asia Pacific	13,913 (11,148, 17,227)	21,768 (18,401, 25,591)	1.34 (1.19, 1.48)	171,778 (149,717, 199,965)	397,463 (353,852, 450,918)	2.84 (2.71, 2.97)	205,666 (186,331, 227,011)	217,283 (181,976, 247,367)	−0.06 (−0.18, 0.06)	8582 (7555, 9547)	10,016 (7568, 11,527)	0.21 (0.07, 0.36)
High-income North America	7693 (6210, 9462)	12,922 (10,820, 15,498)	1.70 (1.45, 1.95)	115,708 (101,526, 135,484)	221,806 (198,659, 246,858)	2.15 (2.05, 2.26)	137,013 (128,217, 144,752)	209,843 (191,082, 224,778)	1.45 (1.34, 1.56)	5868 (5338, 6181)	9875 (8505, 10,625)	1.77 (1.60, 1.95)
North Africa and Middle East	2910 (2361, 3573)	5440 (4570, 6565)	1.77 (1.66, 1.89)	37,587 (34,124, 41,096)	79,110 (73,132, 84,816)	2.30 (2.20, 2.40)	65,391 (39,314, 124,381)	96,747 (75,521, 121,297)	1.12 (0.94, 1.30)	2738 (1565, 5523)	4186 (3197, 5510)	1.39 (1.21, 1.57)
Oceania	103 (86, 126)	220 (184, 258)	2.27 (2.18, 2.36)	1050 (965, 1144)	2373 (2200, 2546)	2.59 (2.46, 2.73)	2756 (1820, 4005)	5003 (3468, 7320)	1.81 (1.66, 1.97)	110 (71, 164)	203 (134, 299)	1.83 (1.72, 1.93)
South Asia	12,790 (10,075, 16,231)	25,430 (20,645, 31,079)	1.95 (1.81, 2.09)	107,145 (90,997, 128,451)	246,262 (213,518, 288,047)	2.72 (2.62, 2.82)	257,937 (137,048, 475,890)	533,499 (409,773, 724,086)	2.32 (2.18, 2.45)	9756 (5060, 19,096)	20,920 (15,787, 29,877)	2.48 (2.36, 2.59)
Southeast Asia	8574 (7054, 10,468)	17,985 (15,119, 21,890)	2.16 (2.05, 2.27)	95,958 (85,805, 107,347)	220,672 (200,259, 245,766)	2.58 (2.45, 2.71)	191,525 (132,524, 283,415)	341,750 (274,357, 485,836)	1.68 (1.61, 1.75)	8043 (5379, 12,770)	14,599 (11,297, 22,708)	1.75 (1.66, 1.83)
Southern Latin America	2026 (1684, 2482)	2607 (2219, 3072)	0.71 (0.58, 0.84)	21,439 (18,741, 24,557)	32,682 (27,861, 40,644)	1.51 (1.45, 1.58)	39,490 (35,164, 44,030)	34,785 (31,856, 37,799)	−0.16 (−0.37, 0.06)	1650 (1446, 1856)	1538 (1383, 1677)	0.06 (−0.18, 0.30)
Southern Sub-Saharan Africa	393 (307, 502)	822 (655, 1037)	2.22 (2.05, 2.39)	6899 (6045, 7918)	13,700 (12,217, 15,511)	2.05 (1.96, 2.14)	4218 (3486, 5669)	11,470 (9781, 14,065)	3.56 (3.19, 3.93)	151 (119, 214)	429 (353, 538)	3.73 (3.26, 4.20)
Tropical Latin America	2543 (2017, 3256)	6886 (5683, 8427)	3.06 (2.98, 3.15)	39,412 (33,375, 46,543)	91,267 (78,172, 106,843)	2.55 (2.41, 2.69)	55,033 (52,025, 58,452)	139,199 (128,015, 147,692)	3.10 (2.86, 3.34)	2027 (1911, 2158)	5781 (5204, 6149)	3.57 (3.29, 3.85)
Western Europe	13,982 (11,866, 16,603)	17,124 (14,929, 19,656)	0.61 (0.51, 0.70)	198,567 (181,469, 215,339)	255,190 (237,234, 273,347)	0.83 (0.80, 0.86)	223,595 (207,073, 237,720)	212,904 (188,044, 231,179)	−0.01 (−0.15, 0.13)	10,385 (9436, 11,086)	11,338 (9415, 12,406)	0.55 (0.34, 0.76)
Western Sub-Saharan Africa	874 (675, 1138)	1739 (1342, 2294)	2.05 (1.87, 2.23)	18,026 (16,288, 20,139)	37,765 (34,388, 41,401)	2.33 (2.17, 2.50)	19,651 (12,583, 37,788)	35,706 (24,778, 72,413)	1.85 (1.74, 1.97)	759 (445, 1595)	1295 (828, 2930)	1.63 (1.53, 1.73)

95% CI = 95% confidence interval, EAPC = estimated annual percentage change, GBD = Global Burden of Disease, SAH = subarachnoid hemorrhage, UI = uncertainty interval.

### 3.2. Regional and national-level burdens of SAH in postmenopausal women

The EAPC of the age-standardized incidence, mortality, and DALY rates in East Asia was −3.92%, −7.17%, and −6.87%, respectively, and the absolute numbers of deaths and DALYs in that region fell by 51.6% and 52.8%, despite a global rise in the absolute number of cases. Central Asia was the only region in which the age-standardized incidence rate increased (EAPC: 0.10%, 95% CI: 0.04–0.16). In Central Latin America, the mortality and DALY rates both increased (EAPC: 0.52% and 0.24%, respectively), and the absolute numbers of deaths and DALYs in that subregion rose by 289% and 253%. The largest relative increases in absolute case numbers occurred in Latin America and sub-Saharan Africa: incident cases rose by 237% in Central Latin America, 171% in Tropical Latin America, 140% in Andean Latin America, 130% in Central Sub-Saharan Africa, 99% in Western Sub-Saharan Africa, and 86% in Eastern Sub-Saharan Africa (Table 1 and Table S1, Supplemental Digital Content 1).

In absolute terms, the burden of cases increased substantially in several countries. India had a major increase in new cases (95% UI: 15,286–23,247) in 2021, almost double the number in 1990 (10,098). Indonesia had a rise from 3028 cases in 1990 to 6270 cases in 2021. Although the United States is a high-income country, it still has a large absolute burden: in 2021, new cases reached 11,355 and prevalent cases totaled 195,589 because of the large population (Table S2, Supplemental Digital Content 2). China had the largest reduction in the age-standardized SAH incidence, mortality, and DALY rates. Its incidence rate EAPC was −4.02% (95% CI: −4.37 to −3.66), its mortality rate EAPC was −7.29% (95% CI: −8.10 to −6.46), and its DALY rate EAPC was −7.00% (95% CI: −7.77 to −6.23) (Fig. 1, Fig. S1, Supplemental Digital Content 3, and Table S3, Supplemental Digital Content 4).

**Figure 1.**
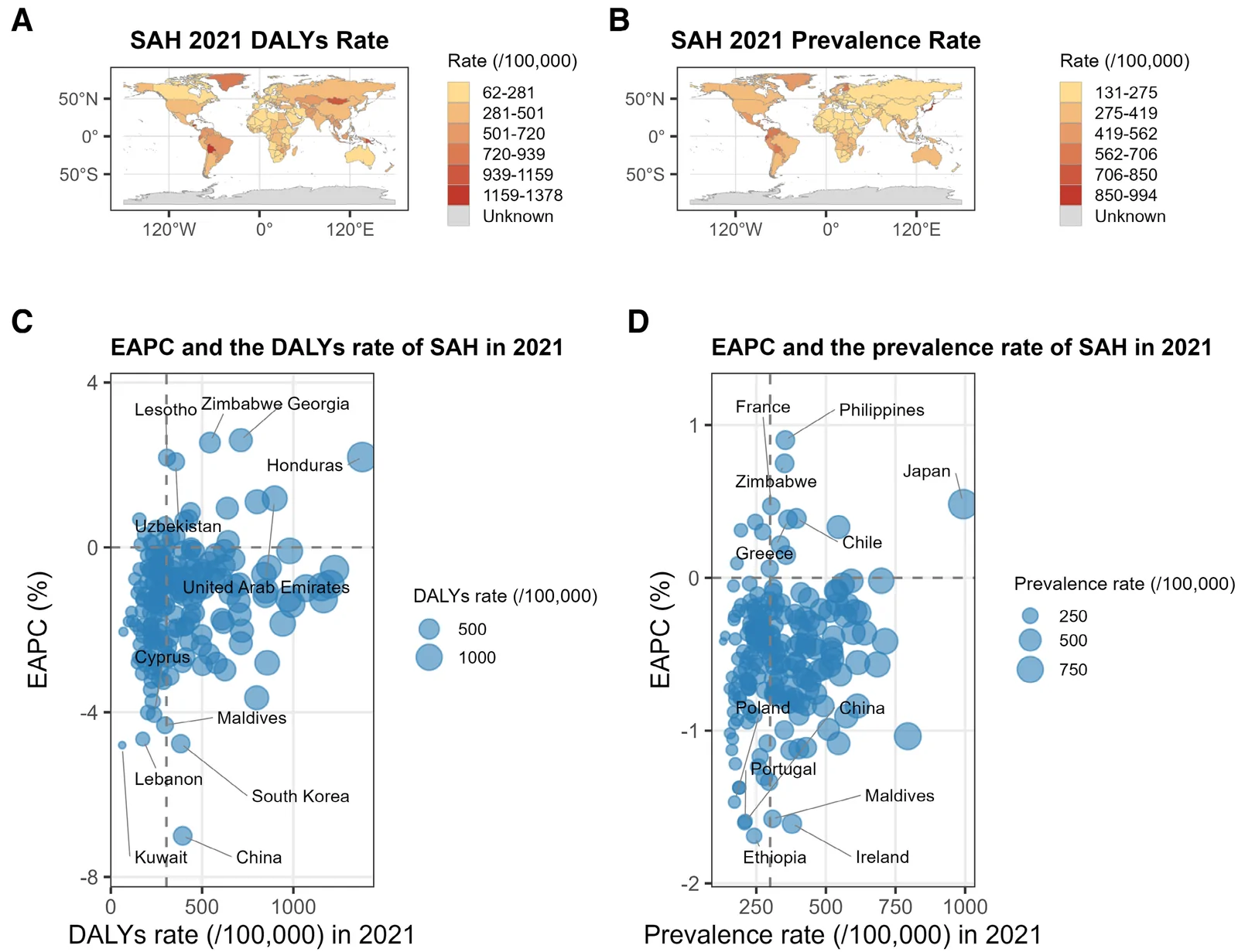
Global distribution and temporal trends of DALY and prevalence rates of SAH among postmenopausal women in 2021. (A) DALY rate. (B) Prevalence rate. (C) Association between EAPC and DALY rate in 2021. (D) Association between EAPC and prevalence rate in 2021. Rates are expressed per 100,000 women aged 55 years and older. DALYs = disability-adjusted life years, EAPC = estimated annual percentage change, SAH = subarachnoid hemorrhage.

### 3.3. Age-specific disease burden of SAH in postmenopausal women

In 2021, the absolute number of SAH cases among postmenopausal women peaked in the 55 to 59 age group and declined thereafter. Deaths rose sharply after the 65 age group, and mortality among postmenopausal women 80-plus is much higher than that among men (Fig. 2). In contrast, incidence, prevalence, DALY, and mortality rates all increased with age and reached their maximum in the 85+ age group (Fig. S2, Supplemental Digital Content 5).

**Figure 2.**
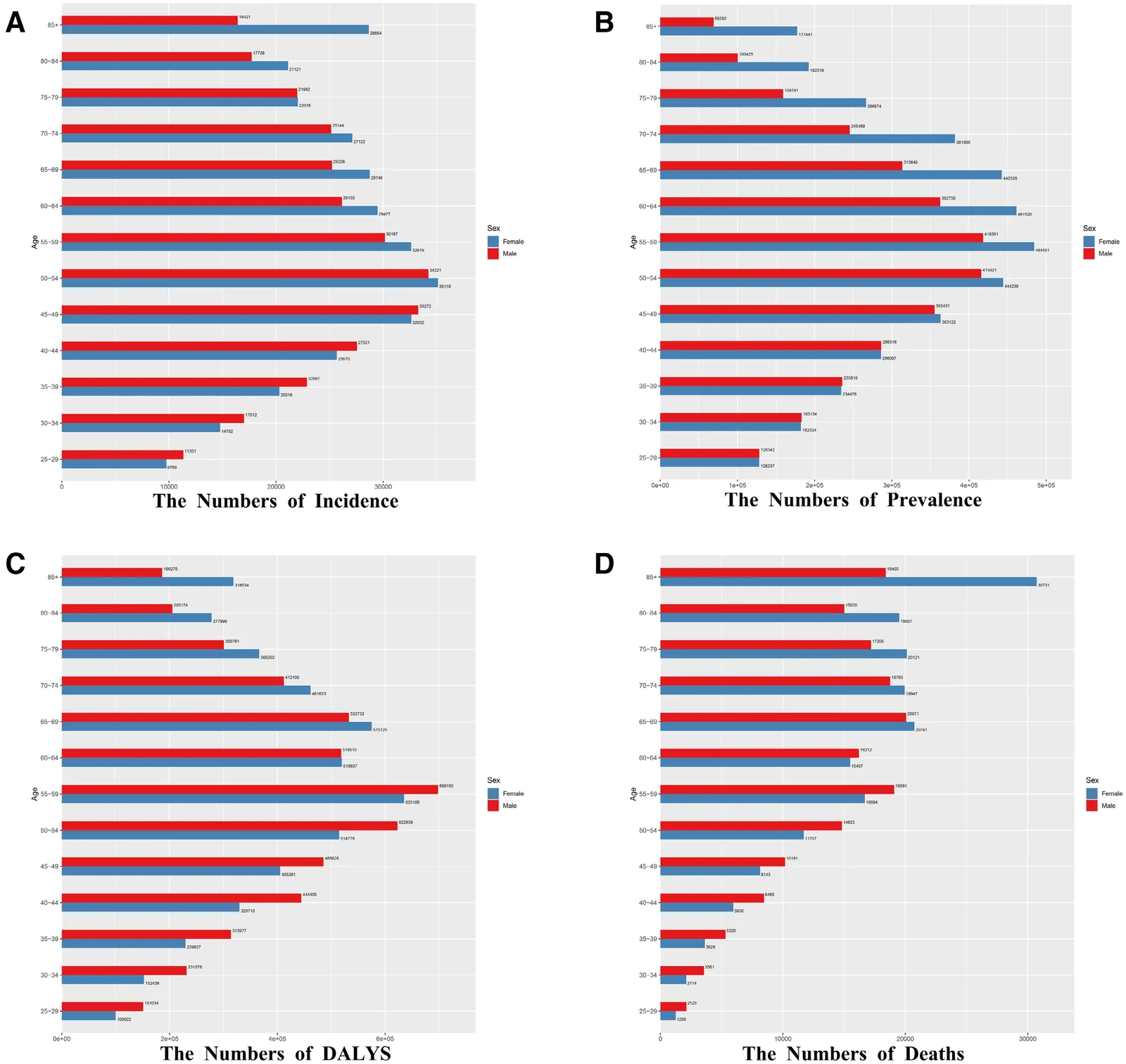
In 2021, the number of subarachnoid hemorrhage cases among postmenopausal women compared with age-matched men and younger women (25–54 years). (A) Number of incidence cases. (B) Number of prevalence cases. (C) Number of DALYs. (D) Number of deaths. DALYs = disability-adjusted life years.

### 3.4. The temporal trend of SAH in postmenopausal women from 1990 to 2021

Globally, the number of SAH cases for postmenopausal women increased steadily from 1990 to 2021 (Fig. 3 and Table 1) for all age groups (Fig. 3A and B). The DALY count for postmenopausal women declined around 2010 and then rebounded (Fig. 3C). Deaths decreased in all age groups until about 2005 and then slowly rose in the 85+ age group (deaths briefly decreased from 2005 to 2010 but again increased in the following years).

**Figure 3.**
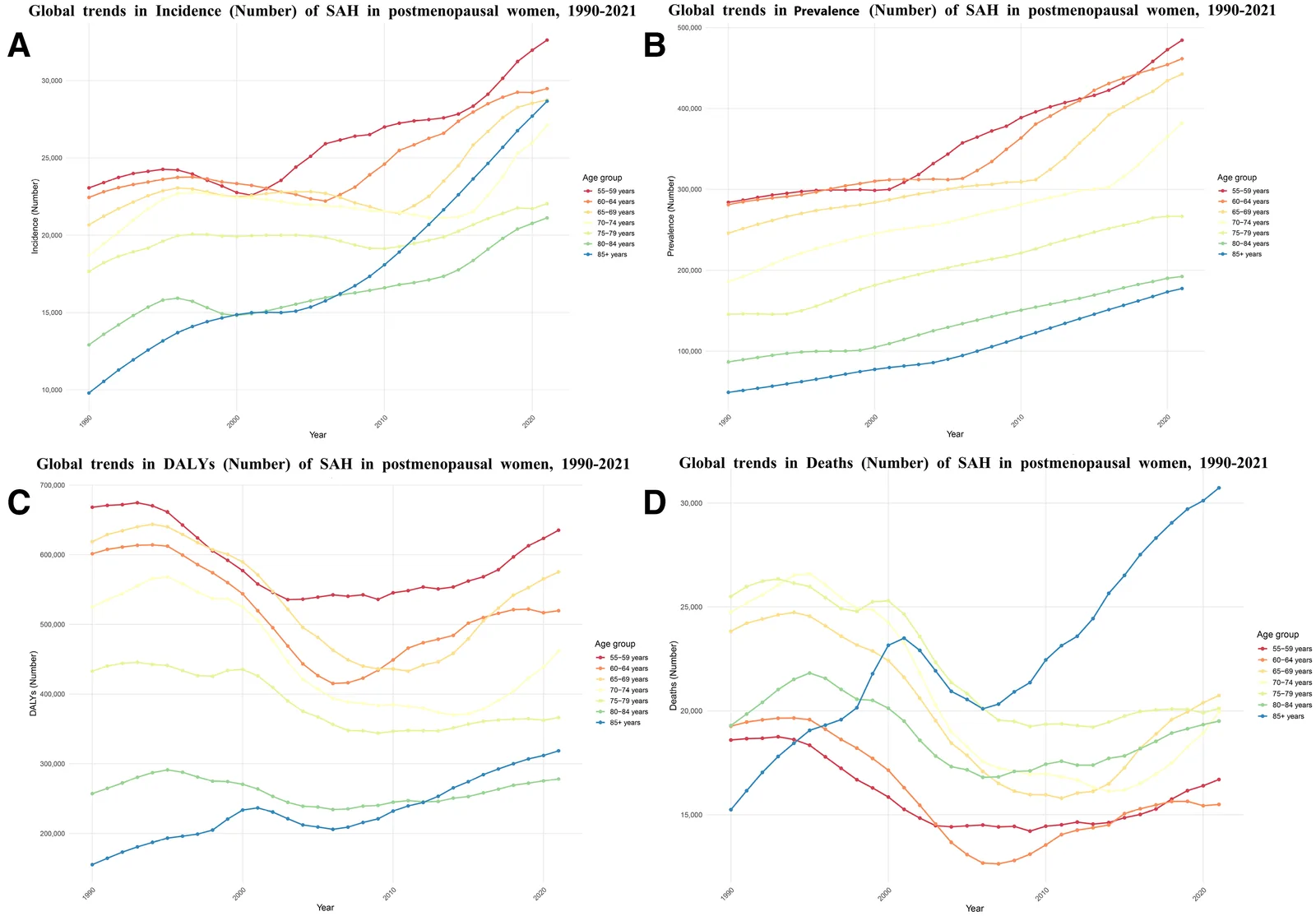
Global temporal trends in the absolute numbers of SAH cases among postmenopausal women from 1990 to 2021. (A) Trends in the number of incident cases by age group. (B) Trends in the number of prevalent cases by age group. (C) Trends in the number of DALYs by age group. (D) Trends in the number of deaths by age group. DALYs = disability-adjusted life years, SAH = subarachnoid hemorrhage.

In contrast, age-specific SAH rates of postmenopausal women have declined since 1990 overall (Fig. S3, Supplemental Digital Content 6 and Table S1, Supplemental Digital Content 1). Incidence and prevalence rates decreased for most age groups, most markedly for the 55 to 64 age group (Fig. S3A and B, Supplemental Digital Content 6). DALYs and mortality rates decreased for all age groups, and the DALY rate decreased most markedly for the 65 to 74 age group (Fig. S3C and D, Supplemental Digital Content 6 and Table S1, Supplemental Digital Content 1).

### 3.5. The correlation between the disease burden of SAH in postmenopausal women and the SDI

The correlations between the age-standardized SAH rates and the SDI were weak for all 4 outcomes, and none reached statistical significance (Fig. 4 and Table S4, Supplemental Digital Content 7). Across the 21 GBD regions and the global level over 32 years (n = 704), the correlation coefficients were ρ = 0.064 (*P* = .09) for incidence, ρ = 0.066 (*P* = .08) for prevalence, ρ = 0.010 (*P* = .79) for mortality, and ρ = −0.021 (*P* = .57) for DALYs. When the same analysis was applied to the unstandardized rates for women aged 55 years and older, the coefficients were larger (ρ = 0.161, 0.084, 0.159, and 0.031 for incidence, prevalence, mortality, and DALYs); the first 3 reached statistical significance (*P* < .001, *P* = .03, and *P* < .001 respectively), whereas the DALY correlation did not (*P* = .42); the loss of significance after standardization indicates that the crude association was driven largely by the differing age structure of the 55 years and older female population across regions rather than by the SDI itself.

**Figure 4.**
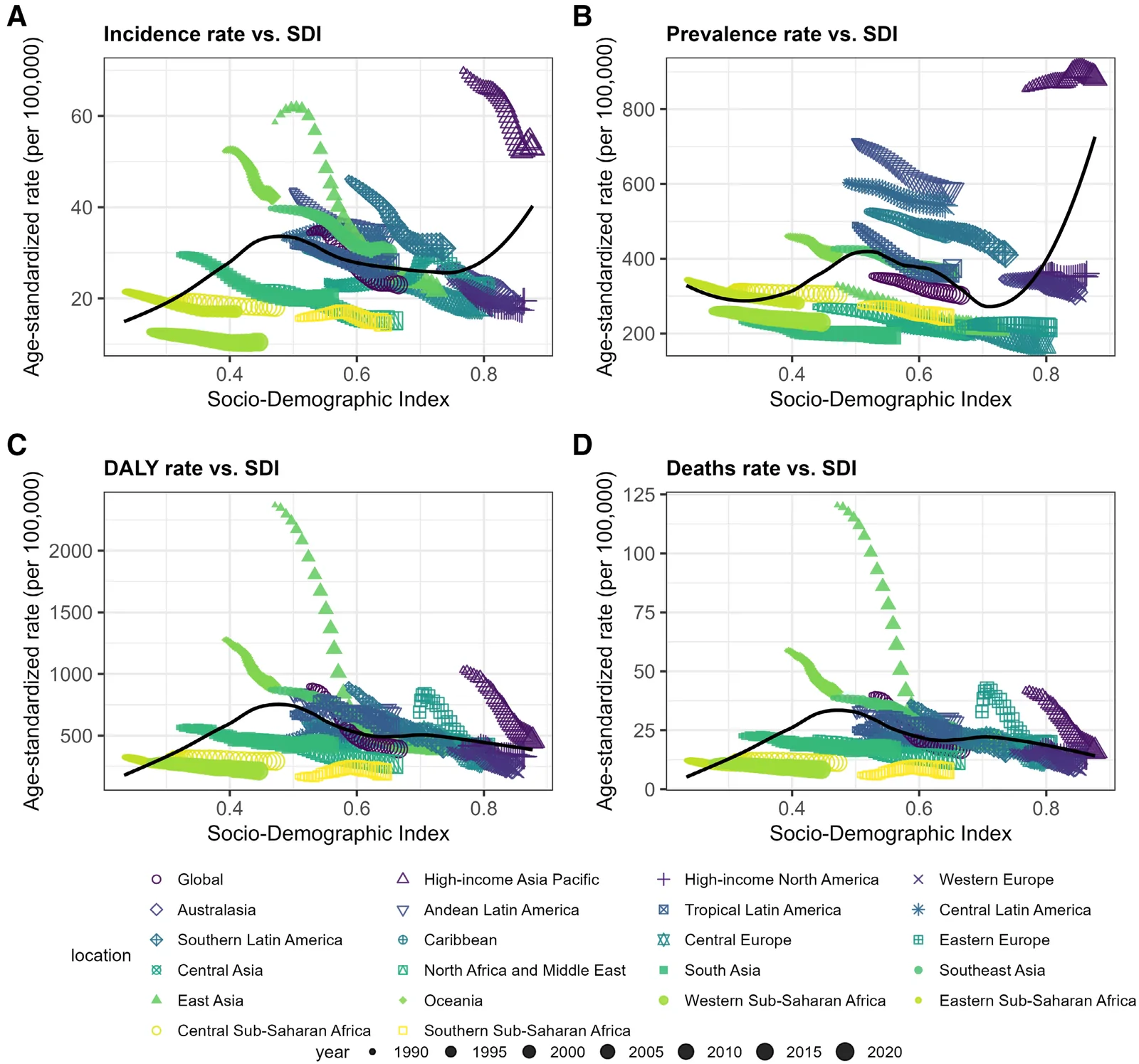
Correlation between rates of subarachnoid hemorrhage and SDI among women aged 55 years and older across 21 GBD regions and the global level, 1990–2021. (A) Incidence rate versus SDI. (B) Prevalence rate versus SDI. (C) DALY rate versus SDI. (D) Mortality rate versus SDI. Rates were age-standardized to the World Health Organization World Standard Population within the 55 years and older age range. DALYs = disability-adjusted life years, GBD = Global Burden of Disease, SDI = sociodemographic index.

### 3.6. Decomposition analysis of SAH disease burden in postmenopausal women based on SDI levels

Between 1990 and 2021, the absolute increase in SAH prevalence (+1,128,683 cases) was driven almost entirely by population growth (+1,408,318 cases), partly offset by a 25.5% decline in age-specific prevalence rates (−287,577 cases). For deaths and DALYs, population growth and aging together would have added 133,856 deaths and 2.83 million DALYs, but these were more than compensated by substantial epidemiological improvement: mortality risk reduction prevented 137,095 deaths, and DALY rate reduction prevented 2.93 million DALYs. As a result, total deaths and DALYs showed only slight net decreases (−3239 and −104,003, respectively) (Fig. 5, Fig. S4, Supplemental Digital Content 8, and Table S5, Supplemental Digital Content 9).

**Figure 5.**
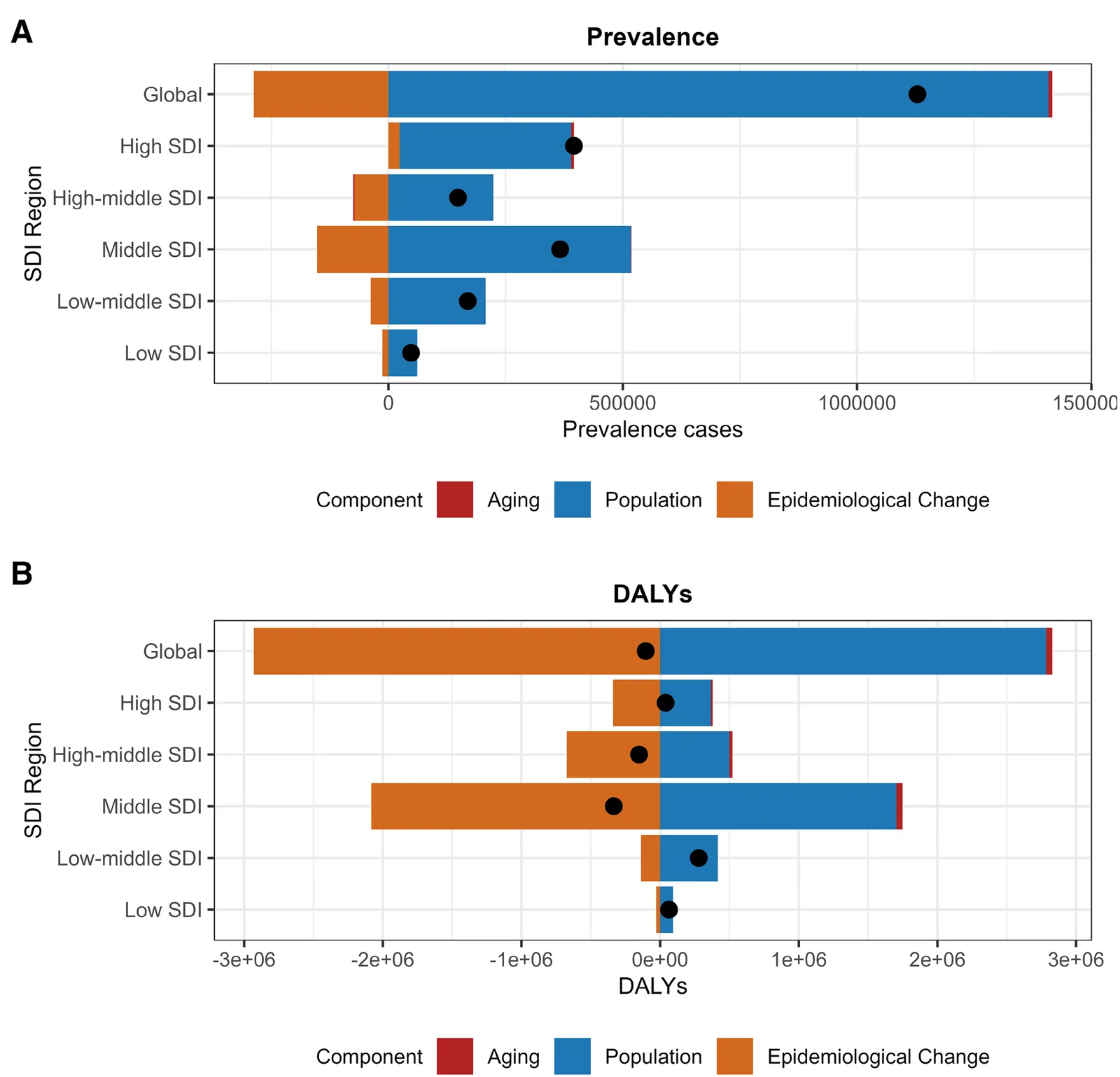
Decomposition of prevalence and DALYs of subarachnoid hemorrhage in postmenopausal women from 1990 to 2021 across SDI regions. (A) Contribution of aging, population growth, and epidemiological change to the overall change in prevalence. (B) Contribution of aging, population growth, and epidemiological change to the overall change in DALYs. Black dots indicate the net overall differences in each SDI region. DALYs = disability-adjusted life years, SDI = sociodemographic index.

Regional heterogeneity was stark. In high-SDI regions, the absolute burden rose for all 4 indicators; uniquely, the epidemiological component itself increased prevalence (+23,875 cases), and although death and DALY rates fell, demographic pressure still produced net increases in deaths (+4144) and DALYs (+39,420). High-middle and middle-SDI regions showed the greatest gains, with risk reductions exceeding demographic pressures driving net declines in deaths and DALYs. Low-middle and low-SDI regions were increasing burden across all measures, driven by unchecked population growth with only modest epidemiological offsets, suggesting lagging prevention and treatment capacity.

### 3.7. Analysis of health inequality in the disease burden of SAH in postmenopausal women

Inequality analysis using the CI and the SII showed socioeconomic differences in the burden of SAH among women aged 55 years and older. Both indices used the same population weights and moved in the same direction for every outcome, although the direction of the inequality itself differed by outcome (Fig. 6, Fig. S5, Supplemental Digital Content 10, and Table S6, Supplemental Digital Content 11).

**Figure 6.**
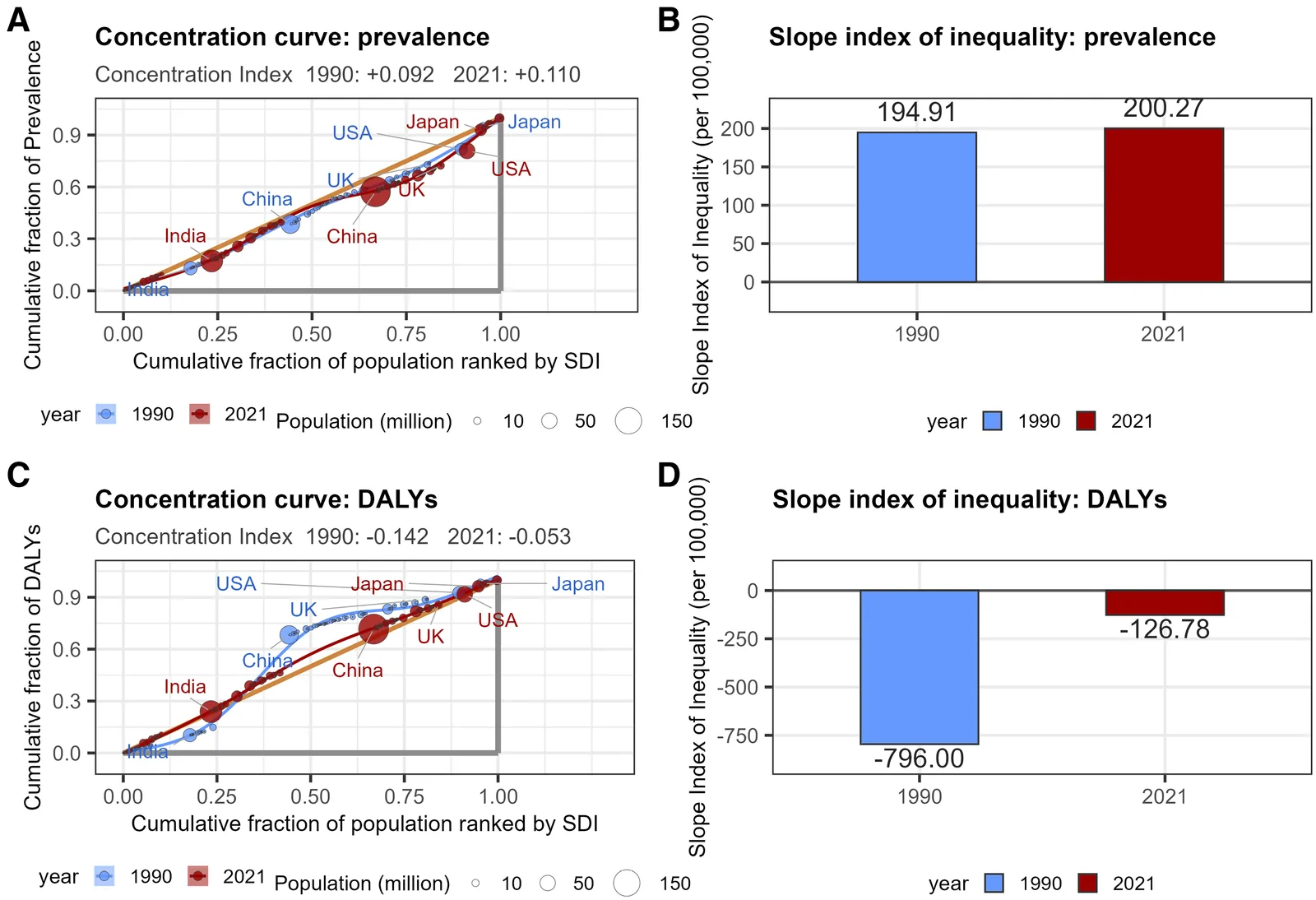
Socioeconomic inequalities in subarachnoid hemorrhage prevalence and DALYs among postmenopausal women aged ≥55 years, 1990 versus 2021. (A) Concentration curve of prevalence. (B) Slope index of inequality (SII) for prevalence. (C) Concentration curve of DALYs. (D) SII for DALYs. Concentration curves plot the cumulative share of the age-standardized burden against the cumulative share of the female population aged 55 years and older ranked by SDI; the SII panels show the absolute difference in the age-standardized rate across the SDI distribution, weighted by the same population. DALYs = disability-adjusted life years, SDI = sociodemographic index.

For incidence, the CI moved from −0.028 in 1990 to +0.043 in 2021, and for prevalence, it remained positive and increased slightly from +0.092 to +0.110, indicating that prevalent cases are concentrated in higher-SDI countries. For mortality, the CI moved from −0.164 to −0.049 and for DALYs, from −0.142 to −0.053, indicating that fatal and disabling burden remains concentrated in lower-SDI countries but that the relative gap narrowed rather than widened. On the absolute scale, the SII moved in the same direction as the CI for every outcome: from −6.14 to 6.05 for incidence, from 194.91 to 200.27 for prevalence, from −42.54 to −5.05 for mortality, and from −796.00 to −126.78 for DALYs. The absolute gap therefore narrowed for mortality and DALYs, where the SII moved toward zero, widened slightly for prevalence, and was approximately unchanged for incidence.

### 3.8. Sensitivity analysis of the age cut-point

Because menopausal status is not recorded in GBD, 55 years was used as an operational age proxy. To test whether the findings depend on that choice, the standardization and the trend estimation were repeated with cut-points of 50 and 60 years, holding the WHO World Standard Population fixed and varying only the age range entering the standardization. Global trends were in the same direction and statistically significant for all 4 outcomes at all 3 cut-points: the incidence rate EAPC was −1.65 at every cut-point, the prevalence rate EAPC was −0.63, −0.56 and −0.46, the mortality rate EAPC was −3.30, −3.31, and −3.31, and the DALY rate EAPC was −3.12, −3.15, and −3.17 for the 50+, 55+, and 60+ definitions, respectively (Table S7, Supplemental Digital Content 12, Fig. S6, Supplemental Digital Content 13). Across the 21 GBD regions, the 5 SDI regions, and the global level, the direction of the EAPC was identical at all 3 cut-points in 104 of 108 location-outcome combinations; in the 4 discordant combinations, the EAPCs were all close to zero (absolute value no greater than 0.51%), so the disagreement concerns whether a trend is flat or very slightly in one direction rather than a reversal of a substantive trend.

### 3.9. Risk-factor exposure and SAH burden

Among women aged 55 years and older, the SEV for smoking fell from 8.92 in 1990 to 5.98 in 2021 (−33.0%; EAPC: −1.17, 95% CI: −1.22 to −1.13), a decline close in magnitude to the 33.1% fall in the age-standardized incidence rate over the same period (EAPC: −1.65, 95% CI: −1.77 to −1.52). By contrast, the SEV for high systolic blood pressure changed little (47.90–46.16; −3.6%; EAPC: −0.26, 95% CI: −0.33 to −0.19), whereas the age-standardized death rate fell by 57.9% (EAPC: −3.31, 95% CI: −3.58 to −3.04) and the DALY rate by 56.5% (EAPC: −3.15, 95% CI: −3.40 to −2.89). Pooled across 704 location-years, the smoking SEV correlated positively with the SAH rate (incidence ρ = 0.23, *P* < .001; DALYs ρ = 0.21, *P* < .001), whereas the SEV for high systolic blood pressure correlated negatively with it (incidence ρ = −0.45, *P* < .001; DALYs ρ = −0.38, *P* < .001); these estimates were unchanged in direction and similar in magnitude after adjustment for the SDI and calendar year, and when the age-standardized SEV was substituted for the SEV of the 55 years and older group (Table S8, Supplemental Digital Content 14). Across the 21 GBD regions, the EAPC of exposure was not consistently associated with the EAPC of the SAH rate: the absolute value of ρ was below 0.50 in all 8 exposure-outcome pairs, and only one pair reached nominal significance (prevalence vs high systolic blood pressure, ρ = −0.49, *P* = .02; Fig. S7, Supplemental Digital Content 15).

## 4. Discussion

This paper is the first global assessment of the burden of SAH among postmenopausal women using GBD 2021 data. Throughout, “postmenopausal women” is used as a shorthand for the operational population of women aged 55 years and older, and not as a biologically verified group. Over the last 3 decades, absolute numbers of incident and prevalent cases have increased substantially, while deaths and DALYs have declined slightly, even as incidence, prevalence, mortality, and DALY rates fell.^[18]^ This paradoxically occurs because prevention and management improvements have reduced rates, while demographic changes such as population aging have increased the absolute burden.^[18]^ Age-specific analyses showed that the risk burden is concentrated in older age groups with women aged 70 years and older.^[25]^ Regional and socioeconomic disparities were evident: while prevalent cases were concentrated in higher-SDI countries, the burden of deaths and DALYs was concentrated in low-SDI countries.^[26]^ Decomposition analyses identified population aging and growth as the primary drivers of the rising burden, while improvement in epidemiological risks tended to counter some adverse trends in high-income countries.^[18]^ Inequality analyses using the CI and the SII showed that the absolute gaps between high- and low-SDI countries narrowed for deaths and DALYs but widened slightly for prevalence, while the direction of concentration differed by outcome: prevalent cases were concentrated in higher-SDI countries, whereas deaths and DALYs remained concentrated in lower-SDI countries.^[24]^

The divergence between case counts and age-specific rates in older women requires explanation. In our data, the absolute numbers of incident and prevalent cases peaked in the 55 to 59 age group and declined thereafter, whereas death counts continued to rise into the oldest age bands, and all 4 age-specific rates increased monotonically and reached their maxima at 85 years and older. Part of this pattern is arithmetic rather than epidemiological: rates are counts divided by the female population at risk, and the size of that population falls steeply with each successive age band, so a falling count can coexist with a rising rate. The count peak therefore reflects the shrinking number of women at the oldest ages rather than a reduction in per-woman risk. Beyond this denominator effect, 4 further mechanisms may amplify the divergence. First, aneurysm fragility increases with age: loss of elastin, collagen cross-linking, medial thinning, and cumulative exposure to hypertension alter the arterial wall so that aneurysms form, enlarge, and rupture more readily in older women.^[5,26–29]^ Second, case fatality rises with age and is higher in women, so each additional case at older ages contributes a disproportionate share of deaths.^[8,30,31]^ Third, ascertainment and survival bias act in different directions across settings. Where death occurs before hospital admission, a sudden fatal neurological event may never be recorded as SAH, and where emergency neuroimaging is scarce, such deaths are more readily attributed to cardiac causes or to nonspecific stroke; conversely, where computed tomography (CT) is readily available, it may be applied to older patients with nonspecific presentations, increasing diagnosis at older ages.^[32]^ Fourth, diagnostic under-investigation is plausibly more common in frail, nonhospitalized elderly women, and access to aneurysm repair is also skewed by socioeconomic position and by decisions about treatment intensity near the end of life.^[16]^ Taken together, these mechanisms imply that the rising rates we observe in the oldest age groups reflect both a genuine increase in per-woman risk and an ascertainment pattern that favors detection among older women who reach the hospital. Because GBD estimates are ecological and rest on modeled inputs, we cannot separate these mechanisms; distinguishing them will require neuroimaging-confirmed, individual-level, or registry-linked data.^[26,33]^

Our results complement previous work on stroke epidemiology and SAH burden. In previous GBD analyses, global age-standardized stroke incidence and death rates declined in high-income regions, due to advances in aneurysm repair, neurocritical care, and control of hypertension and smoking.^[34,35]^ We confirm those results and emphasize the demographic drivers: despite standardized rate decreases, absolute case counts rose due to population aging and growth.^[34]^ With regard to postmenopausal women, we identify a distinct biologically vulnerable group of clear demographic importance.

These exposure analyses, however, do not support attributing the decline in SAH burden primarily to cardiovascular risk-factor control. The 33% fall in smoking exposure among women aged 55 years and older closely parallels the 33% fall in incidence, so some contribution of tobacco control to the falling incidence is plausible. Yet, exposure to high systolic blood pressure was almost unchanged (−3.6%) while the death rate fell by 58% and the DALY rate by 57%; a discordance of this size cannot be accounted for by blood pressure control. The inverse cross-sectional association between blood pressure exposure and SAH rates should not be read as a protective effect: it most plausibly reflects the co-distribution of modeled exposure with case ascertainment and vital registration, because several of the regions with the highest blood pressure exposure are also those in which sudden deaths are least completely recorded. Taken together, these ecological associations do not demonstrate a causal pathway, and the fall in SAH burden is at least as consistent with improvements in diagnosis, acute management, and access to aneurysm repair as with population-level risk-factor control.

Clinical and genetic data further explain our results. Early menopause, shorter reproductive lifespan, and lower estrogen exposure are associated with a higher risk of aneurysmal SAH.^[9,11]^ Postmenopausal declines of estradiol increase vascular aging, impair endothelial function, and increase inflammation.^[27,28]^ Experimental studies confirm that estradiol protects the vascular barrier by stabilizing the extracellular matrix.^[36]^ Variations in estrogen receptor genes (PvuII polymorphism [rs2234693]) are also implicated in SAH risk.^[37]^ Women have worse functional outcomes and higher mortality after SAH than men.^[30,31]^ These biological and clinical observations are compatible with, but do not establish, a contribution of hormonal deficit and vascular aging to the population-level patterns described here; the ecological design of our analysis cannot link individual hormonal status to individual disease burden.

Regional differences in SAH burden reflect earlier results. In Europe, North America, and Japan, mortality has declined due to improved aneurysm screening, surgical clipping, and endovascular coiling.^[1]^ In South Asia, sub-Saharan Africa, and parts of Latin America, healthcare infrastructure and resources are limited and mortality rises or stagnates.^[25]^ Our inequality analyses reflect these reports, showing that fatal and disabling SAH burden remains concentrated in low-SDI settings where neurosurgical capacity is limited, poor access to imaging, and delayed diagnosis worsen outcomes.^[18]^ The high burden in these areas highlights the need for targeted interventions.^[18,33]^

We present the global SAH burden assessment of postmenopausal women in the context of stroke and women’s health research. We show that demographic shifts and epidemiological advances are interrelated: population growth and aging drive increases in absolute burden while medical advances reduce age-standardized rates (especially in high-SDI regions).^[34,38]^ We also show persistent socioeconomic differences, with the largest absolute gaps in fatal and disabling burden falling on low-SDI countries, although those gaps narrowed over the study period. These results have implications for clinical practice and public health policy. Because the analysis is ecological, these implications concern populations and health systems rather than individual patients, and they should not be read as individual-level risk predictions. In high-SDI countries, ongoing efforts to control modifiable risk factors (like hypertension and smoking) remain vital.^[29]^ Consider early screening for intracranial aneurysms in postmenopausal women with a family history of SAH or poorly controlled vascular risk factors. Health systems should prioritize postacute rehabilitation and cognitive care because long-term disability is common among survivors of SAH.^[39]^ In low-SDI countries, expanding health infrastructure, increasing neurosurgical capacity, and improving access to neuroimaging and intensive care are urgent priorities.^[32]^ Innovative approaches, such as mobile CT units or tele-neurosurgery consultation programs, could help reduce resource gaps.^[32]^

Because the burden is concentrated in this age group, gender- and age-specific strategies are warranted.^[9]^ Blood pressure monitoring and health education are established, low-risk measures and should be delivered through primary care. Hormone replacement therapy, by contrast, remains unresolved: experimental data suggest possible cardiovascular protection, but the available evidence is insufficient to support a preventive recommendation for cerebrovascular disease, and any evaluation should take place within adequately powered randomized trials.^[40–42]^ Public health programs should record reproductive and hormonal history, menopausal age, and reproductive lifespan so that these variables can be tested in cerebrovascular risk models.^[9,42]^

This paper used global data sets, standard methods, and advanced analytical methods such as decomposition and inequality analysis to evaluate the SAH burden of postmenopausal women by age, region, and socioeconomic factors. Several limitations should be noted. First, menopausal status was approximated by age 55 years and older; this operational proxy does not account for individual variation in the timing of menopause, or for premature and early menopause, and may therefore result in misclassification or underestimation. Second, GBD estimates for many low-SDI settings are derived largely from modeled predictions and extrapolation from neighboring regions rather than from complete vital registration or autopsy-confirmed diagnosis. In sub-Saharan Africa, parts of Latin America and India, civil registration and vital statistics coverage, the quality of death certification, and access to CT or other neurovascular imaging remain limited. Sudden deaths from aneurysmal SAH occurring outside a hospital may therefore go unrecorded, and SAH may be misclassified as nonspecific stroke or as a noncerebrovascular cause of death. These constraints are likely to widen the 95% UIs for the counts and rates we report in these regions, probably bias absolute numbers downward, and leave age-standardized trends comparatively more stable. The magnitude of this heterogeneity is visible in the UIs themselves: in 2021, the age-standardized death rate was 11.29 per 100,000 (95% UI: 5.29–29.01) in Central Sub-Saharan Africa, an upper bound 2.57 times the point estimate, and 16.70 (95% UI: 12.03–24.71) in South Asia, whereas the corresponding value in Western Europe was 9.64 (95% UI: 8.19–10.61), an upper bound only 1.10 times the point estimate (Table S1, Supplemental Digital Content 1). Because our decomposition and inequality estimates are built from the same modeled inputs, the regional contrasts we report should be interpreted as directional rather than as precise effect sizes. Third, we did not include individual-level risk factors such as blood pressure, smoking status, body mass index, or genetic predisposition, which limits causal inference about pathways; the risk-factor exposure analysis we added operates at the level of populations and cannot be linked to individual exposures. Fourth, a single age threshold of 55 years ignores the substantial geographic and socioeconomic variation in the age at natural menopause, which differs by region and varies with education, parity, smoking, and body mass index. With a uniform threshold, populations in which menopause occurs earlier contribute a larger proportion of women who are genuinely postmenopausal, whereas populations in which it occurs later contribute a larger proportion who are still premenopausal; the prevalence of surgical menopause after hysterectomy or bilateral oophorectomy also differs between countries.^[9,20]^ Because menopausal status is not recorded in GBD, cross-country comparisons of the postmenopausal burden are therefore affected by differential misclassification of the exposure proxy, and the regional contrasts we report should be read with this limitation in mind. Finally, this is an ecological analysis of group-level estimates; it cannot support inference about individual-level associations, and our findings should be read as ecological associations rather than as evidence of individual risk (ecological fallacy).

Future work should include clinical, epidemiological, and biological data to clarify linkages between menopause, estrogen deficit, and cerebrovascular disease. Large prospective cohort studies using reproductive history, hormone levels, genetic markers, and lifestyle factors are needed to better identify risk and elucidate mechanisms. Mendelian randomization and multiomics approaches could help to elucidate the causal pathways between sex hormones, cerebrovascular health, and aneurysm rupture risk.^[43]^ Randomized controlled trials of preventive measures (e.g., antihypertensive therapy or hormone replacement therapy in postmenopausal women) should also be conducted in order to assess their efficacy and safety in reducing SAH incidence and outcomes.

From a health-policy perspective, strategies to reduce the SAH burden should be tailored to the country context. High-SDI settings require strengthening primary prevention and enhancing screening of high-risk subgroups. Low-SDI settings require capacity building for neurosurgical care, investments in diagnostic technologies, and improved access to emergency and intensive care.^[44]^ Cross-country collaborations and global health initiatives should aim to reduce inequalities by providing technical assistance, training, and funding to resource-limited countries.^[45]^

## 5. Conclusion

In summary, although the age-standardized incidence, prevalence, mortality, and DALY rates all declined significantly over the study period, the absolute SAH burden among women aged 55 years and older increased because of population growth and aging, with women aged 85 years and older, and low-SDI countries for fatal and disabling burden, bearing the largest share. Translating these findings into practice, we propose 3 concrete priorities. First, primary care should implement targeted blood pressure screening and control protocols for women aged 55 years and older, particularly in low and low-middle SDI settings, where hypertension is the leading modifiable contributor to aneurysmal SAH. Second, health systems should establish standardized emergency neurosurgical referral pathways so that patients with suspected aneurysmal SAH reach a facility capable of surgical clipping or endovascular coiling as early as possible. Third, noninvasive neurovascular imaging, including CT angiography and magnetic resonance angiography, should be prioritized for scale-up in low-middle SDI regions, where limited diagnostic capacity rather than surgical technique currently constrains case ascertainment and outcome. These directives are specific and testable, and should be evaluated prospectively in health-services research.

## Acknowledgments

We extend our sincere gratitude to the GBD team for granting us access to their extensive database.

## Author contributions

**Conceptualization:** Mei Zhu.

**Data curation:** Mei Zhu.

**Formal analysis:** Mei Zhu.

**Investigation:** Mei Zhu.

**Methodology:** Mei Zhu, Zhihua Cheng.

**Resources:** Mei Zhu.

**Software:** Mei Zhu.

**Validation:** Mei Zhu, Zhihua Cheng.

**Visualization:** Mei Zhu.

**Funding acquisition:** Zhihua Cheng.

**Project administration:** Zhihua Cheng.

**Supervision:** Zhihua Cheng.

**Writing – original draft:** Mei Zhu.

**Writing – review & editing:** Zhihua Cheng.
































