# Supplementary material for: Global burden and trends of subarachnoid hemorrhage in women aged 55 years and older: A repeated cross-sectional analysis of the GBD 2021 study
Source: Medicine (Baltimore). 2026 Nov 6;105(45):e51457. doi: 10.1097/MD.0000000000051457 (PMC13747508; doi:10.1097/MD.0000000000051457)
Supplement: Supplementary file 1 [file medi-105-e51457-s001.docx]

Table S1. Age-standardized rates of incidence, prevalence, DALYs and deaths (95% UI) and EAPC (95% CI) of SAH-related indicators among postmenopausal women by GBD region, 1990–2021

| Location | **Incidence** **(95% UI)** | | | **Prevalence (95% UI)** | | | **DALYs (95% UI)** | | | **Deaths (95% UI)** | | |
| --- | --- | --- | --- | --- | --- | --- | --- | --- | --- | --- | --- | --- |
|  | **1990** | **2021** | **EAPC** | **1990** | **2021** | **EAPC** | **1990** | **2021** | **EAPC** | **1990** | **2021** | **EAPC** |
| Global | 34.39 (24.10, 47.71) | 22.99 (16.61, 31.02) | -1.65 (-1.77, -1.52) | 353.63 (299.81, 413.99) | 301.73 (263.73, 345.08) | -0.56 (-0.58, -0.53) | 898.63 (627.81, 1116.06) | 390.85 (342.53, 450.60) | -3.15 (-3.40, -2.89) | 39.86 (26.35, 50.32) | 16.78 (14.28, 19.85) | -3.31 (-3.58, -3.04) |
| Andean Latin America | 43.68 (31.49, 58.49) | 34.32 (25.49, 45.02) | -0.78 (-0.86, -0.70) | 715.10 (655.88, 780.25) | 583.40 (539.17, 625.70) | -0.76 (-0.80, -0.72) | 809.12 (563.90, 1129.26) | 667.42 (507.70, 868.63) | -0.79 (-0.97, -0.62) | 32.16 (21.40, 47.19) | 27.64 (20.40, 36.65) | -0.63 (-0.82, -0.45) |
| Australasia | 26.35 (19.72, 34.56) | 19.07 (13.54, 26.09) | -1.06 (-1.14, -0.97) | 345.65 (313.41, 381.03) | 310.13 (284.34, 337.32) | -0.31 (-0.36, -0.26) | 500.54 (428.91, 576.76) | 250.71 (207.49, 293.12) | -2.31 (-2.42, -2.20) | 20.84 (17.59, 24.25) | 10.64 (8.48, 12.52) | -2.25 (-2.36, -2.13) |
| Caribbean | 30.56 (21.76, 41.32) | 26.72 (19.57, 35.38) | -0.52 (-0.56, -0.48) | 526.22 (484.63, 569.93) | 465.89 (431.07, 500.25) | -0.44 (-0.46, -0.41) | 606.17 (445.59, 790.38) | 466.63 (347.05, 626.71) | -0.85 (-0.92, -0.78) | 23.69 (17.15, 31.30) | 18.19 (13.32, 24.78) | -0.87 (-0.94, -0.80) |
| Central Asia | 23.78 (17.49, 31.65) | 23.18 (18.23, 29.12) | 0.10 (0.04, 0.16) | 224.45 (199.07, 251.47) | 205.32 (185.10, 226.84) | -0.33 (-0.34, -0.32) | 385.47 (330.80, 458.24) | 430.54 (379.78, 483.21) | 0.36 (0.17, 0.55) | 16.09 (13.59, 19.71) | 19.93 (17.44, 22.47) | 0.85 (0.62, 1.07) |
| Central Europe | 23.38 (17.73, 30.49) | 18.45 (14.50, 23.09) | -1.02 (-1.11, -0.93) | 227.65 (199.45, 258.30) | 174.86 (155.04, 196.81) | -0.97 (-1.03, -0.90) | 493.84 (448.22, 548.04) | 312.73 (278.36, 347.42) | -1.71 (-2.01, -1.41) | 19.66 (17.72, 21.84) | 13.34 (11.74, 14.93) | -1.46 (-1.78, -1.13) |
| Central Latin America | 33.36 (24.15, 45.33) | 33.49 (24.79, 44.01) | -0.01 (-0.08, 0.05) | 603.94 (542.55, 671.36) | 541.91 (490.75, 598.27) | -0.39 (-0.41, -0.36) | 469.79 (428.99, 513.08) | 500.08 (434.26, 570.29) | 0.24 (0.04, 0.44) | 18.38 (16.67, 20.14) | 20.80 (17.73, 23.82) | 0.52 (0.29, 0.76) |
| Central Sub-Saharan Africa | 19.24 (12.68, 27.64) | 18.06 (11.98, 25.91) | -0.26 (-0.32, -0.19) | 317.42 (281.94, 354.49) | 302.27 (271.94, 334.26) | -0.18 (-0.19, -0.16) | 325.08 (171.85, 714.43) | 292.19 (158.81, 670.63) | -0.39 (-0.47, -0.31) | 12.44 (5.77, 30.35) | 11.29 (5.29, 29.01) | -0.36 (-0.45, -0.27) |
| East Asia | 58.62 (39.83, 82.48) | 21.82 (15.27, 30.04) | -3.92 (-4.27, -3.58) | 329.24 (255.67, 413.89) | 211.29 (173.78, 255.26) | -1.55 (-1.66, -1.45) | 2369.59 (1022.90, 3078.35) | 395.52 (282.95, 522.79) | -6.87 (-7.62, -6.12) | 120.47 (48.70, 157.94) | 18.92 (12.85, 25.66) | -7.17 (-7.96, -6.36) |
| Eastern Europe | 23.37 (15.99, 33.09) | 22.20 (15.76, 30.33) | -0.43 (-0.79, -0.07) | 219.43 (176.96, 268.56) | 214.70 (177.70, 257.93) | -0.07 (-0.11, -0.03) | 436.19 (395.38, 482.73) | 407.63 (358.82, 454.28) | -0.97 (-1.89, -0.04) | 19.27 (17.23, 21.53) | 19.02 (16.59, 21.30) | -0.80 (-1.92, 0.33) |
| Eastern Sub-Saharan Africa | 21.47 (14.07, 31.19) | 17.15 (11.51, 24.55) | -0.91 (-0.98, -0.84) | 343.86 (300.43, 392.25) | 281.09 (251.39, 313.65) | -0.71 (-0.73, -0.68) | 326.15 (182.83, 802.54) | 235.01 (136.68, 571.64) | -1.16 (-1.22, -1.10) | 12.19 (5.86, 34.15) | 8.71 (4.32, 24.18) | -1.17 (-1.24, -1.11) |
| High-income Asia Pacific | 69.85 (48.52, 97.56) | 52.92 (38.10, 71.25) | -1.01 (-1.11, -0.91) | 853.53 (713.28, 1020.33) | 882.40 (771.52, 1015.59) | 0.15 (0.12, 0.19) | 1031.12 (916.04, 1163.74) | 460.47 (391.83, 526.07) | -2.88 (-2.97, -2.79) | 41.71 (36.09, 47.71) | 16.27 (12.95, 18.78) | -3.38 (-3.51, -3.25) |
| High-income North America | 22.51 (15.22, 32.09) | 19.48 (13.55, 27.06) | -0.64 (-0.80, -0.47) | 342.98 (288.82, 406.41) | 360.52 (316.24, 409.62) | 0.04 (-0.02, 0.11) | 421.97 (393.27, 450.57) | 330.80 (299.78, 358.31) | -0.94 (-1.08, -0.79) | 16.17 (14.79, 17.20) | 13.63 (11.92, 14.77) | -0.63 (-0.81, -0.45) |
| North Africa and Middle East | 22.08 (15.30, 30.54) | 15.12 (11.09, 20.15) | -1.48 (-1.57, -1.39) | 271.64 (239.11, 305.78) | 211.66 (190.94, 232.71) | -0.91 (-0.96, -0.86) | 486.65 (278.27, 931.14) | 266.40 (199.65, 353.26) | -2.05 (-2.15, -1.94) | 21.72 (11.65, 44.25) | 12.11 (8.79, 16.42) | -1.86 (-2.00, -1.72) |
| Oceania | 52.27 (37.82, 70.15) | 42.28 (31.53, 55.26) | -0.83 (-0.88, -0.78) | 460.63 (404.90, 518.00) | 411.14 (368.12, 453.72) | -0.42 (-0.44, -0.40) | 1276.24 (780.48, 2024.58) | 906.79 (580.36, 1420.68) | -1.20 (-1.24, -1.16) | 58.75 (34.76, 95.82) | 41.16 (25.34, 66.61) | -1.25 (-1.29, -1.21) |
| South Asia | 29.58 (19.97, 42.08) | 20.32 (14.08, 28.04) | -1.56 (-1.70, -1.43) | 233.19 (184.79, 290.34) | 193.63 (158.36, 235.15) | -0.64 (-0.70, -0.57) | 566.05 (294.94, 1078.11) | 419.96 (314.46, 588.13) | -1.06 (-1.17, -0.95) | 22.67 (11.17, 47.08) | 16.70 (12.03, 24.71) | -1.07 (-1.17, -0.97) |
| Southeast Asia | 39.72 (27.98, 54.50) | 30.16 (21.94, 40.44) | -1.09 (-1.19, -0.99) | 426.94 (369.60, 492.08) | 361.68 (318.10, 411.65) | -0.60 (-0.63, -0.57) | 873.71 (584.13, 1321.55) | 569.87 (437.23, 828.50) | -1.52 (-1.62, -1.41) | 38.71 (24.69, 63.19) | 25.16 (18.50, 39.67) | -1.53 (-1.65, -1.41) |
| Southern Latin America | 46.15 (34.01, 61.47) | 30.98 (23.23, 40.30) | -1.43 (-1.54, -1.33) | 496.02 (420.07, 577.13) | 410.05 (342.89, 501.49) | -0.54 (-0.61, -0.47) | 905.57 (760.22, 1075.79) | 419.52 (359.36, 482.96) | -2.26 (-2.42, -2.11) | 36.48 (30.07, 43.98) | 16.63 (13.92, 19.31) | -2.27 (-2.48, -2.06) |
| Southern Sub-Saharan Africa | 15.63 (10.30, 22.58) | 14.60 (9.89, 20.53) | -0.29 (-0.46, -0.12) | 274.42 (234.03, 321.46) | 243.51 (210.55, 281.51) | -0.45 (-0.48, -0.42) | 168.24 (131.83, 234.68) | 203.81 (163.74, 261.11) | 1.00 (0.61, 1.38) | 6.03 (4.48, 9.10) | 7.79 (6.02, 10.31) | 1.22 (0.77, 1.69) |
| Tropical Latin America | 31.61 (21.29, 45.45) | 27.83 (19.35, 38.65) | -0.68 (-0.77, -0.59) | 489.83 (399.10, 595.81) | 373.56 (307.54, 449.72) | -1.11 (-1.23, -0.99) | 676.90 (624.56, 730.62) | 574.51 (522.03, 622.79) | -0.55 (-0.82, -0.28) | 25.29 (23.09, 27.54) | 23.11 (20.60, 25.20) | -0.24 (-0.54, 0.06) |
| Western Europe | 23.72 (17.32, 31.89) | 18.32 (13.41, 24.39) | -0.91 (-0.97, -0.85) | 348.37 (311.99, 387.58) | 308.33 (280.25, 338.36) | -0.40 (-0.42, -0.39) | 384.66 (354.25, 414.13) | 230.15 (203.75, 252.76) | -1.59 (-1.74, -1.45) | 15.81 (14.34, 17.12) | 9.64 (8.19, 10.61) | -1.47 (-1.67, -1.27) |
| Western Sub-Saharan Africa | 12.68 (8.22, 18.75) | 10.42 (6.85, 15.15) | -0.79 (-0.86, -0.72) | 260.09 (229.52, 294.21) | 229.42 (206.37, 254.67) | -0.45 (-0.48, -0.43) | 282.70 (178.78, 596.87) | 214.31 (146.49, 437.82) | -0.96 (-1.03, -0.88) | 11.14 (6.32, 25.58) | 8.29 (5.16, 18.79) | -1.02 (-1.09, -0.94) |

Note. Rates are expressed per 100,000 women and were age-standardized to the WHO World Standard Population (WHO 2000–2025), renormalized within ages 55 years and older. Abbreviations: CI, confidence interval; EAPC, estimated annual percentage change; UI, uncertainty interval.
