# Supplementary material for: Global burden and trends of subarachnoid hemorrhage in women aged 55 years and older: A repeated cross-sectional analysis of the GBD 2021 study
Source: Medicine (Baltimore). 2026 Nov 6;105(45):e51457. doi: 10.1097/MD.0000000000051457 (PMC13747508; doi:10.1097/MD.0000000000051457)
Supplement: Supplementary file 2 [file medi-105-e51457-s002.docx]

Table S2. Case counts (95% UI) and EAPC (95% CI) for SAH outcomes in postmenopausal women across GBD countries, 1990–2021

| **Location** | **Incidence (95% UI)** | | | **Prevalence (95% UI)** | | | **DALYs (95% UI)** | | | **Deaths (95% UI)** | | |
| --- | --- | --- | --- | --- | --- | --- | --- | --- | --- | --- | --- | --- |
|  | **1990** | **2021** | **EAPC** | **1990** | **2021** | **EAPC** | **1990** | **2021** | **EAPC** | **1990** | **2021** | **EAPC** |
| Afghanistan | 125 (98, 159) | 155 (129, 187) | 0.22 (0.03, 0.41) | 1491 (1291, 1694) | 1538 (1360, 1706) | -0.33 (-0.49, -0.17) | 3639 (1012, 8202) | 3592 (1097, 6495) | -0.56 (-0.86, -0.26) | 143 (33, 342) | 152 (41, 295) | -0.15 (-0.41, 0.11) |
| Albania | 34 (29, 40) | 85 (74, 96) | 3.14 (3.03, 3.26) | 345 (314, 378) | 710 (644, 769) | 2.32 (2.27, 2.38) | 592 (462, 779) | 1002 (653, 1468) | 2.08 (1.81, 2.35) | 27 (21, 36) | 51 (31, 77) | 2.58 (2.25, 2.91) |
| Algeria | 233 (184, 286) | 440 (356, 541) | 1.67 (1.51, 1.82) | 3173 (2868, 3476) | 6786 (6229, 7294) | 2.36 (2.24, 2.47) | 3562 (1972, 6673) | 6905 (4508, 10646) | 2.08 (1.98, 2.19) | 145 (71, 282) | 310 (181, 512) | 2.51 (2.41, 2.60) |
| American Samoa | 1 (1, 1) | 1 (1, 2) | 2.49 (2.42, 2.57) | 8 (7, 8) | 17 (16, 18) | 2.55 (2.51, 2.59) | 12 (9, 15) | 25 (19, 32) | 2.29 (2.10, 2.49) | 0 (0, 1) | 1 (1, 1) | 2.42 (2.16, 2.68) |
| Andorra | 1 (1, 1) | 3 (2, 3) | 2.98 (2.87, 3.10) | 17 (15, 18) | 41 (37, 44) | 2.56 (2.37, 2.76) | 20 (12, 30) | 34 (24, 47) | 1.72 (1.54, 1.90) | 1 (0, 1) | 2 (1, 2) | 2.36 (2.10, 2.62) |
| Angola | 60 (45, 78) | 166 (128, 214) | 3.16 (3.02, 3.30) | 967 (878, 1057) | 2899 (2659, 3143) | 3.59 (3.45, 3.72) | 1002 (565, 2183) | 2739 (1758, 5493) | 3.00 (2.84, 3.16) | 35 (18, 83) | 95 (56, 207) | 2.98 (2.81, 3.14) |
| Antigua and Barbuda | 2 (1, 2) | 3 (2, 3) | 1.62 (1.35, 1.90) | 27 (25, 29) | 46 (42, 49) | 1.85 (1.62, 2.07) | 27 (24, 31) | 40 (36, 44) | 1.12 (0.80, 1.44) | 1 (1, 1) | 2 (1, 2) | 0.55 (0.21, 0.88) |
| Argentina | 1515 (1252, 1865) | 1603 (1344, 1916) | -0.03 (-0.16, 0.11) | 15678 (13522, 18159) | 20519 (17118, 26181) | 0.88 (0.84, 0.91) | 30676 (26800, 34811) | 21349 (19331, 23641) | -0.91 (-1.24, -0.59) | 1297 (1113, 1475) | 924 (824, 1018) | -0.77 (-1.15, -0.39) |
| Armenia | 65 (54, 76) | 65 (56, 76) | -0.34 (-0.54, -0.14) | 607 (550, 665) | 728 (666, 793) | 0.32 (0.17, 0.47) | 1165 (989, 1346) | 879 (773, 997) | -1.14 (-1.61, -0.67) | 50 (42, 58) | 42 (37, 47) | -0.57 (-1.13, -0.02) |
| Australia | 478 (416, 556) | 839 (704, 999) | 1.90 (1.81, 2.00) | 6334 (5854, 6852) | 13055 (12172, 13954) | 2.49 (2.44, 2.54) | 8725 (7928, 9572) | 10466 (9112, 11694) | 0.62 (0.49, 0.75) | 397 (360, 435) | 529 (434, 589) | 0.94 (0.81, 1.06) |
| Austria | 498 (435, 575) | 467 (392, 549) | 0.34 (0.10, 0.58) | 8651 (7407, 9340) | 10668 (9875, 11338) | 0.77 (0.63, 0.91) | 4987 (4546, 5462) | 5146 (4459, 5804) | 0.31 (0.17, 0.45) | 206 (186, 225) | 233 (189, 265) | 0.66 (0.45, 0.86) |
| Azerbaijan | 86 (63, 114) | 134 (108, 168) | 1.40 (1.11, 1.70) | 953 (847, 1073) | 1808 (1631, 2002) | 1.75 (1.32, 2.17) | 815 (572, 1217) | 1339 (903, 2006) | 1.14 (0.94, 1.34) | 28 (17, 47) | 45 (24, 74) | 1.29 (1.12, 1.45) |
| Bahamas | 4 (3, 5) | 9 (7, 11) | 2.67 (2.54, 2.80) | 60 (55, 65) | 155 (144, 166) | 3.03 (2.94, 3.12) | 55 (47, 66) | 119 (98, 143) | 2.44 (2.31, 2.58) | 2 (2, 3) | 5 (4, 6) | 2.27 (2.11, 2.42) |
| Bahrain | 2 (1, 2) | 5 (4, 7) | 3.80 (3.46, 4.15) | 24 (22, 27) | 101 (92, 109) | 4.85 (4.46, 5.24) | 60 (45, 83) | 114 (86, 153) | 1.00 (0.59, 1.41) | 2 (2, 3) | 4 (3, 6) | 0.81 (0.44, 1.18) |
| Bangladesh | 1091 (873, 1368) | 3447 (2863, 4239) | 3.99 (3.84, 4.13) | 11771 (10535, 13227) | 34357 (30932, 38021) | 3.89 (3.58, 4.20) | 32623 (15996, 69975) | 73058 (47422, 127434) | 3.11 (2.89, 3.34) | 1362 (637, 3102) | 3171 (1990, 5803) | 3.20 (3.00, 3.40) |
| Barbados | 9 (7, 11) | 13 (11, 16) | 1.33 (1.03, 1.63) | 137 (127, 147) | 223 (206, 239) | 1.66 (1.44, 1.88) | 134 (120, 153) | 222 (177, 273) | 1.92 (1.52, 2.32) | 6 (6, 7) | 10 (8, 12) | 1.50 (1.12, 1.88) |
| Belarus | 328 (274, 397) | 344 (296, 404) | 0.11 (0.01, 0.22) | 2885 (2560, 3197) | 3259 (2959, 3535) | 0.26 (0.14, 0.38) | 5061 (3858, 6723) | 5118 (4252, 5953) | -0.54 (-0.75, -0.34) | 221 (163, 303) | 252 (209, 294) | -0.02 (-0.20, 0.17) |
| Belgium | 290 (239, 350) | 392 (337, 464) | 0.83 (0.50, 1.17) | 3978 (3646, 4307) | 5058 (4656, 5441) | 0.65 (0.44, 0.85) | 4266 (3816, 4660) | 4947 (4277, 5544) | 0.77 (0.50, 1.04) | 199 (176, 218) | 264 (213, 302) | 1.31 (0.94, 1.69) |
| Belize | 2 (2, 3) | 5 (4, 6) | 2.92 (2.66, 3.18) | 34 (31, 37) | 95 (88, 101) | 3.32 (3.06, 3.59) | 27 (23, 33) | 72 (62, 82) | 2.98 (2.76, 3.20) | 1 (1, 1) | 3 (2, 3) | 2.75 (2.53, 2.97) |
| Benin | 22 (17, 30) | 50 (39, 67) | 2.49 (2.40, 2.57) | 434 (397, 471) | 1031 (944, 1111) | 2.73 (2.65, 2.82) | 595 (347, 1186) | 1168 (712, 2457) | 2.23 (2.09, 2.37) | 24 (13, 51) | 46 (26, 107) | 2.20 (2.08, 2.33) |
| Bermuda | 2 (1, 2) | 2 (2, 3) | 0.96 (0.83, 1.10) | 33 (30, 35) | 58 (55, 62) | 1.98 (1.91, 2.06) | 23 (19, 27) | 23 (20, 28) | -0.29 (-0.57, -0.02) | 1 (1, 1) | 1 (1, 1) | -0.36 (-0.68, -0.03) |
| Bhutan | 6 (5, 7) | 10 (9, 12) | 1.70 (1.61, 1.79) | 55 (49, 62) | 109 (97, 120) | 2.22 (2.19, 2.26) | 137 (60, 248) | 197 (130, 308) | 0.98 (0.85, 1.11) | 5 (2, 10) | 8 (5, 13) | 1.32 (1.21, 1.44) |
| Bolivia (Plurinational State of) | 159 (130, 193) | 375 (313, 448) | 2.85 (2.78, 2.92) | 2132 (1942, 2339) | 4992 (4585, 5420) | 2.85 (2.82, 2.89) | 4722 (2579, 7310) | 9499 (6037, 13732) | 2.40 (2.32, 2.47) | 193 (98, 307) | 405 (255, 598) | 2.61 (2.51, 2.71) |
| Bosnia and Herzegovina | 114 (95, 138) | 154 (135, 176) | 0.92 (0.81, 1.02) | 980 (870, 1089) | 1123 (1014, 1229) | 0.16 (0.06, 0.26) | 3081 (1963, 4493) | 2013 (1479, 2599) | -1.94 (-2.19, -1.69) | 121 (77, 189) | 93 (65, 124) | -1.34 (-1.58, -1.11) |
| Botswana | 10 (7, 12) | 23 (18, 29) | 2.68 (2.60, 2.76) | 144 (131, 157) | 359 (333, 390) | 2.84 (2.77, 2.91) | 117 (78, 218) | 192 (142, 326) | 1.99 (1.58, 2.40) | 4 (3, 9) | 7 (4, 12) | 1.89 (1.41, 2.36) |
| Brazil | 2480 (1961, 3184) | 6720 (5535, 8223) | 3.05 (2.97, 3.14) | 38504 (32493, 45583) | 89063 (75918, 104613) | 2.54 (2.40, 2.69) | 53697 (50701, 57048) | 135890 (125279, 143866) | 3.09 (2.85, 3.34) | 1973 (1858, 2102) | 5646 (5090, 6004) | 3.57 (3.28, 3.86) |
| Brunei Darussalam | 7 (6, 9) | 19 (15, 23) | 2.66 (2.43, 2.88) | 78 (71, 86) | 232 (215, 250) | 3.60 (3.39, 3.82) | 139 (98, 182) | 249 (200, 310) | 1.73 (1.48, 1.99) | 6 (4, 7) | 9 (7, 12) | 1.42 (1.16, 1.68) |
| Bulgaria | 206 (173, 241) | 329 (293, 367) | 1.80 (1.67, 1.93) | 2139 (1934, 2379) | 2309 (2077, 2558) | 0.41 (0.34, 0.47) | 4175 (3603, 4787) | 5999 (5209, 6903) | 1.43 (1.23, 1.64) | 159 (136, 183) | 308 (268, 354) | 2.52 (2.27, 2.78) |
| Burkina Faso | 42 (31, 57) | 80 (61, 105) | 2.06 (1.97, 2.15) | 908 (828, 996) | 1766 (1632, 1904) | 2.19 (2.11, 2.27) | 655 (368, 1459) | 1285 (766, 2810) | 2.58 (2.46, 2.70) | 22 (11, 57) | 46 (23, 113) | 2.90 (2.73, 3.07) |
| Burundi | 48 (38, 62) | 71 (54, 93) | 0.97 (0.59, 1.34) | 781 (710, 852) | 1130 (1037, 1220) | 1.13 (0.78, 1.48) | 967 (427, 2252) | 1018 (470, 3011) | -0.32 (-0.78, 0.14) | 36 (14, 92) | 36 (13, 120) | -0.55 (-1.01, -0.10) |
| Cabo Verde | 3 (2, 4) | 5 (4, 7) | 1.37 (1.11, 1.63) | 67 (62, 73) | 120 (110, 128) | 1.66 (1.53, 1.80) | 43 (26, 112) | 69 (45, 151) | 1.45 (1.16, 1.75) | 2 (1, 5) | 3 (1, 7) | 1.54 (1.26, 1.82) |
| Cambodia | 162 (131, 196) | 394 (328, 475) | 2.87 (2.76, 2.98) | 1649 (1473, 1827) | 4346 (3966, 4744) | 3.29 (3.14, 3.45) | 4187 (2162, 6856) | 7498 (5257, 11694) | 1.80 (1.58, 2.01) | 169 (81, 300) | 311 (207, 536) | 1.88 (1.66, 2.10) |
| Cameroon | 47 (35, 63) | 115 (87, 150) | 2.78 (2.70, 2.87) | 945 (867, 1032) | 2414 (2237, 2607) | 2.98 (2.91, 3.05) | 1051 (690, 1994) | 2565 (1530, 5131) | 2.95 (2.84, 3.06) | 39 (25, 78) | 95 (52, 212) | 2.97 (2.84, 3.11) |
| Canada | 935 (777, 1118) | 1564 (1307, 1864) | 1.75 (1.69, 1.81) | 15011 (13830, 16086) | 26186 (24259, 27956) | 1.83 (1.76, 1.90) | 13923 (12779, 15179) | 18487 (16429, 20453) | 0.83 (0.73, 0.93) | 578 (526, 629) | 859 (730, 955) | 1.21 (1.06, 1.36) |
| Central African Republic | 20 (15, 26) | 36 (28, 46) | 1.78 (1.66, 1.89) | 296 (267, 329) | 551 (501, 607) | 1.94 (1.81, 2.08) | 416 (198, 901) | 702 (345, 1753) | 1.60 (1.49, 1.71) | 15 (6, 35) | 25 (11, 69) | 1.63 (1.54, 1.72) |
| Chad | 31 (24, 41) | 49 (37, 63) | 1.37 (1.27, 1.47) | 636 (576, 693) | 1013 (936, 1090) | 1.41 (1.30, 1.53) | 737 (398, 1724) | 1362 (773, 2803) | 1.99 (1.94, 2.05) | 28 (14, 73) | 51 (27, 113) | 1.92 (1.87, 1.96) |
| Chile | 325 (262, 409) | 765 (632, 911) | 3.09 (2.92, 3.26) | 3553 (3153, 3982) | 9493 (8212, 11500) | 3.65 (3.43, 3.88) | 5045 (4604, 5502) | 9683 (8760, 10677) | 2.48 (2.18, 2.77) | 193 (176, 209) | 429 (379, 475) | 2.96 (2.59, 3.33) |
| China | 39895 (32281, 49426) | 43238 (36260, 51735) | -0.55 (-0.98, -0.12) | 240245 (202059, 285882) | 405511 (350453, 474495) | 1.62 (1.38, 1.85) | 1703023 (751078, 2185813) | 782371 (563249, 1037555) | -3.81 (-4.69, -2.92) | 81343 (33121, 105080) | 38353 (26297, 52035) | -3.86 (-4.79, -2.92) |
| Colombia | 674 (555, 814) | 2505 (2106, 2989) | 4.31 (4.14, 4.48) | 11665 (10796, 12573) | 36577 (34214, 39083) | 3.73 (3.67, 3.78) | 11035 (10103, 12072) | 35661 (30402, 41136) | 3.97 (3.59, 4.36) | 431 (400, 464) | 1568 (1294, 1838) | 4.55 (4.10, 5.00) |
| Comoros | 3 (3, 4) | 7 (6, 10) | 2.30 (2.23, 2.38) | 59 (54, 65) | 136 (125, 147) | 2.63 (2.59, 2.68) | 59 (35, 125) | 107 (63, 238) | 1.71 (1.54, 1.87) | 2 (1, 5) | 4 (2, 9) | 1.76 (1.57, 1.94) |
| Congo | 19 (15, 24) | 34 (26, 45) | 1.63 (1.48, 1.78) | 304 (273, 332) | 593 (545, 638) | 2.04 (1.92, 2.17) | 367 (222, 790) | 647 (424, 1243) | 1.53 (1.39, 1.68) | 13 (7, 30) | 23 (14, 49) | 1.48 (1.34, 1.63) |
| Cook Islands | 0 (0, 0) | 1 (1, 1) | 1.19 (0.99, 1.40) | 5 (5, 6) | 10 (9, 10) | 2.00 (1.92, 2.08) | 7 (5, 10) | 7 (5, 10) | -0.23 (-0.50, 0.05) | 0 (0, 0) | 0 (0, 0) | -0.35 (-0.69, -0.02) |
| Costa Rica | 55 (46, 68) | 148 (123, 180) | 3.15 (3.05, 3.25) | 967 (898, 1033) | 2899 (2714, 3083) | 3.56 (3.51, 3.62) | 645 (575, 712) | 1901 (1637, 2143) | 3.49 (3.26, 3.72) | 25 (23, 29) | 80 (68, 91) | 3.69 (3.44, 3.93) |
| Croatia | 155 (134, 181) | 157 (140, 179) | -0.11 (-0.28, 0.06) | 1330 (1196, 1455) | 1215 (1102, 1331) | -0.50 (-0.59, -0.42) | 4304 (3855, 4685) | 2951 (2546, 3387) | -1.10 (-1.31, -0.89) | 180 (160, 197) | 160 (138, 183) | -0.11 (-0.36, 0.14) |
| Cuba | 243 (202, 293) | 405 (338, 494) | 1.41 (1.31, 1.51) | 4731 (4384, 5120) | 7946 (7401, 8460) | 1.54 (1.48, 1.60) | 3804 (3211, 4375) | 5607 (4826, 6438) | 0.95 (0.76, 1.14) | 152 (126, 177) | 236 (200, 271) | 1.10 (0.87, 1.34) |
| Cyprus | 27 (22, 32) | 40 (34, 47) | 0.76 (0.52, 1.00) | 306 (277, 331) | 516 (473, 556) | 1.60 (1.40, 1.80) | 548 (336, 790) | 466 (361, 594) | -0.80 (-1.02, -0.58) | 28 (15, 41) | 23 (17, 32) | -0.72 (-0.97, -0.48) |
| Czechia | 345 (296, 397) | 335 (292, 387) | -0.20 (-0.30, -0.09) | 3243 (2917, 3595) | 3573 (3289, 3868) | 0.56 (0.46, 0.66) | 8270 (7110, 9496) | 4661 (4120, 5283) | -1.71 (-2.00, -1.41) | 359 (311, 413) | 230 (197, 261) | -1.40 (-1.74, -1.07) |
| Côte d'Ivoire | 35 (26, 48) | 91 (68, 117) | 2.89 (2.77, 3.01) | 684 (628, 742) | 1867 (1725, 2003) | 3.05 (2.94, 3.16) | 709 (439, 1296) | 1877 (1170, 3961) | 3.45 (3.27, 3.63) | 25 (15, 50) | 68 (39, 151) | 3.64 (3.42, 3.87) |
| Democratic People's Republic of Korea | 731 (613, 891) | 1139 (963, 1354) | 1.00 (0.83, 1.16) | 5828 (5221, 6433) | 9784 (8829, 10768) | 1.34 (1.19, 1.49) | 18176 (11024, 25535) | 23583 (14927, 37729) | 0.53 (0.38, 0.69) | 805 (480, 1135) | 1100 (670, 1917) | 0.84 (0.74, 0.95) |
| Democratic Republic of the Congo | 265 (193, 356) | 613 (467, 789) | 2.55 (2.48, 2.62) | 4282 (3890, 4732) | 9835 (9023, 10671) | 2.50 (2.41, 2.60) | 4451 (2410, 9571) | 9602 (5004, 23477) | 2.37 (2.32, 2.42) | 153 (72, 356) | 347 (158, 944) | 2.54 (2.44, 2.64) |
| Denmark | 243 (207, 284) | 248 (210, 293) | 0.06 (-0.04, 0.17) | 3477 (3214, 3770) | 3740 (3460, 4029) | 0.39 (0.33, 0.45) | 4355 (3894, 4857) | 3335 (2985, 3663) | -0.83 (-1.00, -0.66) | 195 (170, 219) | 174 (148, 193) | -0.36 (-0.55, -0.16) |
| Djibouti | 2 (2, 3) | 8 (6, 10) | 4.30 (4.23, 4.38) | 33 (30, 36) | 134 (124, 145) | 4.56 (4.53, 4.59) | 29 (18, 63) | 102 (59, 219) | 4.01 (3.91, 4.11) | 1 (1, 2) | 3 (2, 8) | 3.98 (3.89, 4.08) |
| Dominica | 2 (1, 2) | 2 (1, 2) | -0.12 (-0.30, 0.07) | 28 (26, 31) | 31 (29, 34) | 0.14 (0.05, 0.23) | 32 (25, 41) | 30 (23, 37) | -0.38 (-0.59, -0.17) | 1 (1, 2) | 1 (1, 2) | -0.29 (-0.44, -0.14) |
| Dominican Republic | 82 (65, 101) | 276 (228, 332) | 4.13 (4.05, 4.21) | 1535 (1425, 1656) | 4766 (4436, 5110) | 3.71 (3.63, 3.79) | 1522 (1122, 2014) | 3808 (2721, 5236) | 3.33 (3.18, 3.48) | 60 (42, 82) | 149 (99, 210) | 3.41 (3.20, 3.62) |
| Ecuador | 188 (153, 230) | 627 (534, 759) | 4.24 (4.13, 4.35) | 3500 (3257, 3769) | 10443 (9778, 11115) | 3.63 (3.59, 3.68) | 2575 (2251, 2935) | 11745 (9541, 14330) | 5.23 (4.97, 5.49) | 94 (80, 110) | 519 (414, 638) | 5.94 (5.63, 6.24) |
| Egypt | 363 (282, 462) | 665 (532, 823) | 1.78 (1.64, 1.93) | 4935 (4406, 5433) | 9531 (8670, 10365) | 2.02 (1.85, 2.19) | 9518 (4173, 20454) | 12493 (7438, 21166) | 0.91 (0.69, 1.14) | 399 (159, 912) | 491 (274, 874) | 0.77 (0.54, 1.00) |
| El Salvador | 99 (82, 119) | 181 (153, 217) | 1.97 (1.82, 2.12) | 1631 (1513, 1749) | 3024 (2819, 3216) | 1.92 (1.87, 1.97) | 1467 (1101, 1973) | 2248 (1682, 2914) | 1.59 (1.24, 1.94) | 58 (42, 82) | 94 (67, 125) | 1.90 (1.53, 2.27) |
| Equatorial Guinea | 4 (3, 5) | 6 (5, 8) | 1.51 (1.27, 1.76) | 53 (47, 58) | 107 (98, 116) | 2.29 (2.18, 2.39) | 60 (33, 135) | 79 (43, 150) | 0.34 (-0.08, 0.76) | 2 (1, 5) | 3 (1, 6) | 0.10 (-0.36, 0.56) |
| Eritrea | 23 (17, 30) | 51 (39, 67) | 2.85 (2.77, 2.93) | 356 (325, 392) | 881 (811, 951) | 3.22 (3.13, 3.30) | 426 (211, 934) | 779 (463, 1692) | 2.42 (2.20, 2.65) | 14 (6, 34) | 27 (14, 65) | 2.53 (2.29, 2.77) |
| Estonia | 45 (37, 54) | 44 (37, 53) | -0.18 (-0.28, -0.08) | 556 (486, 628) | 443 (392, 492) | -0.71 (-0.78, -0.65) | 616 (537, 722) | 558 (471, 630) | -1.05 (-1.74, -0.35) | 26 (22, 30) | 32 (26, 36) | -0.05 (-0.87, 0.77) |
| Eswatini | 4 (3, 5) | 8 (6, 10) | 2.09 (1.86, 2.33) | 64 (58, 70) | 128 (118, 137) | 2.17 (2.07, 2.27) | 57 (40, 94) | 117 (75, 183) | 2.63 (2.10, 3.17) | 2 (1, 4) | 4 (3, 7) | 2.55 (2.04, 3.07) |
| Ethiopia | 352 (261, 457) | 475 (366, 608) | 0.52 (0.31, 0.72) | 5682 (4772, 6790) | 8001 (6984, 9290) | 1.01 (0.83, 1.18) | 5068 (2614, 12085) | 5668 (3219, 13770) | -0.05 (-0.26, 0.17) | 170 (72, 459) | 197 (92, 560) | 0.03 (-0.19, 0.25) |
| Fiji | 20 (17, 24) | 42 (35, 50) | 2.24 (2.18, 2.29) | 202 (183, 224) | 444 (409, 482) | 2.60 (2.58, 2.63) | 303 (229, 387) | 580 (436, 767) | 2.07 (1.97, 2.18) | 12 (9, 15) | 23 (16, 31) | 2.12 (1.99, 2.25) |
| Finland | 278 (249, 309) | 341 (292, 399) | 0.62 (0.39, 0.85) | 4886 (4513, 5243) | 7021 (6626, 7430) | 1.44 (1.26, 1.63) | 4772 (4170, 5422) | 4474 (3947, 5012) | -0.12 (-0.21, -0.03) | 202 (171, 232) | 209 (174, 235) | 0.09 (-0.04, 0.21) |
| France | 1442 (1224, 1689) | 2579 (2248, 2966) | 2.21 (2.10, 2.32) | 21514 (19863, 23139) | 38185 (35427, 40975) | 2.05 (1.96, 2.14) | 22749 (20327, 24862) | 28677 (24913, 32265) | 1.23 (0.93, 1.52) | 1199 (1044, 1316) | 1558 (1271, 1751) | 1.39 (1.02, 1.77) |
| Gabon | 10 (8, 13) | 13 (10, 17) | 0.48 (0.27, 0.70) | 168 (152, 184) | 246 (226, 266) | 0.98 (0.82, 1.14) | 130 (84, 248) | 173 (109, 306) | 0.59 (0.43, 0.76) | 5 (3, 10) | 6 (3, 11) | 0.54 (0.35, 0.72) |
| Gambia | 3 (2, 4) | 9 (7, 12) | 3.45 (3.30, 3.60) | 64 (59, 70) | 190 (176, 204) | 3.59 (3.41, 3.77) | 69 (44, 132) | 232 (135, 510) | 3.99 (3.78, 4.20) | 3 (2, 6) | 9 (5, 21) | 4.18 (3.97, 4.39) |
| Georgia | 272 (236, 317) | 326 (290, 369) | 1.14 (0.90, 1.37) | 2081 (1888, 2268) | 2053 (1873, 2244) | -0.28 (-0.45, -0.12) | 3258 (2417, 4012) | 4813 (4045, 5584) | 2.63 (1.66, 3.62) | 145 (100, 183) | 259 (215, 305) | 3.75 (2.58, 4.93) |
| Germany | 3316 (2835, 3960) | 3354 (2847, 4011) | -0.38 (-0.56, -0.19) | 52356 (47866, 56531) | 57640 (53550, 61636) | 0.25 (0.21, 0.30) | 51503 (45465, 59057) | 43439 (37942, 48228) | -0.68 (-0.88, -0.48) | 2383 (2060, 2814) | 2078 (1682, 2351) | -0.53 (-0.77, -0.27) |
| Ghana | 71 (54, 94) | 183 (138, 244) | 3.03 (2.99, 3.07) | 1651 (1517, 1792) | 4303 (4014, 4648) | 3.06 (3.01, 3.11) | 1900 (1196, 4035) | 4535 (2866, 8666) | 3.04 (2.82, 3.27) | 69 (39, 156) | 168 (98, 334) | 3.20 (2.92, 3.47) |
| Greece | 323 (264, 391) | 461 (386, 542) | 1.19 (0.91, 1.46) | 4849 (4487, 5195) | 7497 (6934, 8044) | 1.52 (1.45, 1.59) | 4994 (3576, 6084) | 6268 (5577, 6953) | 0.71 (0.45, 0.98) | 236 (162, 293) | 340 (281, 378) | 1.11 (0.74, 1.49) |
| Greenland | 1 (1, 2) | 2 (2, 3) | 2.29 (2.10, 2.49) | 15 (13, 23) | 27 (25, 30) | 1.74 (1.52, 1.96) | 62 (45, 76) | 49 (39, 63) | -1.33 (-1.62, -1.04) | 2 (2, 3) | 2 (2, 3) | -1.36 (-1.64, -1.07) |
| Grenada | 3 (2, 3) | 3 (2, 4) | 0.69 (0.53, 0.84) | 37 (34, 40) | 47 (44, 51) | 0.96 (0.86, 1.06) | 46 (35, 57) | 49 (43, 57) | 0.67 (0.34, 0.99) | 2 (1, 2) | 2 (2, 2) | 0.54 (0.20, 0.88) |
| Guam | 2 (2, 3) | 5 (5, 6) | 2.55 (2.39, 2.72) | 28 (26, 30) | 81 (76, 86) | 3.47 (3.42, 3.52) | 40 (31, 48) | 61 (50, 75) | 1.20 (0.98, 1.42) | 2 (1, 2) | 2 (2, 3) | 0.68 (0.40, 0.96) |
| Guatemala | 61 (47, 80) | 208 (166, 256) | 4.35 (4.25, 4.46) | 1274 (1180, 1362) | 4163 (3875, 4470) | 4.04 (4.00, 4.08) | 577 (480, 691) | 2387 (2053, 2783) | 4.76 (4.51, 5.00) | 19 (15, 23) | 89 (76, 105) | 5.23 (4.96, 5.50) |
| Guinea | 37 (28, 49) | 52 (40, 67) | 0.79 (0.60, 0.98) | 746 (678, 816) | 1106 (1023, 1189) | 0.91 (0.72, 1.10) | 898 (520, 1940) | 1339 (784, 3094) | 1.23 (1.09, 1.37) | 34 (18, 79) | 51 (28, 132) | 1.35 (1.14, 1.55) |
| Guinea-Bissau | 4 (3, 6) | 7 (5, 9) | 1.49 (1.42, 1.57) | 89 (81, 98) | 150 (140, 161) | 1.60 (1.53, 1.68) | 137 (72, 301) | 241 (139, 538) | 1.98 (1.93, 2.03) | 5 (2, 12) | 9 (5, 22) | 2.05 (1.98, 2.11) |
| Guyana | 12 (10, 15) | 17 (14, 20) | 0.92 (0.67, 1.17) | 151 (137, 167) | 254 (234, 274) | 1.78 (1.57, 1.98) | 206 (177, 240) | 284 (221, 360) | 1.56 (1.24, 1.88) | 8 (7, 9) | 11 (8, 14) | 1.49 (1.17, 1.80) |
| Haiti | 112 (87, 143) | 240 (194, 296) | 2.31 (2.22, 2.40) | 1391 (1235, 1554) | 2970 (2651, 3296) | 2.40 (2.28, 2.53) | 4195 (1301, 6851) | 7681 (2797, 13326) | 2.07 (1.99, 2.14) | 164 (49, 279) | 298 (103, 526) | 2.05 (1.97, 2.13) |
| Honduras | 55 (44, 68) | 226 (187, 278) | 4.82 (4.73, 4.91) | 912 (836, 993) | 2864 (2617, 3085) | 3.78 (3.71, 3.86) | 1301 (854, 2237) | 7717 (5207, 10490) | 6.30 (6.01, 6.59) | 52 (33, 94) | 330 (214, 452) | 6.47 (6.13, 6.80) |
| Hungary | 254 (218, 296) | 308 (272, 353) | 0.72 (0.68, 0.76) | 3173 (2896, 3495) | 2981 (2694, 3300) | -0.05 (-0.13, 0.03) | 4985 (4626, 5392) | 4070 (3486, 4597) | -0.40 (-0.68, -0.12) | 195 (180, 210) | 181 (155, 206) | -0.01 (-0.33, 0.32) |
| Iceland | 9 (7, 11) | 11 (9, 13) | 0.48 (0.31, 0.64) | 123 (113, 133) | 181 (167, 193) | 1.27 (1.16, 1.37) | 120 (107, 131) | 126 (109, 141) | 0.28 (0.02, 0.55) | 5 (5, 6) | 7 (5, 8) | 0.76 (0.42, 1.10) |
| India | 10098 (7947, 12815) | 18986 (15286, 23247) | 1.70 (1.54, 1.86) | 80406 (67110, 97851) | 179995 (152872, 215355) | 2.60 (2.51, 2.68) | 194147 (102299, 350468) | 398251 (302990, 527463) | 2.26 (2.10, 2.42) | 7180 (3662, 13791) | 15362 (11375, 21250) | 2.45 (2.32, 2.59) |
| Indonesia | 3028 (2417, 3805) | 6270 (5085, 7827) | 2.04 (1.92, 2.17) | 37302 (31555, 44396) | 78705 (67562, 92518) | 2.12 (1.95, 2.29) | 76215 (45495, 117940) | 145262 (96235, 226172) | 1.88 (1.80, 1.96) | 2970 (1672, 5248) | 5778 (3597, 10065) | 1.98 (1.86, 2.11) |
| Iran (Islamic Republic of) | 347 (270, 440) | 750 (609, 928) | 2.45 (2.37, 2.53) | 4564 (3922, 5243) | 11253 (9895, 12730) | 2.99 (2.87, 3.12) | 6376 (4118, 9415) | 9321 (7345, 11329) | 1.17 (0.98, 1.36) | 265 (159, 417) | 419 (291, 512) | 1.54 (1.38, 1.70) |
| Iraq | 161 (136, 192) | 218 (174, 272) | 0.54 (0.32, 0.76) | 1795 (1623, 1971) | 3700 (3368, 3981) | 2.37 (2.28, 2.46) | 2369 (1634, 4079) | 4269 (3058, 5786) | 1.45 (1.31, 1.60) | 95 (62, 177) | 167 (114, 233) | 1.31 (1.14, 1.48) |
| Ireland | 159 (133, 191) | 181 (154, 215) | 0.13 (-0.05, 0.31) | 2217 (2025, 2413) | 2628 (2430, 2819) | 0.46 (0.35, 0.57) | 2794 (2578, 2997) | 2052 (1762, 2308) | -0.82 (-1.00, -0.65) | 124 (114, 134) | 106 (86, 122) | -0.23 (-0.44, -0.03) |
| Israel | 87 (68, 109) | 175 (145, 210) | 2.22 (2.16, 2.28) | 1447 (1327, 1573) | 2980 (2761, 3218) | 2.38 (2.35, 2.42) | 918 (819, 1031) | 1433 (1246, 1611) | 1.48 (1.18, 1.79) | 34 (31, 39) | 63 (52, 71) | 2.13 (1.62, 2.64) |
| Italy | 1737 (1390, 2162) | 2192 (1884, 2512) | 0.74 (0.67, 0.80) | 21432 (18373, 24919) | 27979 (24425, 32534) | 0.91 (0.88, 0.95) | 24597 (22954, 26014) | 26870 (22883, 29578) | 0.70 (0.43, 0.97) | 1077 (976, 1137) | 1585 (1265, 1772) | 1.87 (1.47, 2.27) |
| Jamaica | 57 (45, 72) | 93 (77, 111) | 1.55 (1.39, 1.70) | 932 (855, 1017) | 1507 (1402, 1608) | 1.60 (1.52, 1.68) | 1020 (844, 1200) | 1390 (1107, 1715) | 1.29 (0.96, 1.62) | 43 (35, 51) | 61 (48, 77) | 1.46 (1.15, 1.78) |
| Japan | 11499 (9052, 14279) | 17754 (14939, 20994) | 1.31 (1.08, 1.54) | 148027 (127767, 174943) | 340212 (298451, 392229) | 2.82 (2.63, 3.00) | 157809 (145814, 168756) | 178635 (148207, 201737) | 0.11 (-0.08, 0.30) | 6518 (5868, 6884) | 8318 (6132, 9582) | 0.42 (0.21, 0.64) |
| Jordan | 15 (12, 18) | 51 (40, 66) | 3.48 (3.11, 3.85) | 228 (208, 249) | 969 (892, 1052) | 4.46 (4.14, 4.78) | 124 (87, 178) | 391 (296, 509) | 3.31 (3.11, 3.50) | 4 (3, 6) | 12 (8, 16) | 3.15 (2.97, 3.34) |
| Kazakhstan | 346 (291, 414) | 469 (405, 541) | 0.88 (0.61, 1.16) | 2961 (2623, 3270) | 4058 (3682, 4432) | 0.80 (0.48, 1.13) | 6853 (5435, 9067) | 9886 (8396, 11511) | 0.62 (0.34, 0.90) | 304 (237, 416) | 461 (395, 532) | 0.90 (0.61, 1.20) |
| Kenya | 119 (89, 153) | 287 (222, 368) | 2.79 (2.71, 2.86) | 1867 (1597, 2195) | 4970 (4303, 5827) | 3.12 (3.06, 3.19) | 1319 (756, 3360) | 3598 (2115, 8999) | 3.80 (3.58, 4.01) | 46 (23, 134) | 127 (62, 360) | 3.97 (3.70, 4.25) |
| Kiribati | 2 (1, 2) | 3 (3, 4) | 2.04 (1.91, 2.17) | 20 (18, 22) | 42 (39, 46) | 2.56 (2.36, 2.77) | 21 (16, 27) | 41 (30, 56) | 2.36 (2.21, 2.51) | 1 (1, 1) | 1 (1, 2) | 2.28 (2.18, 2.37) |
| Kuwait | 5 (4, 6) | 23 (17, 29) | 4.93 (4.53, 5.33) | 88 (79, 96) | 431 (395, 465) | 5.04 (4.69, 5.39) | 83 (69, 98) | 113 (91, 140) | 0.43 (-0.56, 1.44) | 3 (3, 4) | 2 (2, 3) | -1.76 (-2.96, -0.55) |
| Kyrgyzstan | 80 (67, 95) | 96 (79, 115) | 0.37 (0.02, 0.73) | 839 (745, 940) | 1076 (972, 1178) | 0.50 (0.04, 0.96) | 1516 (1303, 1767) | 2186 (1844, 2551) | 1.13 (0.83, 1.42) | 65 (55, 79) | 90 (75, 106) | 1.08 (0.75, 1.41) |
| Lao People's Democratic Republic | 65 (52, 81) | 103 (86, 125) | 1.09 (0.93, 1.26) | 701 (631, 779) | 1268 (1160, 1380) | 1.74 (1.57, 1.92) | 2299 (950, 3595) | 2254 (1440, 3467) | -0.43 (-0.58, -0.27) | 90 (34, 152) | 90 (53, 155) | -0.35 (-0.48, -0.23) |
| Latvia | 101 (85, 120) | 72 (61, 85) | -1.36 (-1.50, -1.23) | 925 (834, 1026) | 740 (653, 820) | -0.39 (-0.49, -0.29) | 1511 (1322, 1705) | 1196 (1051, 1359) | -1.48 (-1.99, -0.98) | 64 (55, 73) | 64 (56, 73) | -0.74 (-1.32, -0.14) |
| Lebanon | 40 (33, 50) | 77 (64, 94) | 1.79 (1.66, 1.92) | 544 (495, 594) | 1175 (1069, 1275) | 2.43 (2.38, 2.49) | 1169 (608, 1788) | 1019 (786, 1310) | -1.02 (-1.31, -0.74) | 51 (25, 81) | 51 (36, 67) | -0.55 (-0.83, -0.27) |
| Lesotho | 15 (12, 19) | 22 (18, 27) | 1.35 (1.08, 1.62) | 223 (203, 244) | 304 (280, 328) | 0.89 (0.74, 1.04) | 190 (109, 369) | 342 (208, 568) | 2.75 (2.16, 3.35) | 8 (4, 16) | 13 (8, 24) | 2.71 (2.08, 3.35) |
| Liberia | 11 (8, 14) | 18 (14, 23) | 1.23 (1.07, 1.40) | 228 (208, 248) | 371 (343, 400) | 1.38 (1.24, 1.53) | 269 (166, 541) | 470 (243, 1205) | 1.57 (1.37, 1.77) | 10 (6, 21) | 17 (8, 52) | 1.49 (1.32, 1.66) |
| Libya | 24 (19, 31) | 46 (37, 57) | 1.71 (1.42, 1.99) | 332 (299, 366) | 787 (718, 851) | 2.64 (2.47, 2.81) | 370 (223, 628) | 932 (526, 1497) | 3.10 (2.83, 3.36) | 15 (8, 28) | 38 (20, 62) | 3.29 (3.05, 3.52) |
| Lithuania | 100 (85, 118) | 99 (85, 118) | -0.03 (-0.11, 0.05) | 919 (826, 1026) | 1125 (973, 1278) | 0.99 (0.88, 1.10) | 1260 (1093, 1468) | 1613 (1390, 1813) | 0.41 (-0.28, 1.10) | 51 (45, 60) | 86 (75, 97) | 1.20 (0.41, 1.98) |
| Luxembourg | 11 (9, 14) | 15 (12, 18) | 0.55 (0.20, 0.90) | 138 (126, 150) | 199 (184, 214) | 1.01 (0.70, 1.33) | 181 (165, 200) | 164 (144, 184) | -0.34 (-0.62, -0.06) | 8 (8, 9) | 8 (7, 10) | 0.11 (-0.22, 0.44) |
| Madagascar | 95 (73, 123) | 205 (156, 263) | 2.43 (2.23, 2.63) | 1687 (1525, 1854) | 3527 (3245, 3822) | 2.39 (2.17, 2.61) | 1920 (1068, 4320) | 4032 (2185, 9163) | 2.39 (2.14, 2.64) | 69 (35, 170) | 139 (66, 338) | 2.24 (2.00, 2.49) |
| Malawi | 64 (48, 84) | 108 (82, 139) | 1.63 (1.42, 1.84) | 963 (871, 1061) | 1685 (1534, 1833) | 1.78 (1.59, 1.97) | 1091 (582, 2771) | 1847 (899, 5033) | 1.59 (1.47, 1.71) | 40 (19, 113) | 70 (29, 217) | 1.64 (1.53, 1.76) |
| Malaysia | 352 (292, 429) | 661 (546, 818) | 1.85 (1.71, 1.99) | 3555 (3241, 3887) | 9174 (8437, 9852) | 3.11 (3.05, 3.17) | 5451 (4158, 7255) | 10575 (7825, 13513) | 2.18 (2.05, 2.31) | 228 (167, 321) | 439 (312, 588) | 2.17 (2.04, 2.30) |
| Maldives | 3 (2, 3) | 6 (5, 8) | 2.30 (2.04, 2.57) | 29 (27, 32) | 75 (70, 81) | 2.69 (2.50, 2.87) | 56 (24, 79) | 71 (55, 90) | 0.11 (-0.18, 0.41) | 2 (1, 3) | 3 (2, 4) | 0.87 (0.61, 1.12) |
| Mali | 40 (30, 53) | 67 (51, 89) | 1.64 (1.47, 1.80) | 804 (733, 878) | 1482 (1368, 1596) | 2.11 (1.97, 2.25) | 1121 (632, 2373) | 1870 (1096, 3764) | 1.88 (1.67, 2.10) | 41 (21, 92) | 69 (37, 148) | 1.98 (1.74, 2.22) |
| Malta | 9 (7, 12) | 14 (11, 17) | 1.27 (1.17, 1.38) | 153 (140, 167) | 275 (252, 295) | 2.02 (1.98, 2.05) | 102 (90, 112) | 125 (107, 142) | 0.83 (0.55, 1.12) | 4 (3, 4) | 5 (4, 6) | 0.94 (0.57, 1.32) |
| Marshall Islands | 1 (1, 1) | 1 (1, 2) | 1.78 (1.52, 2.05) | 8 (7, 9) | 17 (15, 19) | 2.82 (2.45, 3.19) | 17 (10, 28) | 28 (15, 42) | 1.64 (1.43, 1.84) | 1 (0, 1) | 1 (1, 2) | 0.84 (0.63, 1.06) |
| Mauritania | 12 (9, 15) | 19 (14, 26) | 1.56 (1.34, 1.78) | 247 (226, 268) | 433 (397, 469) | 1.82 (1.63, 2.01) | 318 (195, 674) | 468 (278, 975) | 1.32 (1.04, 1.60) | 12 (7, 26) | 17 (9, 39) | 1.22 (0.96, 1.49) |
| Mauritius | 21 (17, 25) | 45 (37, 54) | 2.29 (2.19, 2.40) | 320 (288, 351) | 798 (715, 875) | 3.34 (3.21, 3.48) | 472 (425, 519) | 913 (818, 1006) | 1.31 (0.81, 1.82) | 19 (17, 21) | 39 (34, 43) | 1.46 (0.95, 1.98) |
| Mexico | 962 (750, 1214) | 3332 (2729, 4091) | 4.20 (4.15, 4.26) | 18289 (15862, 21136) | 53680 (46971, 62216) | 3.60 (3.53, 3.67) | 12081 (11433, 12753) | 46498 (40353, 53090) | 4.60 (4.40, 4.80) | 464 (446, 481) | 1977 (1687, 2261) | 5.01 (4.76, 5.26) |
| Micronesia (Federated States of) | 3 (2, 3) | 4 (3, 4) | 0.84 (0.62, 1.05) | 25 (22, 28) | 39 (35, 42) | 1.54 (1.29, 1.79) | 60 (34, 93) | 64 (38, 98) | 0.17 (-0.16, 0.51) | 3 (1, 4) | 3 (1, 4) | -0.27 (-0.57, 0.02) |
| Monaco | 2 (1, 2) | 1 (1, 2) | -0.73 (-0.85, -0.61) | 22 (20, 24) | 24 (22, 26) | 0.15 (0.11, 0.19) | 33 (22, 49) | 19 (14, 26) | -1.73 (-1.86, -1.61) | 2 (1, 3) | 1 (1, 1) | -1.92 (-2.07, -1.77) |
| Mongolia | 32 (27, 37) | 76 (64, 90) | 2.68 (2.49, 2.87) | 245 (216, 274) | 547 (495, 603) | 2.54 (2.10, 2.99) | 940 (596, 1713) | 2031 (1496, 2698) | 2.40 (2.13, 2.67) | 45 (28, 82) | 93 (67, 125) | 2.21 (1.96, 2.47) |
| Montenegro | 9 (7, 10) | 13 (11, 15) | 1.36 (1.18, 1.55) | 92 (83, 101) | 130 (117, 143) | 0.99 (0.91, 1.07) | 142 (112, 180) | 225 (155, 315) | 1.31 (0.94, 1.68) | 6 (5, 8) | 11 (7, 16) | 1.79 (1.44, 2.14) |
| Morocco | 255 (202, 317) | 512 (421, 631) | 2.10 (2.01, 2.18) | 3162 (2829, 3499) | 6684 (6108, 7234) | 2.35 (2.24, 2.45) | 5812 (2441, 14409) | 11013 (5306, 17334) | 2.22 (2.12, 2.32) | 245 (92, 647) | 468 (202, 775) | 2.32 (2.23, 2.42) |
| Mozambique | 106 (79, 142) | 195 (150, 255) | 1.98 (1.90, 2.06) | 1569 (1405, 1734) | 2990 (2744, 3248) | 2.09 (2.03, 2.14) | 1850 (980, 5270) | 3505 (1712, 8529) | 2.46 (2.23, 2.69) | 69 (32, 219) | 130 (55, 355) | 2.50 (2.27, 2.74) |
| Myanmar | 764 (623, 966) | 1155 (966, 1382) | 0.95 (0.80, 1.11) | 8180 (7361, 9102) | 13841 (12639, 15028) | 1.53 (1.36, 1.69) | 21723 (12026, 36692) | 23730 (16900, 37233) | -0.27 (-0.47, -0.08) | 850 (432, 1507) | 974 (668, 1718) | -0.13 (-0.32, 0.07) |
| Namibia | 12 (9, 15) | 21 (17, 26) | 1.55 (1.41, 1.69) | 178 (163, 193) | 332 (308, 357) | 1.83 (1.73, 1.93) | 140 (97, 242) | 240 (164, 419) | 1.33 (1.08, 1.58) | 5 (3, 9) | 9 (6, 17) | 1.42 (1.13, 1.72) |
| Nauru | 0 (0, 0) | 0 (0, 0) | 0.33 (0.26, 0.41) | 2 (2, 2) | 3 (2, 3) | 1.29 (1.26, 1.32) | 4 (3, 7) | 6 (4, 8) | 0.77 (0.44, 1.11) | 0 (0, 0) | 0 (0, 0) | 0.78 (0.40, 1.16) |
| Nepal | 215 (168, 274) | 507 (410, 630) | 2.81 (2.70, 2.92) | 2095 (1814, 2341) | 5111 (4558, 5710) | 3.04 (2.97, 3.12) | 5665 (2558, 13636) | 9034 (5676, 16456) | 1.62 (1.27, 1.97) | 214 (86, 562) | 355 (209, 698) | 1.76 (1.40, 2.12) |
| Netherlands | 547 (460, 658) | 647 (555, 768) | 0.67 (0.54, 0.80) | 8155 (7467, 8764) | 10709 (9939, 11482) | 0.91 (0.86, 0.96) | 8937 (8147, 9724) | 8292 (7344, 9143) | -0.44 (-0.60, -0.27) | 410 (363, 449) | 417 (353, 465) | -0.24 (-0.45, -0.03) |
| New Zealand | 116 (91, 145) | 154 (131, 181) | 0.68 (0.52, 0.85) | 1311 (1111, 1566) | 2007 (1734, 2331) | 1.22 (1.04, 1.41) | 2283 (2086, 2464) | 2415 (2116, 2686) | 0.01 (-0.20, 0.23) | 101 (92, 110) | 117 (99, 132) | 0.30 (0.03, 0.57) |
| Nicaragua | 50 (41, 62) | 141 (114, 173) | 3.23 (3.15, 3.30) | 798 (737, 857) | 2355 (2184, 2518) | 3.45 (3.40, 3.50) | 575 (460, 729) | 1514 (1153, 1907) | 3.47 (3.28, 3.67) | 22 (17, 29) | 56 (41, 74) | 3.50 (3.27, 3.73) |
| Niger | 27 (20, 36) | 84 (63, 111) | 3.69 (3.63, 3.75) | 559 (511, 612) | 1735 (1597, 1871) | 3.77 (3.71, 3.83) | 635 (351, 1256) | 1916 (1057, 4844) | 3.64 (3.56, 3.71) | 23 (11, 51) | 70 (34, 191) | 3.66 (3.57, 3.75) |
| Nigeria | 426 (331, 550) | 768 (586, 1030) | 1.60 (1.26, 1.94) | 8600 (7436, 9993) | 16614 (14498, 18911) | 2.04 (1.73, 2.35) | 8991 (5277, 18100) | 12643 (8260, 24734) | 0.62 (0.27, 0.98) | 365 (193, 796) | 439 (254, 948) | -0.01 (-0.36, 0.34) |
| Niue | 0 (0, 0) | 0 (0, 0) | -1.88 (-2.07, -1.69) | 1 (1, 1) | 1 (1, 1) | -0.72 (-0.83, -0.62) | 2 (2, 3) | 1 (1, 2) | -2.10 (-2.31, -1.88) | 0 (0, 0) | 0 (0, 0) | -2.56 (-2.84, -2.28) |
| North Macedonia | 58 (48, 68) | 123 (109, 139) | 2.70 (2.56, 2.84) | 508 (449, 565) | 829 (736, 923) | 1.51 (1.48, 1.54) | 1676 (1303, 2166) | 2610 (1889, 3437) | 1.42 (1.12, 1.72) | 69 (53, 92) | 118 (83, 158) | 1.76 (1.44, 2.09) |
| Northern Mariana Islands | 0 (0, 0) | 1 (1, 2) | 4.23 (4.07, 4.39) | 5 (4, 5) | 20 (18, 21) | 4.96 (4.76, 5.16) | 11 (8, 15) | 31 (24, 39) | 3.35 (3.12, 3.58) | 0 (0, 1) | 1 (1, 2) | 3.09 (2.89, 3.28) |
| Norway | 205 (169, 250) | 198 (164, 237) | -0.02 (-0.15, 0.10) | 2886 (2451, 3350) | 3427 (3001, 3931) | 0.58 (0.53, 0.64) | 3898 (3611, 4152) | 2241 (1968, 2474) | -1.63 (-1.82, -1.43) | 196 (175, 208) | 119 (99, 131) | -1.50 (-1.80, -1.21) |
| Oman | 15 (11, 25) | 54 (45, 63) | 4.17 (3.87, 4.48) | 167 (150, 188) | 436 (398, 474) | 2.95 (2.62, 3.27) | 190 (122, 307) | 245 (172, 326) | 0.72 (0.42, 1.02) | 7 (4, 13) | 9 (5, 12) | 0.45 (0.08, 0.82) |
| Pakistan | 1381 (1095, 1725) | 2480 (1986, 3060) | 1.57 (1.44, 1.70) | 12817 (10664, 15407) | 26690 (22648, 31836) | 2.33 (2.23, 2.43) | 25366 (13017, 45930) | 52958 (32070, 84299) | 1.94 (1.76, 2.11) | 995 (487, 1925) | 2024 (1173, 3347) | 1.84 (1.66, 2.03) |
| Palau | 0 (0, 1) | 1 (1, 1) | 1.27 (1.09, 1.46) | 5 (4, 5) | 11 (10, 12) | 2.50 (2.32, 2.68) | 6 (5, 9) | 11 (8, 14) | 1.64 (1.46, 1.82) | 0 (0, 0) | 0 (0, 1) | 1.47 (1.33, 1.60) |
| Palestine | 13 (11, 17) | 24 (20, 30) | 1.68 (1.52, 1.85) | 185 (168, 203) | 436 (400, 472) | 2.63 (2.53, 2.73) | 147 (100, 220) | 237 (190, 296) | 1.49 (1.37, 1.62) | 6 (4, 9) | 8 (6, 11) | 1.30 (1.10, 1.50) |
| Panama | 56 (47, 68) | 153 (127, 179) | 3.15 (3.07, 3.22) | 800 (744, 858) | 2357 (2206, 2500) | 3.53 (3.49, 3.56) | 704 (618, 805) | 1951 (1577, 2304) | 3.35 (3.10, 3.61) | 29 (26, 34) | 86 (67, 102) | 3.48 (3.23, 3.74) |
| Papua New Guinea | 52 (41, 64) | 113 (91, 134) | 2.39 (2.26, 2.53) | 515 (468, 566) | 1214 (1115, 1313) | 2.69 (2.47, 2.92) | 1802 (1069, 2812) | 3365 (2084, 5308) | 1.88 (1.70, 2.07) | 72 (41, 113) | 137 (82, 217) | 1.91 (1.80, 2.03) |
| Paraguay | 63 (52, 75) | 166 (138, 196) | 3.42 (3.34, 3.49) | 908 (822, 1002) | 2204 (2028, 2403) | 2.81 (2.77, 2.85) | 1336 (1015, 1816) | 3308 (2302, 4484) | 3.46 (3.26, 3.67) | 54 (42, 76) | 134 (93, 183) | 3.55 (3.32, 3.77) |
| Peru | 407 (337, 493) | 806 (666, 963) | 2.28 (2.09, 2.47) | 6653 (6152, 7201) | 14962 (13996, 15898) | 2.59 (2.53, 2.64) | 6592 (4934, 9014) | 13348 (9554, 18254) | 1.94 (1.52, 2.35) | 265 (191, 386) | 550 (379, 752) | 2.09 (1.65, 2.54) |
| Philippines | 464 (364, 596) | 1765 (1408, 2182) | 4.87 (4.65, 5.09) | 6951 (5991, 8087) | 26494 (22906, 30866) | 4.65 (4.56, 4.73) | 8875 (7517, 11215) | 32503 (25613, 39807) | 4.67 (4.53, 4.80) | 346 (291, 491) | 1236 (953, 1571) | 4.54 (4.41, 4.67) |
| Poland | 984 (802, 1210) | 994 (833, 1181) | -0.23 (-0.41, -0.06) | 11285 (9568, 13342) | 11385 (9766, 13347) | 0.10 (-0.09, 0.30) | 18213 (17293, 19155) | 16157 (14449, 17877) | -0.37 (-0.68, -0.05) | 709 (675, 740) | 730 (639, 807) | 0.01 (-0.31, 0.33) |
| Portugal | 357 (302, 430) | 457 (370, 521) | 0.64 (0.53, 0.75) | 4472 (4066, 4888) | 4604 (4193, 5015) | -0.13 (-0.22, -0.05) | 6737 (6245, 7224) | 6734 (5750, 7464) | 0.46 (0.22, 0.70) | 314 (290, 337) | 402 (326, 452) | 1.41 (1.12, 1.70) |
| Puerto Rico | 91 (74, 112) | 141 (114, 171) | 1.32 (1.14, 1.50) | 1724 (1605, 1842) | 2995 (2815, 3170) | 1.77 (1.65, 1.88) | 1110 (1004, 1241) | 1523 (1288, 1770) | 0.55 (0.22, 0.88) | 46 (42, 51) | 68 (55, 80) | 0.72 (0.39, 1.06) |
| Qatar | 1 (1, 1) | 9 (7, 11) | 7.68 (7.17, 8.20) | 20 (18, 22) | 159 (147, 170) | 7.10 (6.61, 7.59) | 29 (20, 44) | 104 (77, 140) | 4.16 (3.93, 4.39) | 1 (1, 2) | 4 (3, 5) | 3.88 (3.55, 4.20) |
| Republic of Korea | 2305 (1887, 2862) | 3767 (3118, 4471) | 1.43 (1.01, 1.85) | 22209 (19946, 24557) | 52765 (49514, 55805) | 2.95 (2.68, 3.21) | 46407 (31592, 64854) | 36606 (29539, 44680) | -0.89 (-1.12, -0.65) | 2013 (1320, 2979) | 1635 (1216, 2073) | -0.75 (-0.95, -0.54) |
| Republic of Moldova | 72 (58, 90) | 77 (63, 93) | -0.08 (-0.25, 0.09) | 693 (613, 783) | 813 (713, 916) | 0.48 (0.31, 0.66) | 976 (866, 1112) | 1117 (993, 1270) | -0.20 (-0.83, 0.44) | 39 (35, 45) | 48 (42, 55) | 0.04 (-0.70, 0.79) |
| Romania | 821 (709, 951) | 1361 (1236, 1515) | 1.70 (1.64, 1.77) | 5317 (4733, 5893) | 5415 (4797, 6031) | 0.06 (-0.03, 0.15) | 13761 (11749, 17053) | 16070 (13698, 18207) | 0.51 (0.00, 1.02) | 592 (498, 745) | 896 (761, 1020) | 1.50 (0.96, 2.05) |
| Russian Federation | 4599 (3700, 5683) | 7110 (6002, 8347) | 1.11 (0.71, 1.51) | 46429 (39046, 55786) | 58577 (50044, 68602) | 0.67 (0.55, 0.80) | 82403 (78718, 86351) | 123830 (111392, 135795) | 0.46 (-0.69, 1.62) | 3532 (3361, 3658) | 6436 (5736, 7051) | 1.14 (-0.30, 2.61) |
| Rwanda | 70 (53, 90) | 109 (85, 142) | 1.46 (1.01, 1.91) | 1096 (990, 1202) | 1759 (1620, 1907) | 1.84 (1.41, 2.28) | 1309 (656, 3255) | 1309 (676, 3084) | -0.40 (-0.96, 0.17) | 46 (21, 123) | 46 (19, 118) | -0.57 (-1.15, 0.01) |
| Saint Kitts and Nevis | 1 (1, 2) | 2 (1, 2) | 0.64 (0.12, 1.17) | 21 (20, 23) | 29 (27, 31) | 0.92 (0.44, 1.41) | 28 (22, 35) | 28 (23, 33) | -0.19 (-0.78, 0.41) | 1 (1, 2) | 1 (1, 1) | -0.59 (-1.09, -0.09) |
| Saint Lucia | 3 (3, 4) | 6 (5, 7) | 1.79 (1.53, 2.05) | 47 (43, 51) | 104 (97, 112) | 2.67 (2.53, 2.81) | 62 (53, 72) | 87 (72, 103) | 0.49 (0.04, 0.94) | 3 (2, 3) | 4 (3, 4) | 0.40 (-0.04, 0.85) |
| Saint Vincent and the Grenadines | 2 (2, 2) | 3 (2, 3) | 1.09 (0.78, 1.41) | 31 (29, 34) | 49 (45, 52) | 1.42 (1.23, 1.61) | 29 (25, 33) | 37 (33, 43) | 0.78 (0.45, 1.11) | 1 (1, 1) | 1 (1, 2) | 0.62 (0.32, 0.93) |
| Samoa | 4 (3, 5) | 6 (5, 7) | 0.98 (0.93, 1.02) | 41 (37, 45) | 69 (63, 75) | 1.64 (1.59, 1.68) | 71 (48, 100) | 103 (72, 140) | 1.08 (0.92, 1.24) | 3 (2, 5) | 4 (3, 6) | 1.02 (0.86, 1.19) |
| San Marino | 1 (0, 1) | 1 (1, 1) | 1.27 (1.19, 1.36) | 10 (9, 11) | 18 (16, 19) | 1.76 (1.66, 1.86) | 7 (5, 9) | 7 (5, 9) | 0.27 (0.09, 0.46) | 0 (0, 0) | 0 (0, 0) | -0.24 (-0.57, 0.08) |
| Sao Tome and Principe | 1 (1, 1) | 1 (1, 2) | 0.88 (0.64, 1.12) | 19 (17, 21) | 28 (26, 30) | 1.12 (0.90, 1.35) | 16 (10, 33) | 26 (17, 55) | 1.18 (1.02, 1.34) | 1 (0, 1) | 1 (1, 2) | 1.06 (0.95, 1.17) |
| Saudi Arabia | 52 (42, 63) | 106 (81, 139) | 1.80 (1.57, 2.04) | 690 (620, 761) | 1975 (1803, 2140) | 3.04 (2.85, 3.23) | 857 (569, 1421) | 1896 (1262, 2872) | 2.38 (2.24, 2.52) | 34 (22, 59) | 63 (39, 100) | 1.88 (1.71, 2.04) |
| Senegal | 31 (23, 41) | 70 (54, 94) | 2.67 (2.62, 2.73) | 666 (609, 728) | 1576 (1461, 1704) | 2.83 (2.80, 2.86) | 749 (489, 1449) | 1782 (1109, 3861) | 3.02 (2.89, 3.15) | 28 (17, 59) | 67 (40, 162) | 3.12 (2.98, 3.27) |
| Serbia | 371 (319, 430) | 629 (568, 695) | 1.65 (1.46, 1.84) | 3108 (2776, 3474) | 2808 (2503, 3159) | -0.57 (-0.64, -0.49) | 10526 (7678, 13838) | 8461 (6453, 11163) | -1.42 (-1.77, -1.06) | 431 (320, 570) | 411 (312, 538) | -0.76 (-1.09, -0.43) |
| Seychelles | 1 (1, 2) | 2 (2, 2) | 0.78 (0.52, 1.03) | 19 (17, 21) | 31 (28, 34) | 1.58 (1.45, 1.71) | 22 (15, 31) | 24 (15, 36) | 0.56 (0.33, 0.79) | 1 (1, 2) | 1 (0, 1) | 0.22 (-0.01, 0.45) |
| Sierra Leone | 19 (14, 26) | 33 (25, 43) | 1.65 (1.53, 1.78) | 418 (381, 455) | 743 (689, 798) | 1.78 (1.64, 1.93) | 444 (260, 1012) | 878 (507, 2024) | 2.49 (2.39, 2.60) | 17 (9, 43) | 33 (17, 84) | 2.42 (2.31, 2.54) |
| Singapore | 101 (81, 126) | 228 (177, 296) | 2.29 (2.09, 2.49) | 1465 (1360, 1568) | 4254 (3972, 4531) | 3.55 (3.37, 3.74) | 1311 (1201, 1428) | 1793 (1552, 2076) | 0.78 (0.66, 0.90) | 46 (42, 50) | 54 (46, 61) | 0.12 (-0.04, 0.27) |
| Slovakia | 148 (128, 174) | 133 (114, 156) | -0.70 (-0.83, -0.57) | 1392 (1275, 1515) | 1571 (1431, 1719) | 0.63 (0.55, 0.72) | 2396 (1824, 2982) | 2081 (1599, 2687) | -0.23 (-0.35, -0.10) | 99 (74, 125) | 91 (66, 122) | -0.05 (-0.18, 0.09) |
| Slovenia | 48 (41, 57) | 59 (50, 70) | 0.19 (0.02, 0.37) | 559 (506, 614) | 653 (598, 714) | 0.60 (0.55, 0.66) | 890 (802, 976) | 687 (574, 786) | -0.81 (-1.06, -0.56) | 37 (33, 40) | 35 (29, 41) | 0.02 (-0.29, 0.32) |
| Solomon Islands | 6 (5, 9) | 21 (18, 25) | 3.38 (3.16, 3.61) | 68 (59, 77) | 188 (168, 208) | 3.09 (3.01, 3.16) | 136 (77, 217) | 299 (196, 470) | 2.39 (2.23, 2.56) | 5 (3, 9) | 12 (7, 20) | 2.62 (2.49, 2.74) |
| Somalia | 40 (29, 53) | 112 (83, 148) | 3.59 (3.49, 3.70) | 646 (581, 714) | 1830 (1642, 2001) | 3.64 (3.55, 3.73) | 800 (328, 2688) | 1860 (667, 7338) | 2.99 (2.90, 3.08) | 28 (10, 107) | 64 (17, 301) | 2.98 (2.88, 3.09) |
| South Africa | 293 (227, 373) | 612 (484, 777) | 2.14 (1.92, 2.36) | 5351 (4603, 6263) | 10321 (9009, 11995) | 1.95 (1.86, 2.04) | 2547 (2073, 3090) | 7125 (5904, 8486) | 3.38 (3.03, 3.72) | 84 (64, 107) | 263 (207, 315) | 3.77 (3.30, 4.24) |
| South Sudan | 30 (23, 38) | 44 (34, 58) | 1.15 (0.87, 1.44) | 491 (450, 538) | 742 (680, 800) | 1.31 (1.11, 1.52) | 468 (276, 1004) | 708 (394, 1549) | 1.13 (0.59, 1.68) | 18 (9, 42) | 25 (12, 60) | 0.80 (0.22, 1.38) |
| Spain | 1053 (852, 1276) | 1776 (1544, 2047) | 1.78 (1.67, 1.88) | 16094 (14752, 17448) | 25757 (24009, 27535) | 1.55 (1.52, 1.57) | 12751 (11467, 13924) | 20923 (17796, 23514) | 1.97 (1.59, 2.35) | 559 (496, 610) | 1200 (948, 1379) | 3.04 (2.49, 3.59) |
| Sri Lanka | 382 (312, 459) | 875 (727, 1045) | 2.52 (2.38, 2.67) | 4183 (3835, 4540) | 10347 (9583, 11060) | 2.99 (2.98, 3.01) | 3053 (2261, 3971) | 6247 (4601, 8158) | 2.59 (2.46, 2.72) | 113 (76, 157) | 232 (147, 333) | 2.74 (2.54, 2.94) |
| Sudan | 148 (117, 183) | 210 (171, 256) | 0.63 (0.44, 0.81) | 1630 (1443, 1824) | 2526 (2299, 2759) | 1.09 (0.92, 1.26) | 3911 (1417, 9569) | 4818 (2035, 8626) | 0.31 (0.14, 0.47) | 165 (54, 459) | 205 (81, 403) | 0.34 (0.20, 0.48) |
| Suriname | 7 (6, 9) | 16 (13, 20) | 2.71 (2.61, 2.80) | 106 (97, 114) | 259 (240, 280) | 2.98 (2.87, 3.10) | 123 (97, 155) | 244 (180, 320) | 2.31 (2.08, 2.54) | 5 (4, 6) | 10 (7, 13) | 2.37 (2.15, 2.60) |
| Sweden | 393 (320, 475) | 399 (330, 478) | 0.02 (-0.07, 0.11) | 5301 (4519, 6252) | 5845 (5047, 6751) | 0.28 (0.23, 0.33) | 6950 (6314, 7478) | 4571 (3931, 5207) | -1.28 (-1.57, -0.98) | 346 (307, 375) | 239 (194, 276) | -1.11 (-1.49, -0.74) |
| Switzerland | 247 (212, 288) | 295 (249, 355) | 0.62 (0.24, 1.00) | 2688 (2450, 2914) | 4181 (3875, 4445) | 1.41 (1.24, 1.59) | 3494 (3079, 3931) | 3253 (2758, 3657) | 0.00 (-0.13, 0.14) | 176 (151, 202) | 180 (144, 207) | 0.40 (0.27, 0.54) |
| Syrian Arab Republic | 72 (56, 93) | 130 (103, 163) | 1.57 (1.36, 1.78) | 1136 (1037, 1238) | 2498 (2294, 2693) | 2.52 (2.41, 2.63) | 1342 (884, 2595) | 2303 (1586, 3255) | 1.21 (0.79, 1.64) | 54 (34, 119) | 89 (57, 133) | 1.11 (0.64, 1.57) |
| Taiwan, China | 368 (301, 460) | 986 (820, 1201) | 3.17 (3.13, 3.21) | 4517 (4058, 4961) | 12413 (11136, 13704) | 3.38 (3.28, 3.49) | 5881 (5378, 6387) | 9875 (8631, 10980) | 1.15 (0.87, 1.44) | 226 (208, 245) | 408 (345, 458) | 1.13 (0.78, 1.49) |
| Tajikistan | 63 (52, 75) | 145 (123, 175) | 2.75 (2.48, 3.02) | 612 (548, 681) | 1218 (1100, 1334) | 1.94 (1.26, 2.63) | 1086 (766, 1577) | 1825 (1284, 2536) | 1.06 (0.84, 1.28) | 48 (32, 80) | 72 (49, 101) | 0.65 (0.37, 0.94) |
| Thailand | 1869 (1566, 2235) | 4001 (3422, 4797) | 2.09 (1.93, 2.25) | 17209 (15791, 18576) | 42381 (39064, 45749) | 2.83 (2.76, 2.90) | 39993 (28311, 56284) | 68575 (52191, 88153) | 1.21 (1.00, 1.41) | 1905 (1269, 2754) | 3514 (2487, 4704) | 1.46 (1.23, 1.68) |
| Timor-Leste | 6 (5, 8) | 19 (16, 23) | 3.90 (3.73, 4.08) | 69 (63, 77) | 227 (206, 249) | 4.36 (4.13, 4.59) | 188 (96, 315) | 487 (278, 832) | 3.62 (3.40, 3.85) | 7 (4, 13) | 21 (11, 38) | 3.94 (3.70, 4.17) |
| Togo | 13 (10, 17) | 39 (29, 52) | 3.63 (3.57, 3.68) | 260 (234, 282) | 822 (759, 883) | 3.82 (3.79, 3.84) | 313 (181, 688) | 979 (561, 2486) | 3.91 (3.82, 3.99) | 11 (6, 27) | 36 (19, 97) | 3.97 (3.86, 4.07) |
| Tokelau | 0 (0, 0) | 0 (0, 0) | -1.44 (-1.59, -1.29) | 1 (1, 1) | 1 (1, 1) | -0.35 (-0.39, -0.30) | 2 (1, 3) | 1 (1, 1) | -1.96 (-2.04, -1.89) | 0 (0, 0) | 0 (0, 0) | -1.87 (-1.93, -1.80) |
| Tonga | 2 (2, 2) | 2 (2, 3) | 0.46 (0.36, 0.56) | 21 (19, 23) | 26 (24, 28) | 0.59 (0.51, 0.67) | 27 (19, 38) | 31 (22, 45) | 0.53 (0.46, 0.60) | 1 (1, 2) | 1 (1, 2) | 1.11 (1.00, 1.22) |
| Trinidad and Tobago | 25 (21, 30) | 50 (42, 61) | 2.30 (2.18, 2.43) | 363 (330, 395) | 763 (706, 821) | 2.47 (2.36, 2.58) | 468 (420, 517) | 746 (596, 927) | 1.25 (1.05, 1.45) | 19 (17, 21) | 32 (25, 40) | 1.35 (1.13, 1.58) |
| Tunisia | 84 (67, 107) | 178 (144, 213) | 2.08 (1.95, 2.21) | 1124 (1016, 1231) | 2656 (2424, 2866) | 2.66 (2.56, 2.76) | 1390 (780, 3099) | 2562 (1546, 4384) | 1.87 (1.79, 1.95) | 57 (29, 130) | 115 (60, 207) | 2.22 (2.13, 2.30) |
| Turkmenistan | 45 (38, 54) | 107 (90, 126) | 3.20 (2.87, 3.53) | 426 (382, 472) | 997 (907, 1089) | 2.79 (2.40, 3.18) | 893 (693, 1151) | 2524 (1579, 3418) | 3.36 (2.97, 3.75) | 35 (27, 46) | 101 (63, 141) | 3.44 (3.05, 3.83) |
| Tuvalu | 0 (0, 0) | 0 (0, 1) | 0.40 (0.31, 0.49) | 4 (3, 4) | 5 (5, 6) | 0.94 (0.82, 1.06) | 10 (5, 16) | 9 (6, 13) | -0.39 (-0.58, -0.21) | 0 (0, 1) | 0 (0, 1) | -0.32 (-0.47, -0.17) |
| Türkiye | 858 (693, 1051) | 1548 (1294, 1876) | 1.70 (1.61, 1.79) | 11195 (10275, 12273) | 22528 (20907, 24291) | 2.09 (2.03, 2.16) | 21806 (13269, 43011) | 28514 (21848, 37379) | 0.69 (0.37, 1.02) | 934 (536, 1976) | 1329 (978, 1758) | 1.30 (0.92, 1.69) |
| Uganda | 103 (77, 134) | 224 (170, 290) | 2.33 (2.20, 2.47) | 1555 (1407, 1728) | 3452 (3155, 3737) | 2.51 (2.37, 2.64) | 1248 (651, 3698) | 2221 (1303, 4936) | 1.32 (1.09, 1.56) | 45 (19, 156) | 77 (37, 202) | 1.12 (0.86, 1.38) |
| Ukraine | 2404 (1969, 2961) | 1917 (1620, 2268) | -0.90 (-1.05, -0.75) | 16607 (14044, 19851) | 16171 (13765, 18876) | -0.18 (-0.29, -0.07) | 47376 (39517, 55985) | 31956 (23033, 43666) | -1.67 (-1.83, -1.51) | 2509 (2062, 2944) | 1742 (1246, 2385) | -1.52 (-1.66, -1.37) |
| United Arab Emirates | 5 (4, 6) | 17 (12, 22) | 3.62 (3.20, 4.04) | 60 (54, 66) | 392 (362, 426) | 6.04 (5.39, 6.70) | 193 (113, 341) | 584 (404, 811) | 4.29 (3.95, 4.64) | 8 (5, 16) | 23 (15, 33) | 4.37 (3.93, 4.81) |
| United Kingdom | 2737 (2258, 3316) | 2860 (2453, 3358) | 0.05 (-0.06, 0.17) | 33232 (28941, 38500) | 35793 (31568, 40620) | 0.19 (0.06, 0.32) | 53701 (51269, 55892) | 39123 (35537, 41724) | -0.98 (-1.13, -0.83) | 2472 (2304, 2554) | 2114 (1818, 2271) | -0.28 (-0.48, -0.09) |
| United Republic of Tanzania | 182 (136, 234) | 384 (300, 494) | 2.26 (2.20, 2.33) | 2811 (2555, 3107) | 5934 (5455, 6399) | 2.28 (2.22, 2.35) | 2275 (1199, 5465) | 4346 (2444, 9153) | 2.02 (1.94, 2.09) | 79 (35, 215) | 156 (74, 366) | 2.24 (2.13, 2.35) |
| United States Virgin Islands | 3 (2, 3) | 5 (4, 6) | 1.82 (1.64, 1.99) | 42 (39, 46) | 83 (77, 89) | 2.30 (2.07, 2.53) | 44 (32, 59) | 41 (31, 56) | 0.00 (-0.28, 0.28) | 2 (1, 2) | 2 (1, 2) | -0.07 (-0.37, 0.23) |
| United States of America | 6756 (5407, 8353) | 11355 (9457, 13597) | 1.70 (1.41, 1.98) | 100679 (87292, 119817) | 195589 (173932, 219831) | 2.20 (2.09, 2.31) | 123025 (114986, 130153) | 191304 (174000, 205069) | 1.51 (1.40, 1.63) | 5288 (4790, 5571) | 9015 (7787, 9698) | 1.83 (1.65, 2.02) |
| Uruguay | 187 (157, 223) | 239 (204, 278) | 0.79 (0.69, 0.88) | 2207 (1941, 2495) | 2668 (2287, 3165) | 1.07 (0.86, 1.28) | 3767 (3369, 4226) | 3752 (3381, 4080) | -0.07 (-0.22, 0.08) | 160 (140, 179) | 185 (161, 204) | 0.42 (0.25, 0.58) |
| Uzbekistan | 173 (136, 223) | 387 (330, 464) | 2.84 (2.40, 3.28) | 1900 (1687, 2137) | 4242 (3867, 4594) | 2.38 (1.98, 2.78) | 1818 (1435, 2270) | 8338 (7085, 9888) | 4.34 (3.15, 5.54) | 67 (48, 93) | 357 (298, 430) | 4.70 (3.32, 6.09) |
| Vanuatu | 2 (2, 3) | 6 (5, 8) | 3.25 (3.16, 3.34) | 25 (22, 28) | 79 (72, 86) | 3.82 (3.74, 3.90) | 48 (24, 73) | 116 (62, 176) | 2.71 (2.51, 2.90) | 2 (1, 3) | 5 (2, 7) | 2.61 (2.44, 2.77) |
| Venezuela (Bolivarian Republic of) | 320 (263, 384) | 981 (821, 1189) | 3.81 (3.71, 3.90) | 5842 (5422, 6248) | 18148 (17072, 19317) | 3.89 (3.82, 3.96) | 4535 (4056, 5066) | 16225 (12058, 20810) | 4.12 (3.99, 4.26) | 170 (152, 193) | 663 (483, 863) | 4.43 (4.29, 4.57) |
| Viet Nam | 1443 (1171, 1779) | 2664 (2199, 3205) | 1.76 (1.57, 1.95) | 15652 (14005, 17239) | 32676 (30166, 35166) | 2.26 (2.00, 2.52) | 28714 (18368, 53344) | 43136 (28558, 78006) | 1.48 (1.31, 1.66) | 1330 (800, 2689) | 1942 (1108, 4076) | 1.45 (1.34, 1.56) |
| Yemen | 93 (74, 117) | 219 (180, 267) | 2.68 (2.54, 2.82) | 1021 (903, 1138) | 2477 (2217, 2724) | 2.97 (2.84, 3.09) | 2410 (967, 5605) | 5230 (2308, 11483) | 2.30 (2.08, 2.52) | 100 (37, 258) | 223 (94, 510) | 2.37 (2.15, 2.59) |
| Zambia | 45 (34, 58) | 102 (80, 132) | 2.61 (2.43, 2.79) | 685 (623, 748) | 1605 (1476, 1726) | 2.81 (2.65, 2.97) | 795 (444, 1801) | 1733 (1002, 3779) | 2.30 (2.06, 2.54) | 29 (14, 70) | 64 (33, 150) | 2.41 (2.17, 2.64) |
| Zimbabwe | 60 (47, 77) | 135 (104, 170) | 2.86 (2.74, 2.97) | 939 (854, 1027) | 2257 (2064, 2437) | 2.70 (2.59, 2.81) | 1167 (871, 1812) | 3453 (2180, 5313) | 4.51 (3.83, 5.19) | 48 (35, 78) | 133 (80, 214) | 4.23 (3.51, 4.96) |

Note. Abbreviations: CI, confidence interval; EAPC, estimated annual percentage change; UI, uncertainty interval.
