# Supplementary material for: Global burden and trends of subarachnoid hemorrhage in women aged 55 years and older: A repeated cross-sectional analysis of the GBD 2021 study
Source: Medicine (Baltimore). 2026 Nov 6;105(45):e51457. doi: 10.1097/MD.0000000000051457 (PMC13747508; doi:10.1097/MD.0000000000051457)
Supplement: Supplementary file 3 [file medi-105-e51457-s004.docx]

Table S3. Age-standardized rates of incidence, prevalence, DALYs and deaths (95% UI) and EAPC (95% CI) of SAH-related indicators among postmenopausal women across GBD countries, 1990–2021

| **Location** | **ASIR (95% UI)** | | | **ASPR (95% UI)** | | | **ASDR (95% UI)** | | | **ASMR (95% UI)** | | |
| --- | --- | --- | --- | --- | --- | --- | --- | --- | --- | --- | --- | --- |
|  | **1990** | **2021** | **EAPC** | **1990** | **2021** | **EAPC** | **1990** | **2021** | **EAPC** | **1990** | **2021** | **EAPC** |
| Afghanistan | 23.55 (15.41, 34.11) | 22.74 (16.22, 30.84) | -0.39 (-0.50, -0.27) | 259.21 (214.78, 306.37) | 217.13 (185.98, 251.13) | -0.67 (-0.71, -0.62) | 648.66 (174.96, 1602.01) | 512.17 (150.22, 1053.01) | -1.00 (-1.19, -0.81) | 27.72 (6.23, 75.06) | 22.77 (5.88, 49.89) | -0.84 (-1.03, -0.64) |
| Albania | 18.82 (13.66, 25.12) | 19.16 (14.29, 25.04) | 0.12 (0.00, 0.24) | 195.51 (174.38, 219.44) | 174.68 (155.02, 195.00) | -0.40 (-0.42, -0.38) | 335.10 (228.45, 491.49) | 234.18 (135.31, 377.57) | -0.83 (-1.09, -0.57) | 15.16 (9.76, 22.84) | 11.32 (5.74, 19.31) | -0.51 (-0.83, -0.19) |
| Algeria | 22.47 (15.46, 31.41) | 15.32 (10.76, 21.01) | -1.62 (-1.76, -1.48) | 291.99 (255.35, 329.96) | 228.33 (204.79, 252.08) | -0.88 (-0.93, -0.83) | 379.78 (169.97, 806.08) | 249.40 (129.96, 438.00) | -1.16 (-1.26, -1.05) | 18.66 (7.16, 41.19) | 12.32 (5.24, 23.10) | -0.82 (-1.01, -0.63) |
| American Samoa | 37.32 (26.63, 50.68) | 34.34 (25.49, 45.40) | -0.27 (-0.30, -0.24) | 420.84 (375.50, 466.66) | 410.22 (371.72, 449.64) | -0.10 (-0.11, -0.09) | 701.52 (446.05, 1072.95) | 621.03 (386.86, 945.83) | -0.44 (-0.57, -0.31) | 32.75 (19.68, 51.74) | 28.47 (16.72, 44.68) | -0.48 (-0.62, -0.33) |
| Andorra | 21.31 (14.48, 29.90) | 19.88 (13.83, 26.89) | -0.24 (-0.32, -0.16) | 342.88 (305.66, 381.77) | 314.67 (283.53, 347.08) | -0.31 (-0.35, -0.27) | 416.05 (227.90, 691.14) | 253.69 (155.97, 387.75) | -1.38 (-1.53, -1.24) | 16.50 (8.27, 28.99) | 10.42 (5.59, 16.85) | -1.20 (-1.37, -1.03) |
| Angola | 19.81 (13.02, 28.62) | 15.81 (10.42, 22.53) | -0.91 (-1.00, -0.81) | 327.48 (292.36, 364.98) | 285.39 (257.85, 315.08) | -0.48 (-0.51, -0.45) | 328.34 (160.46, 751.00) | 263.95 (147.57, 538.73) | -1.01 (-1.13, -0.89) | 12.43 (5.14, 31.60) | 10.05 (4.84, 22.48) | -1.04 (-1.18, -0.90) |
| Antigua and Barbuda | 33.32 (23.94, 44.87) | 28.57 (20.56, 37.88) | -0.63 (-0.71, -0.56) | 526.39 (478.55, 573.42) | 464.10 (425.13, 502.48) | -0.47 (-0.50, -0.44) | 544.87 (432.94, 677.05) | 400.44 (329.29, 483.26) | -1.19 (-1.43, -0.96) | 22.66 (17.65, 28.57) | 16.43 (13.30, 20.03) | -1.27 (-1.53, -1.01) |
| Argentina | 49.02 (35.59, 65.53) | 30.22 (22.53, 39.90) | -1.82 (-1.96, -1.69) | 516.35 (433.14, 606.40) | 406.21 (332.86, 506.26) | -0.82 (-0.88, -0.75) | 999.26 (807.35, 1232.16) | 408.92 (332.72, 497.71) | -2.67 (-2.95, -2.38) | 40.70 (32.12, 50.77) | 15.83 (12.55, 19.48) | -2.76 (-3.12, -2.40) |
| Armenia | 23.96 (17.59, 31.66) | 13.62 (9.88, 18.31) | -2.25 (-2.49, -2.00) | 220.92 (195.84, 249.27) | 162.25 (145.83, 179.44) | -1.13 (-1.18, -1.08) | 437.58 (362.75, 520.25) | 187.95 (159.74, 218.82) | -2.84 (-3.33, -2.36) | 19.07 (15.64, 23.05) | 8.26 (7.07, 9.43) | -2.72 (-3.28, -2.16) |
| Australia | 25.41 (19.10, 33.22) | 19.14 (13.47, 26.43) | -0.90 (-1.02, -0.77) | 343.58 (313.17, 375.55) | 318.87 (293.70, 344.98) | -0.16 (-0.24, -0.09) | 473.14 (391.45, 562.63) | 239.79 (194.53, 287.04) | -2.22 (-2.34, -2.10) | 19.83 (16.19, 23.76) | 10.15 (7.92, 12.23) | -2.20 (-2.33, -2.07) |
| Austria | 38.76 (29.08, 50.32) | 27.47 (19.73, 36.99) | -0.50 (-0.81, -0.19) | 672.15 (581.19, 737.88) | 618.62 (563.10, 672.63) | -0.23 (-0.42, -0.04) | 388.72 (324.90, 457.11) | 275.75 (223.42, 333.16) | -0.99 (-1.13, -0.85) | 14.17 (11.51, 16.90) | 10.00 (7.74, 12.31) | -1.01 (-1.19, -0.84) |
| Azerbaijan | 16.36 (10.03, 24.97) | 13.36 (9.21, 18.49) | -0.34 (-0.50, -0.17) | 188.35 (159.81, 220.46) | 172.28 (149.63, 195.59) | -0.25 (-0.30, -0.20) | 159.61 (107.59, 241.17) | 132.33 (84.15, 200.61) | -0.77 (-1.07, -0.46) | 5.48 (3.27, 9.23) | 4.82 (2.56, 7.97) | -0.42 (-0.77, -0.07) |
| Bahamas | 26.26 (18.58, 36.18) | 22.06 (15.50, 30.15) | -0.65 (-0.70, -0.59) | 431.51 (392.22, 472.01) | 398.94 (364.88, 433.91) | -0.27 (-0.29, -0.25) | 398.93 (312.62, 517.31) | 303.35 (230.90, 392.64) | -0.90 (-1.01, -0.79) | 15.56 (11.90, 20.49) | 11.67 (8.73, 15.35) | -0.92 (-1.07, -0.78) |
| Bahrain | 14.85 (10.06, 20.97) | 9.81 (7.02, 13.19) | -1.49 (-1.59, -1.40) | 189.23 (164.72, 217.20) | 154.08 (137.63, 172.02) | -0.73 (-0.76, -0.70) | 505.32 (315.97, 781.91) | 202.83 (131.28, 302.48) | -4.01 (-4.32, -3.69) | 23.36 (14.17, 37.13) | 9.53 (5.78, 14.74) | -3.92 (-4.25, -3.59) |
| Bangladesh | 33.25 (22.14, 47.31) | 31.13 (22.28, 42.79) | -0.30 (-0.45, -0.14) | 349.16 (295.47, 406.18) | 304.11 (264.17, 346.29) | -0.36 (-0.43, -0.30) | 980.63 (410.77, 2265.99) | 653.62 (356.60, 1188.52) | -1.12 (-1.29, -0.94) | 42.51 (16.37, 106.36) | 29.06 (14.95, 55.77) | -1.07 (-1.31, -0.83) |
| Barbados | 30.18 (21.40, 40.92) | 25.09 (17.96, 34.02) | -0.54 (-0.66, -0.43) | 476.20 (433.86, 517.27) | 440.33 (403.14, 477.00) | -0.28 (-0.32, -0.25) | 477.61 (388.80, 584.37) | 439.68 (331.56, 569.96) | -0.05 (-0.29, 0.19) | 19.89 (15.78, 24.65) | 18.35 (13.63, 23.94) | -0.07 (-0.31, 0.18) |
| Belarus | 21.44 (15.25, 29.18) | 17.31 (12.74, 22.94) | -0.81 (-0.90, -0.71) | 198.60 (173.01, 226.14) | 186.53 (167.00, 206.17) | -0.22 (-0.26, -0.17) | 343.17 (249.58, 470.13) | 275.11 (212.95, 347.10) | -1.32 (-1.64, -0.99) | 14.16 (9.94, 20.10) | 11.92 (9.18, 15.16) | -1.14 (-1.46, -0.82) |
| Belgium | 18.42 (13.00, 25.46) | 16.62 (11.84, 22.75) | -0.61 (-0.96, -0.26) | 263.69 (237.89, 291.89) | 247.89 (224.97, 273.10) | -0.34 (-0.47, -0.20) | 272.17 (223.24, 326.19) | 216.93 (173.79, 262.07) | -0.54 (-0.83, -0.25) | 11.21 (9.05, 13.56) | 9.26 (7.13, 11.35) | -0.39 (-0.77, -0.02) |
| Belize | 26.69 (18.77, 36.04) | 21.89 (15.74, 29.36) | -0.89 (-0.99, -0.79) | 439.70 (399.43, 481.89) | 390.70 (359.42, 423.05) | -0.45 (-0.48, -0.41) | 355.31 (269.51, 460.30) | 291.12 (228.79, 360.03) | -0.93 (-1.24, -0.63) | 13.57 (10.14, 17.87) | 11.41 (8.83, 14.31) | -0.91 (-1.23, -0.58) |
| Benin | 13.64 (8.80, 20.33) | 11.63 (7.51, 17.26) | -0.66 (-0.71, -0.61) | 263.24 (236.39, 291.33) | 239.18 (217.01, 262.32) | -0.39 (-0.42, -0.36) | 360.87 (179.45, 814.11) | 270.12 (141.30, 589.57) | -0.89 (-0.99, -0.79) | 14.80 (6.56, 36.71) | 10.94 (5.07, 26.14) | -0.90 (-1.00, -0.80) |
| Bermuda | 28.35 (19.61, 39.19) | 18.56 (12.35, 26.32) | -1.77 (-1.91, -1.63) | 538.80 (495.64, 583.38) | 438.16 (406.53, 473.49) | -0.73 (-0.78, -0.69) | 376.93 (292.61, 477.86) | 172.15 (135.20, 221.87) | -3.06 (-3.32, -2.79) | 14.61 (10.92, 19.11) | 6.19 (4.60, 8.23) | -3.41 (-3.72, -3.09) |
| Bhutan | 29.76 (20.12, 41.63) | 20.65 (14.47, 28.01) | -1.47 (-1.57, -1.36) | 278.22 (237.06, 322.93) | 222.88 (194.35, 251.88) | -0.72 (-0.74, -0.70) | 687.28 (277.29, 1378.45) | 402.32 (225.70, 702.19) | -1.92 (-2.04, -1.81) | 27.24 (9.95, 58.01) | 16.26 (8.50, 29.41) | -1.84 (-1.96, -1.73) |
| Bolivia (Plurinational State of) | 57.50 (40.28, 77.68) | 46.52 (34.01, 62.84) | -0.85 (-0.91, -0.79) | 767.51 (678.85, 859.85) | 612.85 (549.02, 678.66) | -0.84 (-0.89, -0.79) | 1691.96 (849.42, 2888.70) | 1164.97 (636.60, 1925.86) | -1.25 (-1.33, -1.18) | 70.46 (33.24, 124.98) | 50.42 (26.04, 85.01) | -1.09 (-1.16, -1.02) |
| Bosnia and Herzegovina | 29.31 (20.87, 40.12) | 22.27 (17.11, 28.52) | -1.01 (-1.06, -0.96) | 233.69 (202.19, 268.92) | 190.05 (168.85, 212.00) | -0.79 (-0.84, -0.73) | 745.24 (433.23, 1218.47) | 326.11 (208.35, 472.76) | -3.13 (-3.36, -2.91) | 30.71 (16.80, 53.76) | 13.85 (8.20, 20.97) | -3.03 (-3.28, -2.77) |
| Botswana | 19.15 (12.73, 27.37) | 17.11 (11.55, 23.67) | -0.45 (-0.49, -0.42) | 289.14 (259.62, 320.84) | 269.07 (244.89, 294.48) | -0.28 (-0.29, -0.26) | 236.49 (138.12, 436.30) | 145.72 (91.25, 250.38) | -1.11 (-1.52, -0.70) | 9.31 (4.74, 18.82) | 5.22 (2.83, 10.08) | -1.32 (-1.80, -0.84) |
| Brazil | 31.58 (21.13, 45.49) | 27.74 (19.21, 38.60) | -0.70 (-0.80, -0.61) | 490.12 (397.85, 597.75) | 372.42 (305.47, 449.77) | -1.13 (-1.25, -1.01) | 675.92 (622.76, 731.78) | 573.03 (520.55, 622.87) | -0.57 (-0.84, -0.29) | 25.20 (22.95, 27.47) | 23.05 (20.54, 25.15) | -0.25 (-0.55, 0.06) |
| Brunei Darussalam | 100.27 (67.01, 136.75) | 63.21 (46.23, 83.05) | -1.96 (-2.13, -1.79) | 1060.21 (942.37, 1189.81) | 794.08 (725.71, 869.08) | -1.04 (-1.09, -0.98) | 1870.01 (1155.63, 2798.20) | 856.59 (575.37, 1210.85) | -2.80 (-2.97, -2.64) | 76.14 (43.60, 118.97) | 34.47 (21.17, 51.36) | -2.83 (-3.01, -2.65) |
| Bulgaria | 17.58 (13.31, 22.98) | 19.89 (15.46, 24.87) | 0.52 (0.46, 0.58) | 175.28 (154.53, 198.18) | 180.39 (160.67, 201.97) | 0.09 (0.08, 0.11) | 346.62 (288.11, 411.00) | 424.90 (352.56, 506.25) | 0.68 (0.51, 0.85) | 13.41 (11.10, 16.13) | 18.73 (15.53, 22.39) | 1.23 (1.02, 1.44) |
| Burkina Faso | 11.67 (7.17, 17.80) | 10.28 (6.55, 15.28) | -0.47 (-0.53, -0.41) | 248.73 (223.63, 276.40) | 227.06 (206.86, 248.11) | -0.33 (-0.35, -0.31) | 177.67 (85.93, 414.90) | 164.90 (83.28, 378.66) | 0.07 (-0.06, 0.20) | 6.36 (2.36, 17.38) | 6.17 (2.49, 16.41) | 0.36 (0.15, 0.56) |
| Burundi | 23.64 (15.54, 34.18) | 19.14 (12.42, 27.94) | -0.96 (-1.06, -0.87) | 382.34 (337.68, 428.53) | 310.25 (276.89, 343.62) | -0.75 (-0.79, -0.72) | 466.71 (185.90, 1131.23) | 274.65 (110.99, 822.00) | -2.21 (-2.41, -2.01) | 17.74 (5.69, 48.26) | 10.25 (3.14, 34.76) | -2.30 (-2.51, -2.08) |
| Cabo Verde | 12.69 (7.89, 19.56) | 11.01 (6.77, 16.82) | -0.56 (-0.63, -0.48) | 293.03 (265.16, 322.05) | 274.07 (250.77, 296.74) | -0.23 (-0.26, -0.20) | 188.21 (98.74, 469.05) | 158.73 (88.62, 349.10) | -0.53 (-0.68, -0.38) | 6.70 (2.75, 19.90) | 5.60 (2.47, 14.46) | -0.50 (-0.73, -0.28) |
| Cambodia | 40.72 (28.42, 56.09) | 32.99 (23.97, 43.83) | -0.87 (-0.93, -0.80) | 386.84 (334.95, 441.47) | 342.85 (304.43, 381.72) | -0.43 (-0.45, -0.41) | 1010.75 (478.01, 1855.09) | 616.72 (368.17, 1052.19) | -1.82 (-1.95, -1.69) | 44.16 (19.26, 87.08) | 27.53 (15.37, 51.90) | -1.74 (-1.88, -1.59) |
| Cameroon | 12.79 (8.18, 19.05) | 11.21 (7.28, 16.32) | -0.54 (-0.59, -0.48) | 260.23 (233.47, 288.99) | 237.61 (216.80, 259.47) | -0.34 (-0.36, -0.32) | 286.44 (153.51, 580.42) | 248.89 (130.07, 534.47) | -0.38 (-0.54, -0.22) | 11.33 (5.37, 25.43) | 9.71 (4.43, 22.76) | -0.41 (-0.58, -0.24) |
| Canada | 30.15 (21.60, 40.53) | 22.12 (15.52, 30.21) | -1.09 (-1.14, -1.05) | 483.69 (442.77, 526.04) | 387.35 (356.60, 417.34) | -0.80 (-0.85, -0.76) | 456.32 (381.17, 537.81) | 261.66 (214.29, 308.63) | -2.04 (-2.18, -1.91) | 17.43 (14.24, 20.88) | 10.35 (8.18, 12.57) | -1.91 (-2.07, -1.75) |
| Central African Republic | 20.55 (13.36, 29.96) | 19.14 (12.73, 27.12) | -0.29 (-0.32, -0.26) | 307.88 (270.52, 348.31) | 296.22 (261.37, 331.79) | -0.15 (-0.16, -0.13) | 417.06 (184.66, 976.13) | 369.25 (152.67, 922.52) | -0.43 (-0.51, -0.36) | 16.46 (6.22, 42.58) | 14.76 (5.00, 41.10) | -0.38 (-0.45, -0.31) |
| Chad | 13.09 (8.28, 19.87) | 11.99 (7.73, 17.39) | -0.35 (-0.40, -0.29) | 269.26 (240.23, 299.89) | 251.45 (228.69, 274.31) | -0.26 (-0.28, -0.25) | 310.68 (144.67, 737.70) | 329.91 (163.63, 726.62) | 0.24 (0.13, 0.34) | 12.10 (4.72, 31.97) | 13.00 (5.64, 31.38) | 0.30 (0.19, 0.41) |
| Chile | 35.21 (25.29, 47.67) | 30.28 (21.95, 40.07) | -0.25 (-0.36, -0.14) | 387.21 (330.91, 444.50) | 393.95 (332.99, 472.63) | 0.39 (0.20, 0.58) | 550.09 (460.71, 655.74) | 387.06 (320.09, 463.79) | -0.84 (-1.19, -0.49) | 20.68 (17.02, 25.01) | 15.72 (12.64, 19.09) | -0.58 (-0.99, -0.17) |
| China | 59.43 (40.20, 83.79) | 21.61 (15.05, 29.82) | -4.02 (-4.37, -3.66) | 328.35 (253.28, 415.28) | 207.70 (169.89, 252.12) | -1.60 (-1.71, -1.49) | 2431.63 (1037.13, 3166.38) | 393.73 (277.50, 527.76) | -7.00 (-7.77, -6.23) | 123.93 (49.51, 162.78) | 18.93 (12.62, 26.02) | -7.29 (-8.10, -6.46) |
| Colombia | 45.73 (33.28, 60.61) | 45.88 (33.84, 60.27) | -0.09 (-0.23, 0.06) | 794.26 (725.21, 869.62) | 685.00 (631.24, 741.61) | -0.57 (-0.60, -0.53) | 747.37 (626.78, 881.80) | 672.79 (536.96, 817.87) | -0.29 (-0.64, 0.05) | 29.58 (24.64, 35.21) | 28.25 (22.05, 34.91) | 0.05 (-0.36, 0.45) |
| Comoros | 21.11 (13.67, 30.46) | 17.17 (11.34, 24.66) | -0.84 (-0.91, -0.78) | 370.25 (331.24, 411.95) | 315.26 (284.91, 346.84) | -0.58 (-0.61, -0.55) | 358.02 (180.64, 788.73) | 245.30 (124.64, 546.09) | -1.42 (-1.58, -1.25) | 13.40 (5.61, 33.37) | 9.00 (3.67, 22.92) | -1.49 (-1.68, -1.31) |
| Congo | 19.16 (12.74, 27.31) | 15.54 (10.42, 22.36) | -0.85 (-0.92, -0.77) | 310.04 (274.29, 346.70) | 276.73 (249.61, 303.65) | -0.41 (-0.43, -0.38) | 366.96 (187.87, 822.46) | 294.44 (160.62, 613.30) | -0.93 (-1.08, -0.78) | 14.32 (6.51, 34.69) | 11.29 (5.43, 25.52) | -0.99 (-1.14, -0.83) |
| Cook Islands | 42.04 (30.07, 57.29) | 25.43 (17.84, 34.98) | -1.94 (-2.10, -1.79) | 520.62 (469.46, 574.77) | 403.50 (369.30, 440.08) | -0.90 (-0.95, -0.85) | 759.54 (456.68, 1215.56) | 288.13 (173.89, 462.76) | -3.25 (-3.49, -3.01) | 34.57 (18.84, 57.51) | 11.39 (5.34, 20.39) | -3.73 (-3.99, -3.46) |
| Costa Rica | 37.41 (26.71, 50.26) | 27.77 (19.87, 37.95) | -1.09 (-1.15, -1.02) | 654.13 (601.09, 707.21) | 551.47 (510.86, 593.19) | -0.63 (-0.67, -0.60) | 438.86 (357.30, 527.74) | 362.50 (290.91, 440.07) | -0.73 (-0.96, -0.50) | 16.89 (13.38, 20.77) | 14.64 (11.38, 18.27) | -0.58 (-0.83, -0.33) |
| Côte d'Ivoire | 12.25 (7.86, 18.55) | 10.92 (6.98, 16.09) | -0.43 (-0.48, -0.38) | 247.29 (222.37, 272.92) | 228.25 (207.44, 248.25) | -0.29 (-0.31, -0.28) | 250.02 (134.55, 505.70) | 225.78 (122.65, 474.68) | 0.10 (-0.08, 0.28) | 9.76 (4.60, 21.90) | 8.77 (4.21, 20.02) | 0.14 (-0.06, 0.35) |
| Croatia | 23.62 (18.11, 30.19) | 15.43 (12.17, 19.48) | -1.67 (-1.83, -1.52) | 203.20 (179.46, 228.11) | 155.43 (139.34, 172.80) | -1.02 (-1.07, -0.96) | 660.98 (567.32, 765.25) | 324.52 (264.86, 390.13) | -2.21 (-2.45, -1.97) | 27.57 (23.40, 32.06) | 14.73 (11.97, 17.69) | -1.85 (-2.11, -1.59) |
| Cuba | 27.94 (19.80, 38.21) | 21.69 (15.19, 29.65) | -0.99 (-1.06, -0.92) | 541.31 (496.45, 589.88) | 429.77 (396.04, 464.73) | -0.84 (-0.88, -0.80) | 444.20 (351.14, 547.32) | 304.81 (241.47, 376.79) | -1.43 (-1.62, -1.24) | 17.01 (13.01, 21.46) | 11.98 (9.34, 15.04) | -1.39 (-1.62, -1.16) |
| Cyprus | 34.64 (24.88, 46.71) | 20.35 (14.70, 27.37) | -2.39 (-2.62, -2.17) | 393.79 (348.47, 438.44) | 279.10 (250.82, 307.62) | -1.31 (-1.44, -1.17) | 749.61 (390.82, 1244.44) | 237.17 (150.97, 350.45) | -4.06 (-4.28, -3.84) | 39.80 (18.69, 68.99) | 10.84 (6.30, 16.86) | -4.53 (-4.83, -4.24) |
| Czechia | 23.19 (17.41, 30.38) | 14.28 (10.62, 18.83) | -1.87 (-2.02, -1.72) | 240.19 (212.28, 270.85) | 190.23 (172.76, 208.36) | -0.79 (-0.84, -0.73) | 596.55 (494.37, 703.86) | 223.84 (185.63, 267.07) | -3.24 (-3.52, -2.95) | 23.96 (19.83, 28.46) | 9.30 (7.66, 11.04) | -3.20 (-3.57, -2.82) |
| Democratic People's Republic of Korea | 46.68 (33.38, 63.74) | 32.81 (23.41, 45.09) | -1.42 (-1.53, -1.31) | 350.27 (303.16, 400.40) | 301.37 (264.57, 340.01) | -0.53 (-0.55, -0.51) | 1130.48 (612.27, 1810.90) | 705.87 (386.63, 1219.57) | -1.63 (-1.70, -1.56) | 52.71 (27.33, 87.36) | 31.18 (16.07, 57.15) | -1.80 (-1.87, -1.72) |
| Democratic Republic of the Congo | 19.04 (12.29, 27.68) | 19.05 (12.47, 27.66) | -0.01 (-0.08, 0.06) | 316.54 (279.74, 355.02) | 311.34 (277.65, 345.97) | -0.07 (-0.08, -0.06) | 317.90 (152.13, 731.06) | 301.03 (137.81, 737.60) | -0.14 (-0.23, -0.04) | 12.15 (4.85, 31.62) | 11.67 (4.31, 32.12) | -0.08 (-0.18, 0.02) |
| Denmark | 32.37 (23.80, 42.98) | 22.10 (15.98, 29.58) | -1.33 (-1.44, -1.22) | 466.18 (420.33, 515.64) | 359.25 (325.21, 393.90) | -0.77 (-0.80, -0.73) | 598.75 (488.68, 719.95) | 292.27 (237.48, 352.03) | -2.35 (-2.52, -2.17) | 23.47 (18.75, 28.75) | 12.58 (9.94, 15.33) | -2.03 (-2.24, -1.81) |
| Djibouti | 20.04 (13.19, 28.78) | 17.28 (11.37, 25.17) | -0.61 (-0.67, -0.54) | 339.73 (303.39, 377.55) | 305.27 (274.74, 337.52) | -0.38 (-0.41, -0.36) | 290.03 (150.17, 657.54) | 227.46 (116.25, 517.60) | -0.87 (-0.95, -0.79) | 10.77 (4.67, 27.35) | 8.31 (3.42, 21.52) | -0.93 (-1.01, -0.85) |
| Dominica | 25.53 (17.81, 35.21) | 20.86 (14.69, 28.58) | -0.76 (-0.85, -0.67) | 457.74 (415.44, 499.20) | 406.76 (373.52, 441.19) | -0.43 (-0.45, -0.40) | 522.07 (330.33, 785.09) | 381.69 (242.40, 570.04) | -1.01 (-1.16, -0.87) | 21.66 (13.17, 33.63) | 16.03 (9.52, 24.84) | -0.95 (-1.08, -0.81) |
| Dominican Republic | 26.85 (18.86, 36.96) | 31.33 (22.41, 42.16) | 0.71 (0.63, 0.80) | 502.87 (458.72, 550.56) | 545.06 (497.75, 594.08) | 0.33 (0.31, 0.36) | 496.01 (312.41, 767.90) | 440.14 (267.87, 678.44) | 0.00 (-0.18, 0.19) | 19.93 (11.62, 32.45) | 16.67 (9.30, 26.91) | -0.05 (-0.29, 0.19) |
| Ecuador | 42.69 (30.22, 57.73) | 42.12 (31.14, 56.67) | 0.12 (0.02, 0.22) | 798.78 (733.03, 868.20) | 712.54 (657.00, 771.28) | -0.42 (-0.45, -0.39) | 588.31 (478.64, 716.00) | 801.33 (611.97, 1039.37) | 1.11 (0.87, 1.35) | 21.37 (16.83, 26.80) | 34.09 (25.59, 44.40) | 1.66 (1.37, 1.94) |
| Egypt | 18.48 (12.23, 26.40) | 14.93 (10.73, 20.01) | -0.77 (-0.81, -0.73) | 224.35 (191.91, 261.08) | 185.08 (161.37, 211.29) | -0.68 (-0.71, -0.65) | 491.79 (187.82, 1181.45) | 288.70 (143.11, 553.25) | -1.48 (-1.62, -1.34) | 24.25 (8.05, 61.78) | 14.23 (6.31, 28.74) | -1.28 (-1.47, -1.09) |
| El Salvador | 37.06 (25.93, 50.70) | 28.34 (20.18, 38.62) | -0.89 (-1.05, -0.72) | 618.08 (563.56, 675.04) | 490.40 (452.23, 527.82) | -0.84 (-0.89, -0.78) | 562.68 (368.79, 857.88) | 373.59 (241.77, 551.43) | -1.13 (-1.49, -0.77) | 21.84 (13.56, 34.57) | 14.60 (8.86, 22.49) | -1.03 (-1.42, -0.64) |
| Equatorial Guinea | 20.40 (13.36, 29.46) | 13.52 (8.74, 19.56) | -1.63 (-1.78, -1.48) | 311.66 (276.31, 347.41) | 242.89 (218.73, 267.10) | -0.90 (-0.95, -0.86) | 346.71 (165.45, 826.24) | 174.90 (86.96, 341.60) | -2.74 (-3.06, -2.42) | 13.40 (5.37, 35.69) | 6.32 (2.47, 13.78) | -3.05 (-3.42, -2.68) |
| Eritrea | 23.03 (15.11, 33.73) | 19.99 (13.15, 29.21) | -0.56 (-0.61, -0.51) | 373.04 (330.19, 419.97) | 349.01 (314.10, 384.81) | -0.24 (-0.26, -0.23) | 423.16 (188.25, 1018.37) | 304.72 (154.01, 702.74) | -0.91 (-1.08, -0.74) | 16.01 (5.90, 43.45) | 11.46 (4.70, 29.89) | -0.93 (-1.11, -0.74) |
| Estonia | 19.33 (13.88, 26.26) | 14.70 (10.21, 20.25) | -1.10 (-1.20, -0.99) | 249.42 (210.84, 291.33) | 174.75 (152.86, 197.73) | -1.22 (-1.31, -1.12) | 267.77 (213.98, 330.83) | 175.99 (139.24, 216.76) | -2.16 (-2.93, -1.38) | 10.26 (8.15, 12.91) | 7.67 (5.95, 9.45) | -1.85 (-2.77, -0.91) |
| Eswatini | 16.90 (11.25, 24.34) | 15.45 (10.66, 21.46) | -0.31 (-0.47, -0.14) | 259.51 (230.36, 289.01) | 236.64 (215.09, 259.50) | -0.34 (-0.36, -0.32) | 229.17 (135.34, 410.40) | 217.48 (122.80, 376.22) | 0.13 (-0.34, 0.61) | 9.21 (4.85, 17.73) | 8.74 (4.36, 15.99) | 0.18 (-0.32, 0.69) |
| Ethiopia | 23.95 (15.56, 35.05) | 14.37 (9.47, 20.75) | -2.12 (-2.32, -1.92) | 390.51 (317.57, 477.19) | 242.23 (202.93, 289.03) | -1.69 (-1.76, -1.62) | 338.13 (164.53, 810.78) | 169.93 (92.41, 422.34) | -2.64 (-2.80, -2.49) | 12.30 (4.70, 34.01) | 6.06 (2.66, 17.66) | -2.76 (-2.93, -2.59) |
| Fiji | 80.01 (59.43, 104.85) | 64.01 (48.56, 82.23) | -0.94 (-1.01, -0.86) | 709.52 (622.20, 804.65) | 614.24 (550.28, 685.19) | -0.55 (-0.59, -0.52) | 1120.93 (731.64, 1622.99) | 849.58 (544.17, 1252.23) | -1.06 (-1.19, -0.93) | 48.41 (30.05, 72.73) | 37.48 (22.42, 57.61) | -1.01 (-1.17, -0.85) |
| Finland | 38.75 (30.97, 47.48) | 29.80 (21.94, 39.17) | -1.06 (-1.31, -0.81) | 669.52 (603.65, 732.31) | 604.01 (558.92, 654.01) | -0.22 (-0.40, -0.04) | 667.67 (543.46, 801.82) | 366.02 (299.13, 437.24) | -1.96 (-2.05, -1.88) | 25.54 (20.11, 31.64) | 13.96 (10.98, 16.96) | -2.05 (-2.17, -1.93) |
| France | 15.95 (11.63, 21.55) | 17.27 (12.51, 23.24) | 0.47 (0.38, 0.56) | 266.74 (241.52, 293.84) | 303.65 (275.80, 333.02) | 0.47 (0.44, 0.50) | 253.86 (208.39, 303.51) | 199.67 (161.70, 241.17) | -0.47 (-0.76, -0.19) | 11.24 (9.05, 13.49) | 8.07 (6.31, 9.90) | -0.72 (-1.11, -0.32) |
| Gabon | 18.66 (12.34, 26.72) | 14.78 (9.54, 21.04) | -0.98 (-1.08, -0.87) | 313.60 (279.91, 348.64) | 276.18 (250.77, 303.14) | -0.44 (-0.48, -0.40) | 240.75 (131.27, 492.10) | 192.99 (106.69, 364.66) | -0.84 (-1.04, -0.63) | 8.93 (4.10, 20.44) | 7.10 (3.22, 14.98) | -0.88 (-1.11, -0.66) |
| Gambia | 12.06 (7.63, 17.97) | 11.11 (7.07, 16.48) | -0.33 (-0.36, -0.29) | 255.21 (230.02, 281.88) | 242.45 (220.88, 265.67) | -0.19 (-0.20, -0.18) | 272.55 (145.90, 583.12) | 295.29 (149.97, 667.89) | 0.21 (0.04, 0.38) | 10.55 (4.91, 24.90) | 11.58 (5.27, 28.37) | 0.29 (0.13, 0.45) |
| Georgia | 38.62 (28.93, 50.33) | 43.80 (34.42, 55.23) | 0.94 (0.72, 1.15) | 312.46 (276.64, 349.88) | 336.10 (299.88, 374.21) | 0.22 (0.19, 0.24) | 481.55 (348.63, 602.84) | 712.09 (583.73, 840.45) | 2.60 (1.70, 3.52) | 20.55 (14.04, 26.45) | 33.82 (27.38, 40.32) | 3.33 (2.27, 4.41) |
| Germany | 25.07 (18.21, 33.43) | 17.65 (12.33, 24.27) | -1.36 (-1.46, -1.25) | 404.69 (363.67, 447.09) | 330.98 (302.11, 360.58) | -0.65 (-0.69, -0.61) | 386.23 (310.25, 474.74) | 237.51 (191.41, 287.63) | -1.67 (-1.85, -1.49) | 15.58 (12.17, 19.69) | 9.24 (7.14, 11.38) | -1.82 (-2.06, -1.58) |
| Ghana | 13.83 (8.79, 20.40) | 12.09 (7.92, 17.60) | -0.46 (-0.54, -0.38) | 322.94 (293.11, 356.40) | 286.35 (263.34, 311.72) | -0.43 (-0.45, -0.41) | 363.53 (195.79, 805.51) | 298.56 (166.18, 580.58) | -0.42 (-0.67, -0.16) | 13.97 (6.64, 33.76) | 11.62 (5.79, 24.36) | -0.32 (-0.61, -0.02) |
| Greece | 21.94 (15.27, 30.48) | 19.89 (13.93, 27.37) | -0.21 (-0.45, 0.02) | 331.59 (298.99, 364.85) | 364.10 (330.56, 398.03) | 0.38 (0.35, 0.41) | 331.93 (231.98, 433.43) | 267.21 (216.67, 322.06) | -0.73 (-0.99, -0.46) | 14.81 (9.75, 19.78) | 11.16 (8.76, 13.56) | -1.05 (-1.37, -0.74) |
| Greenland | 39.42 (24.44, 73.36) | 37.40 (28.85, 47.79) | -0.11 (-0.26, 0.04) | 534.87 (430.54, 826.60) | 445.18 (400.60, 491.22) | -0.68 (-0.75, -0.61) | 2200.22 (1410.53, 3153.39) | 799.06 (504.47, 1180.65) | -3.65 (-3.84, -3.46) | 93.83 (57.70, 136.13) | 34.24 (20.55, 51.95) | -3.63 (-3.83, -3.44) |
| Grenada | 35.50 (25.68, 47.34) | 28.73 (21.14, 38.12) | -0.76 (-0.90, -0.63) | 508.69 (460.37, 560.24) | 454.94 (416.59, 496.11) | -0.44 (-0.47, -0.42) | 698.08 (506.39, 914.07) | 480.49 (382.81, 593.66) | -1.02 (-1.33, -0.71) | 26.29 (18.71, 34.73) | 19.50 (15.35, 24.37) | -0.86 (-1.18, -0.55) |
| Guam | 39.47 (28.40, 53.22) | 24.79 (17.74, 33.58) | -1.83 (-2.00, -1.67) | 454.73 (409.16, 502.72) | 409.93 (375.24, 444.50) | -0.32 (-0.35, -0.29) | 707.96 (454.60, 1007.28) | 312.40 (220.22, 427.69) | -2.72 (-2.99, -2.45) | 33.75 (19.30, 49.87) | 9.97 (6.21, 14.79) | -3.85 (-4.23, -3.48) |
| Guatemala | 21.43 (14.33, 30.34) | 21.06 (14.70, 29.19) | 0.05 (-0.01, 0.12) | 453.95 (416.13, 493.39) | 424.61 (390.24, 459.31) | -0.25 (-0.27, -0.23) | 202.21 (155.56, 262.79) | 243.76 (190.07, 304.88) | 0.47 (0.23, 0.70) | 6.96 (5.11, 9.38) | 9.03 (6.83, 11.59) | 0.65 (0.38, 0.92) |
| Guinea | 13.59 (8.63, 20.31) | 11.96 (7.78, 17.47) | -0.48 (-0.51, -0.45) | 274.10 (246.78, 304.06) | 254.41 (231.66, 277.47) | -0.28 (-0.29, -0.26) | 328.84 (165.43, 746.25) | 305.08 (152.03, 713.05) | 0.02 (-0.08, 0.12) | 12.74 (5.59, 31.59) | 11.95 (5.24, 30.98) | 0.09 (-0.02, 0.20) |
| Guinea-Bissau | 13.42 (8.73, 19.73) | 11.57 (7.37, 16.89) | -0.55 (-0.59, -0.52) | 284.84 (256.23, 315.30) | 252.45 (229.57, 275.40) | -0.44 (-0.47, -0.42) | 424.90 (195.82, 968.75) | 392.81 (194.57, 893.38) | -0.07 (-0.17, 0.02) | 16.60 (6.61, 41.72) | 15.71 (6.99, 38.91) | 0.04 (-0.07, 0.14) |
| Guyana | 37.38 (27.48, 50.45) | 28.00 (20.61, 36.93) | -1.29 (-1.43, -1.15) | 476.93 (424.43, 531.12) | 427.19 (386.08, 468.43) | -0.43 (-0.47, -0.40) | 645.91 (512.86, 796.35) | 470.76 (340.18, 630.46) | -0.69 (-0.89, -0.50) | 25.35 (19.96, 31.40) | 18.38 (12.97, 24.92) | -0.68 (-0.89, -0.47) |
| Haiti | 41.47 (28.04, 58.03) | 39.61 (27.60, 53.74) | -0.27 (-0.33, -0.20) | 510.10 (439.27, 584.44) | 482.29 (420.33, 547.63) | -0.21 (-0.26, -0.16) | 1509.99 (473.68, 2805.33) | 1225.79 (423.43, 2380.62) | -0.54 (-0.65, -0.42) | 61.92 (18.58, 120.90) | 51.12 (16.39, 102.98) | -0.49 (-0.60, -0.37) |
| Honduras | 32.63 (22.21, 46.44) | 41.36 (30.12, 56.08) | 0.86 (0.74, 0.97) | 540.17 (485.31, 599.43) | 513.86 (461.62, 566.42) | -0.22 (-0.24, -0.20) | 763.75 (421.24, 1414.96) | 1377.69 (772.06, 2171.41) | 2.19 (1.87, 2.51) | 31.17 (16.12, 61.25) | 60.32 (32.68, 96.16) | 2.43 (2.05, 2.80) |
| Hungary | 16.12 (12.14, 21.31) | 13.68 (10.23, 17.99) | -0.66 (-0.70, -0.61) | 214.99 (192.38, 239.12) | 167.17 (149.84, 186.09) | -0.88 (-0.94, -0.82) | 338.10 (291.57, 390.04) | 222.86 (182.58, 267.12) | -1.30 (-1.56, -1.05) | 12.57 (10.76, 14.51) | 8.43 (6.82, 10.14) | -1.24 (-1.55, -0.93) |
| Iceland | 31.66 (22.70, 43.64) | 19.74 (13.41, 27.57) | -2.01 (-2.17, -1.85) | 470.54 (426.91, 519.00) | 350.39 (319.67, 382.63) | -1.00 (-1.04, -0.95) | 451.86 (369.37, 543.28) | 219.17 (174.18, 266.95) | -2.29 (-2.62, -1.96) | 18.33 (14.50, 22.26) | 9.46 (7.14, 11.71) | -2.03 (-2.43, -1.63) |
| India | 28.56 (19.25, 40.65) | 18.42 (12.65, 25.54) | -1.82 (-1.98, -1.65) | 211.95 (165.79, 267.59) | 172.60 (138.63, 213.71) | -0.72 (-0.79, -0.65) | 514.26 (266.67, 958.65) | 381.93 (283.16, 521.80) | -1.07 (-1.22, -0.92) | 20.12 (9.83, 40.56) | 14.86 (10.64, 21.26) | -1.06 (-1.19, -0.92) |
| Indonesia | 37.72 (25.38, 53.34) | 31.08 (21.68, 43.26) | -0.75 (-0.82, -0.69) | 442.42 (356.54, 544.19) | 366.05 (299.48, 445.18) | -0.70 (-0.74, -0.66) | 925.88 (540.07, 1543.85) | 705.01 (444.64, 1191.89) | -0.90 (-1.02, -0.78) | 38.99 (21.16, 72.79) | 30.89 (18.02, 58.19) | -0.77 (-0.90, -0.64) |
| Iran (Islamic Republic of) | 18.10 (12.19, 25.77) | 11.80 (8.07, 16.65) | -1.61 (-1.72, -1.50) | 215.67 (177.94, 259.16) | 173.04 (146.18, 203.24) | -0.80 (-0.85, -0.75) | 328.19 (196.43, 511.47) | 147.23 (105.94, 187.01) | -2.80 (-2.98, -2.61) | 15.53 (8.17, 25.57) | 6.76 (4.29, 8.79) | -2.88 (-3.07, -2.69) |
| Iraq | 24.23 (17.23, 32.70) | 11.43 (8.01, 15.69) | -2.98 (-3.28, -2.69) | 277.49 (243.71, 314.84) | 188.27 (168.68, 207.84) | -1.37 (-1.45, -1.30) | 365.64 (214.40, 652.24) | 219.54 (133.62, 346.07) | -2.22 (-2.43, -2.02) | 14.44 (7.77, 27.93) | 9.11 (5.16, 14.85) | -2.14 (-2.38, -1.91) |
| Ireland | 42.32 (31.18, 56.96) | 24.45 (17.81, 32.57) | -2.18 (-2.34, -2.03) | 587.85 (527.43, 652.62) | 377.98 (343.01, 414.97) | -1.61 (-1.71, -1.51) | 740.24 (621.96, 867.68) | 266.40 (212.83, 320.42) | -3.19 (-3.41, -2.97) | 30.13 (25.03, 35.63) | 11.91 (9.14, 14.53) | -2.78 (-3.01, -2.55) |
| Israel | 19.58 (13.64, 27.24) | 15.08 (10.37, 20.82) | -1.06 (-1.14, -0.98) | 318.03 (284.25, 352.38) | 268.62 (244.04, 295.85) | -0.58 (-0.61, -0.54) | 204.75 (166.09, 247.46) | 120.01 (93.44, 147.44) | -1.79 (-2.05, -1.53) | 7.35 (5.80, 9.13) | 4.42 (3.40, 5.44) | -1.60 (-2.03, -1.16) |
| Italy | 19.13 (12.59, 28.17) | 15.00 (10.88, 19.83) | -0.79 (-0.86, -0.72) | 245.85 (202.19, 295.37) | 217.62 (182.43, 259.10) | -0.37 (-0.40, -0.33) | 281.04 (256.22, 304.49) | 175.55 (151.98, 195.78) | -1.24 (-1.45, -1.04) | 11.24 (10.08, 12.16) | 7.84 (6.45, 8.79) | -0.78 (-1.09, -0.46) |
| Jamaica | 36.04 (23.77, 51.59) | 33.13 (24.44, 43.78) | -0.37 (-0.45, -0.28) | 573.98 (518.98, 636.35) | 541.25 (496.68, 588.17) | -0.18 (-0.19, -0.16) | 660.86 (505.55, 835.34) | 504.20 (378.56, 652.24) | -0.59 (-0.87, -0.31) | 25.59 (18.95, 32.92) | 20.53 (14.91, 27.08) | -0.44 (-0.72, -0.15) |
| Japan | 67.95 (46.35, 95.59) | 58.43 (41.71, 79.44) | -0.59 (-0.72, -0.47) | 864.49 (711.48, 1049.61) | 993.60 (845.01, 1171.08) | 0.48 (0.44, 0.52) | 933.44 (855.58, 1005.58) | 495.86 (425.59, 563.86) | -2.38 (-2.50, -2.27) | 37.00 (33.28, 39.44) | 16.89 (13.58, 18.95) | -2.97 (-3.13, -2.80) |
| Jordan | 14.84 (10.32, 20.62) | 9.09 (6.28, 12.79) | -2.02 (-2.22, -1.82) | 221.69 (196.78, 251.14) | 165.14 (148.44, 183.00) | -1.05 (-1.12, -0.99) | 121.83 (78.13, 190.47) | 69.20 (45.85, 98.66) | -2.05 (-2.33, -1.77) | 4.16 (2.35, 7.24) | 2.29 (1.33, 3.65) | -2.13 (-2.51, -1.75) |
| Kazakhstan | 25.81 (18.84, 34.70) | 25.72 (19.72, 33.26) | -0.03 (-0.05, -0.01) | 227.89 (198.02, 259.09) | 216.64 (192.30, 242.78) | -0.22 (-0.24, -0.20) | 525.47 (404.59, 698.08) | 537.63 (454.07, 630.90) | -0.36 (-0.61, -0.10) | 22.62 (16.82, 31.28) | 25.66 (21.80, 30.08) | 0.03 (-0.20, 0.26) |
| Kenya | 17.67 (11.53, 25.84) | 14.76 (9.80, 21.25) | -0.65 (-0.71, -0.58) | 280.81 (233.39, 338.67) | 255.73 (215.91, 304.46) | -0.33 (-0.37, -0.30) | 197.15 (111.51, 495.79) | 184.51 (107.29, 445.55) | 0.31 (0.09, 0.53) | 7.19 (3.38, 20.97) | 6.86 (3.38, 18.80) | 0.50 (0.21, 0.79) |
| Kiribati | 58.31 (41.64, 78.91) | 53.98 (39.72, 70.33) | -0.35 (-0.38, -0.32) | 593.75 (517.23, 679.38) | 591.15 (524.81, 661.86) | -0.02 (-0.05, 0.01) | 645.74 (403.48, 978.04) | 621.35 (385.18, 975.85) | -0.08 (-0.13, -0.03) | 25.87 (14.00, 42.40) | 25.77 (14.35, 43.04) | 0.05 (-0.01, 0.12) |
| Kuwait | 14.20 (9.57, 20.18) | 12.31 (8.55, 17.24) | -0.53 (-0.58, -0.48) | 262.24 (228.66, 293.14) | 230.15 (206.51, 252.33) | -0.50 (-0.57, -0.43) | 248.17 (191.51, 317.78) | 62.16 (44.84, 82.97) | -4.80 (-5.52, -4.08) | 9.42 (6.95, 12.30) | 1.42 (1.04, 1.85) | -6.67 (-7.69, -5.65) |
| Kyrgyzstan | 25.08 (18.22, 33.31) | 20.30 (15.03, 26.66) | -0.71 (-0.78, -0.63) | 271.43 (236.65, 308.42) | 221.97 (196.00, 249.04) | -0.72 (-0.74, -0.69) | 486.25 (398.44, 591.31) | 459.78 (368.73, 566.63) | -0.09 (-0.64, 0.45) | 20.06 (16.06, 25.18) | 19.53 (15.47, 24.31) | 0.10 (-0.39, 0.59) |
| Lao People's Democratic Republic | 38.65 (26.45, 53.51) | 27.12 (19.33, 36.74) | -1.45 (-1.57, -1.32) | 387.34 (335.80, 443.13) | 318.16 (281.93, 354.77) | -0.71 (-0.74, -0.68) | 1287.28 (500.48, 2368.96) | 578.81 (326.51, 999.70) | -2.81 (-2.89, -2.72) | 54.13 (18.96, 106.65) | 24.59 (12.60, 46.78) | -2.77 (-2.86, -2.68) |
| Latvia | 24.49 (17.80, 32.53) | 15.80 (11.28, 21.56) | -1.66 (-1.76, -1.56) | 236.04 (205.37, 268.63) | 184.46 (162.67, 206.73) | -0.53 (-0.60, -0.46) | 380.33 (309.19, 461.36) | 265.98 (214.97, 326.55) | -1.92 (-2.45, -1.40) | 15.07 (12.03, 18.42) | 11.42 (9.19, 14.01) | -1.74 (-2.38, -1.10) |
| Lebanon | 21.81 (14.83, 30.48) | 13.42 (9.16, 18.91) | -1.96 (-2.10, -1.82) | 286.20 (251.66, 322.87) | 218.64 (194.94, 242.22) | -0.94 (-0.98, -0.90) | 624.64 (308.81, 1098.23) | 175.11 (111.82, 265.28) | -4.65 (-4.92, -4.38) | 28.36 (13.19, 51.85) | 7.70 (4.42, 12.44) | -4.78 (-5.04, -4.52) |
| Lesotho | 16.80 (11.06, 24.34) | 20.27 (14.15, 27.79) | 0.83 (0.68, 0.98) | 249.32 (221.17, 278.96) | 272.98 (246.42, 300.54) | 0.30 (0.29, 0.31) | 210.65 (102.99, 424.91) | 307.65 (160.75, 573.95) | 2.18 (1.66, 2.71) | 8.47 (3.44, 18.84) | 12.69 (5.96, 25.09) | 2.36 (1.78, 2.94) |
| Liberia | 13.02 (8.30, 19.56) | 11.28 (7.25, 16.72) | -0.62 (-0.68, -0.57) | 268.03 (239.93, 296.67) | 240.72 (218.28, 263.82) | -0.40 (-0.41, -0.38) | 315.99 (165.89, 675.53) | 295.21 (133.84, 773.75) | -0.30 (-0.43, -0.17) | 12.34 (5.77, 28.89) | 11.56 (4.57, 33.94) | -0.29 (-0.43, -0.15) |
| Libya | 16.57 (10.81, 24.55) | 11.37 (7.80, 15.92) | -1.47 (-1.62, -1.32) | 227.89 (198.74, 261.09) | 191.25 (170.70, 213.52) | -0.62 (-0.65, -0.59) | 255.19 (136.18, 469.74) | 230.89 (117.47, 403.73) | -0.14 (-0.30, 0.02) | 10.42 (4.90, 21.12) | 9.80 (4.55, 18.12) | 0.13 (-0.05, 0.30) |
| Lithuania | 20.32 (14.97, 26.69) | 15.10 (10.92, 20.51) | -0.87 (-0.94, -0.80) | 191.42 (167.03, 217.97) | 194.79 (169.21, 221.13) | 0.31 (0.23, 0.40) | 258.62 (206.69, 318.94) | 244.18 (196.63, 299.27) | -0.54 (-1.25, 0.19) | 9.93 (7.86, 12.36) | 10.41 (8.38, 12.69) | -0.36 (-1.20, 0.48) |
| Luxembourg | 19.90 (14.18, 26.78) | 14.36 (9.94, 19.97) | -1.49 (-1.77, -1.21) | 253.19 (226.68, 280.78) | 213.54 (193.28, 234.88) | -0.67 (-0.82, -0.51) | 325.10 (269.71, 391.15) | 158.78 (127.16, 193.26) | -2.35 (-2.71, -2.00) | 13.70 (11.25, 16.62) | 6.72 (5.25, 8.30) | -2.25 (-2.67, -1.84) |
| Madagascar | 23.08 (15.07, 33.08) | 22.04 (14.67, 31.35) | -0.26 (-0.32, -0.20) | 415.70 (368.34, 467.36) | 389.01 (349.13, 431.15) | -0.24 (-0.26, -0.22) | 463.62 (222.25, 1143.58) | 428.65 (204.36, 1011.86) | -0.31 (-0.36, -0.26) | 17.60 (7.13, 48.14) | 16.25 (6.56, 42.74) | -0.32 (-0.37, -0.26) |
| Malawi | 19.32 (12.27, 28.79) | 16.86 (10.93, 24.59) | -0.58 (-0.63, -0.53) | 294.60 (259.64, 331.82) | 262.21 (233.37, 290.52) | -0.43 (-0.45, -0.41) | 328.76 (146.99, 870.18) | 285.66 (125.80, 779.96) | -0.61 (-0.73, -0.49) | 12.82 (4.69, 37.95) | 11.20 (4.05, 34.02) | -0.59 (-0.71, -0.46) |
| Malaysia | 46.16 (32.85, 62.43) | 27.63 (20.11, 37.26) | -1.89 (-1.98, -1.79) | 467.32 (413.93, 523.02) | 370.35 (334.68, 406.33) | -0.82 (-0.87, -0.77) | 724.07 (464.07, 1098.05) | 439.38 (287.52, 650.14) | -1.62 (-1.71, -1.53) | 30.42 (17.82, 49.18) | 19.21 (11.25, 30.06) | -1.41 (-1.54, -1.27) |
| Maldives | 48.05 (34.47, 64.79) | 25.08 (17.97, 33.89) | -2.57 (-2.74, -2.39) | 478.20 (422.84, 536.75) | 308.42 (279.84, 337.41) | -1.58 (-1.66, -1.50) | 977.00 (429.15, 1606.28) | 296.79 (198.62, 433.65) | -4.32 (-4.54, -4.10) | 42.27 (16.37, 73.77) | 13.13 (7.92, 20.25) | -4.26 (-4.48, -4.05) |
| Mali | 12.45 (7.75, 18.76) | 9.84 (6.29, 14.71) | -1.00 (-1.09, -0.91) | 253.39 (226.57, 280.71) | 218.97 (199.82, 239.53) | -0.52 (-0.55, -0.50) | 343.50 (165.58, 756.95) | 270.90 (139.14, 554.91) | -0.71 (-0.79, -0.63) | 13.54 (5.57, 32.85) | 10.65 (4.85, 23.58) | -0.69 (-0.78, -0.60) |
| Malta | 22.30 (15.23, 31.01) | 16.28 (10.87, 23.08) | -1.48 (-1.63, -1.33) | 373.46 (333.15, 416.03) | 311.09 (281.40, 340.44) | -0.66 (-0.72, -0.61) | 248.98 (200.27, 301.98) | 136.14 (107.35, 168.26) | -1.89 (-2.12, -1.66) | 9.02 (7.21, 11.04) | 4.61 (3.49, 5.85) | -2.18 (-2.53, -1.82) |
| Marshall Islands | 68.22 (48.35, 92.86) | 62.27 (46.45, 81.55) | -0.43 (-0.52, -0.33) | 601.57 (518.35, 690.32) | 577.09 (502.62, 652.06) | -0.18 (-0.21, -0.14) | 1367.91 (665.51, 2474.11) | 1069.42 (527.60, 1817.55) | -0.99 (-1.12, -0.85) | 63.63 (28.70, 120.25) | 48.26 (21.65, 85.77) | -1.09 (-1.22, -0.95) |
| Mauritania | 13.52 (8.61, 19.79) | 10.77 (6.84, 16.00) | -0.89 (-0.95, -0.83) | 286.86 (258.67, 315.31) | 245.79 (222.37, 269.50) | -0.55 (-0.58, -0.53) | 365.66 (185.55, 821.27) | 263.00 (134.85, 579.21) | -1.10 (-1.21, -0.99) | 14.17 (6.40, 34.01) | 10.14 (4.53, 24.14) | -1.09 (-1.22, -0.97) |
| Mauritius | 31.95 (23.14, 42.74) | 24.66 (17.90, 32.82) | -1.22 (-1.37, -1.08) | 480.20 (419.95, 549.04) | 437.75 (382.70, 487.21) | -0.13 (-0.21, -0.06) | 715.25 (594.54, 854.43) | 498.92 (410.50, 597.63) | -2.12 (-2.65, -1.59) | 29.01 (23.71, 35.11) | 21.00 (16.87, 25.36) | -2.05 (-2.58, -1.51) |
| Mexico | 26.92 (17.98, 38.97) | 28.69 (19.84, 40.15) | 0.24 (0.16, 0.31) | 510.93 (427.68, 606.36) | 464.68 (391.39, 550.22) | -0.31 (-0.34, -0.28) | 338.30 (314.81, 364.13) | 402.05 (345.17, 463.15) | 0.65 (0.42, 0.87) | 13.39 (12.45, 14.36) | 16.91 (14.36, 19.52) | 0.90 (0.65, 1.16) |
| Micronesia (Federated States of) | 69.20 (48.69, 94.19) | 60.87 (45.24, 79.41) | -0.55 (-0.62, -0.49) | 609.22 (525.22, 692.68) | 569.45 (498.65, 639.55) | -0.25 (-0.28, -0.22) | 1480.93 (748.19, 2654.59) | 991.85 (532.49, 1688.50) | -1.38 (-1.52, -1.24) | 67.92 (31.29, 125.39) | 44.56 (21.88, 78.98) | -1.45 (-1.59, -1.30) |
| Monaco | 24.25 (17.00, 33.56) | 15.82 (10.74, 22.13) | -1.58 (-1.70, -1.45) | 345.96 (309.42, 385.63) | 280.30 (253.74, 307.75) | -0.74 (-0.77, -0.70) | 491.63 (288.65, 813.59) | 214.66 (131.38, 331.23) | -2.68 (-2.80, -2.56) | 20.73 (11.20, 35.96) | 8.57 (4.61, 14.25) | -2.86 (-3.00, -2.71) |
| Mongolia | 33.01 (23.66, 44.30) | 38.74 (29.07, 50.04) | 0.42 (0.20, 0.64) | 258.04 (220.46, 299.52) | 244.68 (211.29, 280.34) | -0.22 (-0.24, -0.19) | 980.86 (549.24, 1889.30) | 979.67 (641.93, 1432.20) | -0.09 (-0.31, 0.14) | 46.23 (25.07, 91.41) | 48.84 (31.04, 72.41) | 0.08 (-0.15, 0.32) |
| Montenegro | 13.62 (9.71, 18.56) | 12.77 (9.52, 16.80) | -0.11 (-0.24, 0.02) | 152.93 (134.91, 172.14) | 138.23 (122.63, 155.00) | -0.38 (-0.41, -0.35) | 233.96 (159.85, 332.07) | 231.57 (145.51, 343.34) | -0.19 (-0.53, 0.15) | 9.58 (6.17, 13.98) | 10.58 (6.04, 16.16) | 0.17 (-0.14, 0.49) |
| Morocco | 22.21 (14.80, 31.52) | 17.44 (12.52, 23.64) | -0.96 (-1.05, -0.88) | 270.80 (232.71, 310.56) | 222.49 (197.27, 249.06) | -0.72 (-0.75, -0.69) | 501.50 (191.67, 1247.69) | 370.10 (166.31, 659.28) | -0.85 (-0.91, -0.79) | 21.54 (7.21, 58.65) | 16.17 (6.48, 29.89) | -0.73 (-0.81, -0.65) |
| Mozambique | 21.40 (13.55, 31.76) | 20.05 (13.23, 28.80) | -0.22 (-0.26, -0.18) | 318.80 (278.44, 359.91) | 310.69 (277.21, 346.30) | -0.09 (-0.11, -0.08) | 369.56 (160.97, 1068.92) | 358.19 (157.59, 945.68) | 0.27 (0.07, 0.47) | 14.45 (5.16, 47.02) | 14.01 (5.07, 40.96) | 0.33 (0.10, 0.55) |
| Myanmar | 38.66 (26.62, 53.21) | 24.60 (17.63, 33.38) | -1.82 (-2.00, -1.63) | 390.63 (338.04, 444.97) | 289.28 (257.39, 323.25) | -1.08 (-1.13, -1.03) | 1052.47 (520.65, 1987.43) | 502.03 (304.24, 843.79) | -2.87 (-3.07, -2.67) | 43.95 (20.16, 89.90) | 21.28 (11.84, 39.47) | -2.84 (-3.05, -2.64) |
| Namibia | 19.84 (13.37, 27.92) | 16.21 (11.26, 22.35) | -0.91 (-1.01, -0.80) | 304.69 (273.82, 336.76) | 255.79 (233.08, 280.07) | -0.64 (-0.68, -0.61) | 241.15 (143.08, 432.44) | 186.02 (108.48, 329.81) | -1.13 (-1.37, -0.89) | 9.29 (4.82, 18.14) | 7.27 (3.69, 13.84) | -1.11 (-1.38, -0.84) |
| Nauru | 57.22 (40.45, 77.24) | 34.23 (24.26, 46.68) | -1.94 (-2.06, -1.82) | 585.62 (504.93, 668.66) | 527.54 (455.67, 602.24) | -0.39 (-0.41, -0.36) | 1521.28 (765.60, 2730.39) | 1154.26 (605.20, 1871.27) | -0.96 (-1.28, -0.64) | 68.57 (32.28, 127.65) | 50.56 (23.69, 85.01) | -1.05 (-1.36, -0.73) |
| Nepal | 30.19 (19.87, 43.40) | 25.07 (17.24, 34.79) | -0.74 (-0.81, -0.67) | 277.64 (229.64, 325.61) | 244.72 (209.53, 282.88) | -0.43 (-0.45, -0.41) | 751.00 (299.44, 1951.29) | 434.35 (230.61, 841.56) | -1.79 (-2.07, -1.51) | 30.05 (10.85, 86.02) | 17.57 (8.50, 36.75) | -1.75 (-2.04, -1.46) |
| Netherlands | 27.96 (20.04, 38.09) | 19.53 (13.97, 26.74) | -1.11 (-1.21, -1.00) | 429.71 (387.73, 471.66) | 341.45 (310.60, 373.78) | -0.80 (-0.83, -0.76) | 461.75 (385.57, 546.81) | 243.22 (197.74, 291.94) | -2.29 (-2.50, -2.08) | 18.70 (15.25, 22.49) | 10.22 (8.00, 12.41) | -2.25 (-2.48, -2.01) |
| New Zealand | 31.04 (20.12, 45.45) | 18.71 (13.26, 25.52) | -1.85 (-2.00, -1.70) | 356.15 (286.65, 440.98) | 263.80 (216.62, 317.24) | -1.17 (-1.29, -1.05) | 638.14 (542.77, 740.13) | 305.91 (251.89, 361.18) | -2.61 (-2.85, -2.38) | 25.90 (21.62, 30.50) | 13.14 (10.53, 15.73) | -2.39 (-2.65, -2.13) |
| Nicaragua | 37.80 (26.68, 51.99) | 31.21 (21.76, 42.24) | -0.73 (-0.78, -0.68) | 608.28 (555.09, 664.02) | 524.64 (482.64, 567.24) | -0.53 (-0.56, -0.50) | 438.12 (295.80, 629.71) | 338.56 (221.90, 488.49) | -0.53 (-0.72, -0.34) | 16.41 (10.29, 25.14) | 12.51 (7.50, 19.07) | -0.49 (-0.72, -0.25) |
| Niger | 13.42 (8.43, 20.28) | 12.26 (7.85, 18.14) | -0.42 (-0.46, -0.37) | 282.09 (252.63, 313.04) | 254.85 (230.00, 279.35) | -0.38 (-0.39, -0.36) | 316.98 (149.87, 704.34) | 277.18 (132.39, 699.44) | -0.47 (-0.51, -0.42) | 12.40 (4.91, 31.09) | 10.96 (4.39, 30.76) | -0.40 (-0.44, -0.35) |
| Nigeria | 12.42 (8.05, 18.44) | 9.52 (6.22, 13.97) | -1.12 (-1.23, -1.02) | 248.23 (208.69, 294.31) | 210.03 (179.85, 245.42) | -0.60 (-0.63, -0.56) | 259.28 (150.39, 537.82) | 158.89 (101.61, 318.03) | -2.00 (-2.19, -1.80) | 10.42 (5.38, 23.60) | 5.99 (3.43, 13.31) | -2.27 (-2.49, -2.06) |
| Niue | 54.04 (38.60, 73.25) | 37.55 (27.07, 50.28) | -1.39 (-1.47, -1.31) | 579.59 (515.55, 648.80) | 489.66 (440.10, 540.59) | -0.60 (-0.62, -0.57) | 962.95 (581.36, 1498.54) | 607.50 (373.70, 931.30) | -1.74 (-1.86, -1.63) | 44.33 (24.83, 70.94) | 26.86 (15.28, 42.95) | -1.89 (-2.02, -1.77) |
| North Macedonia | 33.95 (24.50, 44.69) | 42.07 (33.15, 52.52) | 0.68 (0.51, 0.84) | 289.00 (247.98, 331.08) | 271.41 (233.32, 311.87) | -0.30 (-0.33, -0.26) | 975.67 (682.52, 1395.68) | 866.23 (573.34, 1229.71) | -0.49 (-0.82, -0.16) | 41.42 (28.16, 61.28) | 39.97 (25.57, 58.22) | -0.27 (-0.67, 0.13) |
| Northern Mariana Islands | 48.51 (34.65, 65.90) | 35.38 (25.84, 46.95) | -1.17 (-1.24, -1.09) | 494.26 (438.63, 550.36) | 416.23 (376.69, 457.93) | -0.61 (-0.64, -0.57) | 1260.48 (765.38, 1962.13) | 718.14 (456.02, 1071.98) | -2.02 (-2.20, -1.85) | 60.47 (34.88, 96.23) | 34.65 (21.29, 53.41) | -2.10 (-2.30, -1.90) |
| Norway | 30.29 (20.24, 43.90) | 20.30 (13.90, 28.45) | -1.07 (-1.31, -0.84) | 459.45 (376.74, 552.55) | 390.47 (328.78, 461.89) | -0.55 (-0.64, -0.46) | 599.24 (543.68, 651.78) | 223.53 (193.77, 251.82) | -3.02 (-3.26, -2.78) | 25.58 (22.84, 27.82) | 9.56 (8.03, 10.69) | -3.01 (-3.30, -2.72) |
| Oman | 31.80 (20.08, 55.50) | 43.37 (32.33, 56.62) | 1.05 (0.93, 1.17) | 362.72 (308.27, 425.24) | 322.47 (281.13, 367.77) | -0.50 (-0.54, -0.45) | 411.55 (224.20, 731.75) | 186.50 (112.36, 293.80) | -2.56 (-2.67, -2.46) | 15.87 (7.64, 30.55) | 6.89 (3.30, 12.05) | -2.60 (-2.78, -2.42) |
| Pakistan | 35.29 (23.91, 50.05) | 27.63 (19.13, 38.15) | -1.07 (-1.23, -0.91) | 319.00 (249.19, 403.14) | 283.00 (226.77, 348.87) | -0.42 (-0.44, -0.40) | 633.71 (322.31, 1181.98) | 567.60 (328.42, 940.98) | -0.77 (-1.00, -0.55) | 25.52 (11.81, 51.81) | 22.89 (12.39, 40.10) | -0.77 (-1.00, -0.53) |
| Palau | 57.71 (41.90, 77.65) | 40.08 (29.69, 52.41) | -1.36 (-1.44, -1.29) | 595.97 (528.47, 672.42) | 521.57 (470.84, 573.19) | -0.49 (-0.51, -0.47) | 810.39 (493.76, 1309.86) | 605.76 (378.63, 921.46) | -0.77 (-0.89, -0.65) | 35.68 (19.40, 61.62) | 28.97 (16.67, 46.61) | -0.40 (-0.56, -0.23) |
| Palestine | 16.87 (11.63, 23.39) | 11.62 (8.19, 15.98) | -1.46 (-1.56, -1.36) | 232.93 (206.50, 263.32) | 203.01 (182.17, 228.18) | -0.48 (-0.52, -0.45) | 187.42 (111.57, 312.16) | 113.82 (75.52, 163.23) | -1.57 (-1.76, -1.39) | 7.57 (3.97, 13.48) | 4.29 (2.52, 6.63) | -1.77 (-2.01, -1.53) |
| Panama | 46.28 (32.60, 62.96) | 37.49 (27.25, 50.51) | -0.81 (-0.88, -0.74) | 660.02 (603.02, 717.05) | 598.16 (553.03, 644.59) | -0.36 (-0.38, -0.34) | 584.70 (472.19, 715.77) | 500.19 (381.87, 624.16) | -0.53 (-0.77, -0.29) | 23.92 (18.74, 29.98) | 20.57 (15.12, 26.17) | -0.54 (-0.79, -0.28) |
| Papua New Guinea | 44.38 (31.00, 60.87) | 35.28 (25.15, 47.54) | -0.84 (-0.90, -0.78) | 375.11 (325.82, 426.64) | 332.24 (293.79, 370.62) | -0.47 (-0.50, -0.44) | 1390.34 (721.09, 2465.79) | 973.49 (526.84, 1707.30) | -1.25 (-1.28, -1.21) | 65.38 (32.20, 119.87) | 45.72 (23.62, 82.90) | -1.24 (-1.28, -1.21) |
| Paraguay | 32.97 (23.95, 43.57) | 32.02 (23.30, 42.23) | 0.13 (0.04, 0.21) | 477.83 (427.99, 532.50) | 426.29 (385.22, 471.49) | -0.46 (-0.49, -0.44) | 708.68 (477.11, 1036.57) | 643.06 (413.03, 957.98) | 0.14 (-0.07, 0.35) | 28.17 (18.34, 42.19) | 25.67 (15.73, 39.08) | 0.26 (0.03, 0.48) |
| Peru | 40.31 (28.22, 55.55) | 27.09 (19.28, 36.85) | -1.33 (-1.47, -1.19) | 662.98 (602.70, 726.83) | 509.29 (468.97, 549.12) | -0.99 (-1.04, -0.94) | 658.47 (403.07, 1042.87) | 459.34 (286.88, 708.25) | -1.59 (-1.97, -1.21) | 26.36 (15.13, 43.97) | 18.27 (10.52, 29.50) | -1.59 (-2.01, -1.17) |
| Philippines | 19.52 (12.94, 28.42) | 23.93 (16.44, 33.59) | 1.04 (0.80, 1.29) | 281.73 (235.13, 338.89) | 354.32 (295.04, 424.24) | 0.90 (0.81, 0.99) | 372.49 (301.24, 520.29) | 437.35 (336.50, 572.35) | 0.85 (0.71, 1.00) | 15.86 (12.33, 24.60) | 17.17 (12.83, 24.49) | 0.62 (0.48, 0.75) |
| Poland | 21.16 (14.27, 30.28) | 12.81 (8.89, 17.83) | -2.14 (-2.33, -1.95) | 254.79 (207.47, 309.64) | 171.57 (141.83, 205.62) | -1.47 (-1.59, -1.34) | 411.51 (383.86, 440.29) | 232.24 (204.50, 261.09) | -2.08 (-2.51, -1.65) | 15.32 (14.32, 16.34) | 9.08 (7.85, 10.25) | -1.97 (-2.42, -1.52) |
| Portugal | 25.26 (18.17, 33.61) | 15.57 (10.43, 20.61) | -1.87 (-2.02, -1.71) | 320.01 (284.97, 357.43) | 210.00 (188.20, 233.35) | -1.59 (-1.68, -1.51) | 478.16 (401.34, 565.48) | 260.82 (211.92, 311.29) | -1.62 (-1.83, -1.41) | 21.05 (17.49, 24.91) | 12.19 (9.59, 14.75) | -1.32 (-1.57, -1.07) |
| Puerto Rico | 27.39 (19.03, 37.62) | 20.33 (14.06, 28.50) | -1.14 (-1.25, -1.02) | 515.59 (472.29, 558.28) | 431.26 (399.54, 462.66) | -0.66 (-0.71, -0.61) | 332.76 (271.02, 403.12) | 218.44 (171.70, 272.61) | -1.89 (-2.17, -1.61) | 13.25 (10.55, 16.33) | 8.36 (6.31, 10.69) | -2.05 (-2.35, -1.75) |
| Qatar | 23.79 (16.58, 32.25) | 25.79 (19.25, 33.84) | 0.70 (0.53, 0.88) | 398.45 (356.39, 444.16) | 340.86 (306.75, 376.12) | -0.56 (-0.59, -0.53) | 646.59 (370.60, 1125.23) | 271.50 (167.46, 419.34) | -2.94 (-3.51, -2.38) | 29.60 (16.03, 53.95) | 12.06 (6.68, 19.96) | -3.08 (-3.74, -2.42) |
| Republic of Korea | 80.27 (56.93, 110.08) | 40.76 (29.10, 55.16) | -2.36 (-2.71, -2.01) | 779.33 (686.39, 883.06) | 573.29 (533.04, 613.98) | -0.91 (-1.13, -0.68) | 1638.28 (1056.14, 2452.23) | 382.88 (282.18, 506.41) | -4.76 (-4.95, -4.58) | 73.98 (45.33, 115.22) | 15.41 (10.23, 21.92) | -5.11 (-5.27, -4.94) |
| Republic of Moldova | 15.90 (11.19, 21.91) | 11.56 (8.01, 15.85) | -1.25 (-1.34, -1.15) | 149.51 (126.87, 174.28) | 131.08 (112.26, 150.89) | -0.42 (-0.46, -0.37) | 215.15 (173.41, 264.25) | 174.50 (139.54, 214.41) | -1.28 (-2.03, -0.52) | 8.89 (7.09, 10.99) | 7.00 (5.58, 8.67) | -1.39 (-2.19, -0.58) |
| Romania | 30.01 (23.14, 38.30) | 28.85 (23.19, 35.41) | -0.28 (-0.34, -0.22) | 192.25 (164.80, 220.95) | 163.00 (139.96, 186.07) | -0.65 (-0.71, -0.59) | 499.18 (396.14, 647.06) | 427.26 (337.49, 528.70) | -0.69 (-1.21, -0.17) | 21.65 (16.72, 28.42) | 19.97 (15.78, 24.48) | -0.32 (-0.88, 0.24) |
| Russian Federation | 21.84 (14.76, 31.18) | 24.00 (16.89, 32.77) | -0.04 (-0.56, 0.48) | 226.98 (180.24, 282.38) | 226.22 (184.75, 274.97) | -0.04 (-0.09, 0.02) | 402.99 (380.09, 426.97) | 449.99 (396.87, 497.50) | -0.52 (-1.74, 0.71) | 16.57 (15.59, 17.40) | 21.01 (18.32, 23.20) | -0.10 (-1.62, 1.43) |
| Rwanda | 27.04 (17.52, 38.71) | 18.38 (12.19, 26.34) | -1.67 (-1.83, -1.52) | 431.10 (379.18, 481.82) | 296.20 (266.31, 327.29) | -1.34 (-1.39, -1.28) | 502.42 (224.34, 1276.78) | 219.60 (102.00, 524.02) | -3.45 (-3.78, -3.12) | 19.07 (7.12, 54.65) | 8.12 (2.92, 22.50) | -3.62 (-3.98, -3.25) |
| Saint Kitts and Nevis | 40.74 (29.82, 54.40) | 27.42 (20.19, 36.22) | -1.45 (-1.56, -1.33) | 576.96 (520.36, 634.05) | 429.51 (391.90, 468.80) | -1.11 (-1.17, -1.05) | 784.11 (577.97, 1026.57) | 429.47 (337.80, 541.02) | -2.29 (-2.56, -2.02) | 32.05 (23.04, 42.60) | 18.32 (14.17, 23.21) | -2.13 (-2.42, -1.84) |
| Saint Lucia | 39.21 (28.18, 53.54) | 26.06 (19.03, 35.08) | -1.59 (-1.74, -1.43) | 561.20 (505.86, 617.59) | 471.44 (431.46, 509.73) | -0.61 (-0.64, -0.58) | 759.85 (598.98, 941.74) | 392.66 (305.42, 495.24) | -2.81 (-3.15, -2.47) | 31.17 (24.26, 39.00) | 16.00 (12.17, 20.45) | -2.98 (-3.35, -2.61) |
| Saint Vincent and the Grenadines | 27.83 (19.31, 37.75) | 20.96 (15.02, 28.31) | -0.99 (-1.13, -0.85) | 457.57 (414.37, 501.14) | 393.87 (361.00, 427.77) | -0.55 (-0.58, -0.52) | 435.19 (345.61, 538.06) | 302.97 (243.29, 378.06) | -1.28 (-1.46, -1.09) | 17.46 (13.56, 21.93) | 11.72 (9.21, 14.80) | -1.35 (-1.58, -1.12) |
| Samoa | 56.09 (39.52, 76.29) | 45.66 (33.29, 61.06) | -0.81 (-0.86, -0.76) | 569.70 (498.94, 642.60) | 560.87 (500.57, 620.13) | -0.04 (-0.06, -0.03) | 1005.36 (588.09, 1660.02) | 838.65 (500.46, 1311.82) | -0.66 (-0.81, -0.51) | 46.87 (25.40, 79.90) | 37.31 (20.73, 61.60) | -0.83 (-1.01, -0.65) |
| San Marino | 18.99 (12.76, 26.62) | 14.97 (9.90, 21.47) | -0.92 (-1.00, -0.85) | 309.43 (273.11, 344.71) | 278.97 (249.11, 308.98) | -0.34 (-0.39, -0.28) | 194.94 (123.46, 289.91) | 98.97 (63.29, 149.18) | -1.80 (-1.98, -1.62) | 7.67 (4.44, 12.27) | 3.03 (1.51, 5.21) | -2.53 (-2.82, -2.25) |
| Sao Tome and Principe | 13.74 (8.79, 20.39) | 12.33 (7.95, 17.88) | -0.50 (-0.57, -0.43) | 316.50 (284.60, 349.91) | 297.50 (272.54, 324.90) | -0.22 (-0.25, -0.20) | 276.41 (151.12, 596.48) | 273.64 (149.46, 578.51) | -0.24 (-0.43, -0.05) | 10.27 (4.84, 25.09) | 10.09 (4.71, 23.74) | -0.24 (-0.44, -0.04) |
| Saudi Arabia | 14.14 (9.52, 19.88) | 9.79 (6.69, 13.54) | -1.38 (-1.47, -1.28) | 187.57 (163.24, 216.24) | 170.25 (149.23, 194.85) | -0.27 (-0.33, -0.21) | 233.15 (135.68, 408.18) | 161.89 (96.20, 266.64) | -0.97 (-1.18, -0.76) | 9.39 (5.02, 17.30) | 6.31 (3.30, 11.00) | -1.07 (-1.32, -0.81) |
| Senegal | 12.15 (7.61, 18.17) | 10.59 (6.75, 15.68) | -0.51 (-0.56, -0.46) | 263.45 (237.60, 291.74) | 239.99 (218.52, 262.12) | -0.32 (-0.35, -0.30) | 292.80 (162.09, 624.01) | 268.25 (142.73, 593.08) | -0.14 (-0.26, -0.02) | 11.25 (5.42, 26.49) | 10.48 (4.94, 25.29) | -0.06 (-0.19, 0.07) |
| Serbia | 34.56 (26.21, 44.50) | 32.45 (26.08, 40.01) | -0.49 (-0.64, -0.34) | 269.88 (233.04, 311.08) | 189.08 (164.31, 216.12) | -1.37 (-1.47, -1.28) | 963.07 (633.42, 1390.08) | 538.62 (366.05, 777.57) | -2.59 (-2.94, -2.24) | 41.52 (26.64, 61.54) | 23.47 (15.56, 34.51) | -2.58 (-2.93, -2.23) |
| Seychelles | 24.35 (16.44, 34.54) | 17.77 (11.69, 25.16) | -1.13 (-1.23, -1.03) | 348.31 (304.51, 393.94) | 295.85 (259.43, 332.88) | -0.43 (-0.51, -0.34) | 389.71 (243.29, 614.98) | 222.48 (130.06, 360.28) | -1.37 (-1.53, -1.21) | 15.56 (8.51, 27.33) | 8.32 (3.79, 15.79) | -1.50 (-1.72, -1.27) |
| Sierra Leone | 12.17 (7.61, 18.38) | 11.26 (7.30, 16.70) | -0.26 (-0.32, -0.20) | 261.79 (235.07, 289.32) | 254.18 (232.05, 276.97) | -0.11 (-0.13, -0.09) | 278.93 (139.25, 658.85) | 297.39 (148.25, 683.24) | 0.54 (0.32, 0.76) | 10.81 (4.65, 28.10) | 11.47 (5.01, 29.28) | 0.55 (0.31, 0.79) |
| Singapore | 52.31 (37.69, 71.25) | 29.64 (19.93, 41.79) | -2.28 (-2.44, -2.11) | 759.47 (696.81, 826.85) | 545.38 (504.93, 588.63) | -1.08 (-1.14, -1.02) | 675.40 (568.88, 798.36) | 229.63 (184.53, 281.54) | -3.74 (-3.89, -3.59) | 23.60 (19.53, 28.33) | 6.61 (5.10, 8.26) | -4.49 (-4.74, -4.23) |
| Slovakia | 23.57 (17.70, 30.73) | 12.85 (9.44, 17.07) | -2.38 (-2.55, -2.22) | 236.20 (212.66, 261.36) | 171.98 (154.33, 189.57) | -0.95 (-1.00, -0.90) | 401.03 (275.46, 561.57) | 221.44 (148.63, 321.82) | -1.88 (-1.95, -1.81) | 15.76 (10.24, 22.84) | 8.75 (5.44, 13.34) | -1.84 (-1.93, -1.76) |
| Slovenia | 17.80 (12.84, 23.86) | 12.09 (8.55, 16.49) | -1.78 (-1.94, -1.61) | 219.45 (194.61, 245.52) | 171.00 (154.57, 188.40) | -0.76 (-0.79, -0.74) | 342.39 (281.23, 412.31) | 153.50 (119.45, 191.70) | -2.67 (-2.95, -2.39) | 13.42 (10.83, 16.34) | 6.29 (4.75, 7.93) | -2.49 (-2.83, -2.14) |
| Solomon Islands | 75.35 (47.26, 115.26) | 86.38 (64.34, 113.82) | 0.03 (-0.14, 0.20) | 695.54 (580.61, 815.80) | 698.86 (604.52, 796.24) | -0.02 (-0.09, 0.05) | 1569.59 (776.35, 2810.49) | 1200.81 (679.11, 2107.99) | -0.89 (-1.08, -0.71) | 74.48 (33.88, 140.09) | 55.42 (28.28, 102.27) | -1.00 (-1.15, -0.85) |
| Somalia | 21.22 (13.32, 31.85) | 20.61 (13.09, 30.90) | -0.14 (-0.17, -0.11) | 355.05 (309.99, 402.18) | 339.38 (297.56, 380.57) | -0.16 (-0.18, -0.15) | 423.36 (153.37, 1430.76) | 340.62 (111.76, 1231.93) | -0.70 (-0.79, -0.62) | 16.28 (4.55, 61.95) | 12.99 (2.92, 54.71) | -0.72 (-0.80, -0.63) |
| South Africa | 14.90 (9.75, 21.66) | 13.41 (8.90, 19.16) | -0.52 (-0.73, -0.31) | 272.59 (226.56, 326.84) | 226.23 (190.45, 268.76) | -0.69 (-0.74, -0.65) | 129.98 (101.12, 163.95) | 156.24 (123.58, 191.16) | 0.68 (0.34, 1.01) | 4.23 (3.06, 5.72) | 5.81 (4.31, 7.33) | 1.10 (0.66, 1.54) |
| South Sudan | 18.23 (11.95, 26.24) | 15.49 (10.12, 22.63) | -0.63 (-0.71, -0.54) | 299.89 (268.70, 332.93) | 269.16 (242.54, 295.82) | -0.37 (-0.40, -0.35) | 285.34 (145.24, 630.31) | 248.35 (121.31, 569.16) | -0.65 (-0.97, -0.33) | 10.82 (4.75, 26.37) | 9.33 (3.68, 24.19) | -0.70 (-1.06, -0.34) |
| Spain | 19.13 (13.36, 26.33) | 17.50 (12.65, 23.55) | -0.30 (-0.42, -0.17) | 294.03 (263.82, 326.10) | 298.05 (271.81, 325.58) | 0.06 (0.03, 0.09) | 227.86 (186.82, 271.14) | 201.80 (160.63, 244.76) | -0.19 (-0.49, 0.11) | 9.17 (7.42, 10.99) | 8.67 (6.60, 10.74) | 0.14 (-0.30, 0.59) |
| Sri Lanka | 46.55 (33.32, 62.52) | 32.42 (23.47, 43.62) | -1.35 (-1.49, -1.21) | 476.40 (423.88, 529.68) | 384.07 (348.57, 419.78) | -0.78 (-0.83, -0.74) | 362.46 (242.42, 521.43) | 230.64 (153.02, 328.05) | -1.23 (-1.35, -1.10) | 14.49 (8.45, 22.41) | 8.51 (4.70, 13.56) | -1.22 (-1.44, -1.00) |
| Sudan | 21.57 (14.23, 30.70) | 15.83 (11.13, 21.61) | -1.26 (-1.35, -1.17) | 233.65 (198.74, 273.56) | 181.41 (159.14, 205.76) | -0.93 (-0.97, -0.88) | 562.26 (189.16, 1455.96) | 353.88 (143.88, 700.19) | -1.63 (-1.69, -1.58) | 24.36 (7.23, 70.21) | 15.87 (5.80, 33.25) | -1.50 (-1.55, -1.44) |
| Suriname | 30.83 (22.11, 42.61) | 26.64 (19.09, 35.66) | -0.65 (-0.74, -0.57) | 481.13 (432.73, 528.52) | 432.54 (394.59, 472.33) | -0.37 (-0.40, -0.35) | 558.60 (371.51, 824.99) | 407.69 (254.99, 622.59) | -1.00 (-1.23, -0.78) | 22.57 (14.44, 34.30) | 15.86 (9.17, 25.19) | -1.05 (-1.28, -0.82) |
| Sweden | 27.29 (18.15, 39.60) | 19.92 (13.46, 28.22) | -1.13 (-1.25, -1.00) | 386.53 (315.94, 469.70) | 312.64 (261.15, 372.84) | -0.77 (-0.83, -0.71) | 497.13 (421.38, 575.83) | 221.57 (177.60, 268.14) | -2.54 (-2.82, -2.26) | 20.88 (17.48, 24.36) | 9.33 (7.16, 11.46) | -2.50 (-2.84, -2.15) |
| Switzerland | 23.73 (17.14, 31.51) | 16.87 (11.79, 23.38) | -1.03 (-1.37, -0.70) | 275.81 (246.10, 306.21) | 264.04 (240.07, 287.27) | -0.18 (-0.30, -0.07) | 335.93 (271.81, 408.03) | 175.28 (138.08, 213.59) | -1.89 (-2.04, -1.74) | 14.52 (11.46, 17.99) | 7.67 (5.76, 9.44) | -1.77 (-1.93, -1.61) |
| Syrian Arab Republic | 18.46 (11.82, 27.07) | 12.74 (8.95, 17.46) | -1.61 (-1.78, -1.45) | 275.37 (243.53, 309.88) | 218.62 (196.60, 243.44) | -0.85 (-0.90, -0.79) | 334.70 (188.61, 700.58) | 214.78 (128.84, 340.41) | -2.04 (-2.38, -1.71) | 14.49 (7.42, 33.68) | 9.40 (4.99, 15.70) | -2.07 (-2.44, -1.70) |
| Taiwan, China | 30.03 (21.38, 40.69) | 23.12 (16.26, 31.85) | -1.04 (-1.15, -0.93) | 355.00 (306.17, 405.11) | 314.07 (270.20, 355.96) | -0.45 (-0.47, -0.42) | 471.41 (397.37, 554.12) | 239.99 (191.07, 290.99) | -2.79 (-3.05, -2.54) | 18.89 (15.66, 22.55) | 9.07 (7.03, 11.21) | -3.23 (-3.57, -2.88) |
| Tajikistan | 23.61 (16.49, 32.42) | 31.90 (23.86, 41.61) | 1.21 (1.00, 1.42) | 233.65 (200.78, 266.15) | 227.85 (198.63, 258.76) | -0.16 (-0.19, -0.12) | 409.11 (246.60, 675.19) | 374.14 (239.43, 552.21) | -0.70 (-1.19, -0.20) | 17.81 (9.80, 32.85) | 16.79 (10.06, 25.80) | -0.64 (-1.21, -0.07) |
| Thailand | 62.12 (45.26, 83.88) | 35.62 (25.97, 47.64) | -2.21 (-2.40, -2.01) | 550.95 (489.25, 615.87) | 402.46 (364.47, 440.88) | -1.12 (-1.17, -1.07) | 1329.46 (785.96, 2133.27) | 625.06 (404.67, 920.11) | -2.97 (-3.23, -2.72) | 65.69 (36.43, 108.74) | 29.92 (17.99, 45.86) | -3.09 (-3.39, -2.79) |
| Timor-Leste | 32.67 (22.40, 45.10) | 27.14 (19.34, 36.64) | -0.76 (-0.82, -0.69) | 331.75 (288.89, 377.31) | 312.51 (275.73, 349.69) | -0.22 (-0.24, -0.19) | 918.06 (435.99, 1745.15) | 680.66 (351.75, 1254.08) | -0.93 (-1.09, -0.77) | 39.80 (17.28, 81.20) | 29.70 (13.87, 59.58) | -0.90 (-1.06, -0.74) |
| Togo | 12.73 (8.11, 19.09) | 11.30 (7.23, 16.84) | -0.47 (-0.50, -0.44) | 264.15 (236.95, 292.50) | 241.26 (219.12, 263.06) | -0.33 (-0.34, -0.31) | 311.30 (152.77, 702.85) | 281.53 (139.07, 679.48) | -0.21 (-0.29, -0.13) | 12.04 (5.07, 30.25) | 11.10 (4.66, 29.36) | -0.13 (-0.23, -0.03) |
| Tokelau | 63.51 (44.35, 86.80) | 39.87 (28.62, 53.54) | -1.90 (-2.04, -1.75) | 611.88 (534.38, 693.04) | 514.60 (457.31, 571.01) | -0.58 (-0.60, -0.55) | 1413.45 (720.80, 2576.67) | 712.29 (424.05, 1110.23) | -2.32 (-2.37, -2.28) | 64.76 (30.10, 122.31) | 30.91 (16.68, 50.68) | -2.50 (-2.55, -2.45) |
| Tonga | 41.44 (28.30, 58.50) | 30.90 (21.66, 41.98) | -1.13 (-1.22, -1.04) | 437.22 (381.24, 492.35) | 371.77 (333.80, 410.37) | -0.59 (-0.62, -0.56) | 570.20 (334.30, 941.12) | 412.11 (247.44, 659.99) | -0.87 (-0.98, -0.76) | 24.99 (13.15, 44.80) | 18.32 (9.83, 31.37) | -0.72 (-0.86, -0.59) |
| Trinidad and Tobago | 34.29 (24.63, 46.06) | 27.12 (19.61, 36.13) | -0.87 (-0.92, -0.82) | 495.81 (446.98, 546.40) | 415.86 (378.93, 452.89) | -0.65 (-0.69, -0.61) | 652.42 (528.04, 794.37) | 407.87 (305.35, 535.44) | -1.91 (-2.09, -1.72) | 26.11 (20.67, 32.22) | 17.03 (12.41, 22.62) | -1.73 (-1.91, -1.55) |
| Tunisia | 21.19 (14.06, 30.33) | 14.85 (10.22, 20.56) | -1.41 (-1.51, -1.32) | 269.37 (234.81, 305.15) | 222.29 (198.75, 247.14) | -0.69 (-0.73, -0.64) | 345.54 (166.01, 796.51) | 214.15 (110.68, 397.61) | -1.57 (-1.63, -1.52) | 15.26 (6.38, 38.26) | 9.50 (4.17, 19.05) | -1.49 (-1.57, -1.41) |
| Türkiye | 28.11 (19.25, 38.78) | 17.50 (12.56, 23.29) | -1.78 (-1.88, -1.67) | 360.80 (320.12, 403.19) | 257.84 (235.26, 283.42) | -1.24 (-1.30, -1.17) | 712.33 (376.55, 1476.03) | 322.73 (208.68, 484.05) | -2.68 (-2.97, -2.39) | 31.32 (15.31, 68.55) | 14.75 (9.02, 22.88) | -2.32 (-2.70, -1.93) |
| Turkmenistan | 24.01 (17.27, 32.61) | 28.12 (20.71, 36.70) | 0.84 (0.72, 0.96) | 223.67 (194.89, 255.81) | 246.60 (217.39, 277.53) | 0.37 (0.34, 0.40) | 472.53 (352.11, 617.13) | 638.07 (398.23, 897.47) | 0.95 (0.61, 1.30) | 18.63 (13.60, 25.24) | 26.59 (15.92, 38.76) | 1.10 (0.71, 1.49) |
| Tuvalu | 64.41 (45.33, 88.25) | 48.83 (35.38, 65.47) | -1.13 (-1.21, -1.05) | 590.89 (509.43, 673.86) | 519.54 (458.16, 580.32) | -0.46 (-0.49, -0.43) | 1661.14 (783.31, 3032.18) | 941.60 (506.29, 1563.54) | -1.85 (-1.96, -1.73) | 76.40 (33.33, 144.69) | 42.30 (20.60, 73.40) | -1.92 (-2.04, -1.81) |
| Uganda | 19.61 (12.30, 29.16) | 17.08 (11.21, 25.06) | -0.57 (-0.63, -0.51) | 296.73 (262.51, 334.09) | 266.28 (238.62, 294.62) | -0.40 (-0.42, -0.38) | 236.78 (106.49, 661.93) | 170.49 (87.40, 400.34) | -1.54 (-1.75, -1.32) | 8.89 (3.07, 29.36) | 6.10 (2.42, 16.84) | -1.75 (-1.99, -1.51) |
| Ukraine | 28.12 (18.85, 40.26) | 19.88 (13.74, 27.68) | -1.32 (-1.40, -1.23) | 207.55 (165.13, 256.23) | 195.26 (159.46, 235.95) | -0.18 (-0.20, -0.16) | 566.22 (440.05, 707.33) | 350.88 (240.53, 498.86) | -1.96 (-2.16, -1.77) | 28.51 (21.74, 35.95) | 16.98 (11.35, 24.19) | -2.11 (-2.33, -1.89) |
| United Arab Emirates | 22.48 (15.46, 31.42) | 14.32 (10.30, 19.35) | -1.65 (-1.93, -1.36) | 278.25 (241.03, 317.83) | 236.14 (211.57, 262.23) | -0.60 (-0.63, -0.57) | 905.96 (449.46, 1810.95) | 897.66 (486.39, 1421.81) | 1.19 (0.72, 1.66) | 39.27 (18.45, 85.68) | 50.61 (26.75, 81.32) | 2.44 (1.83, 3.06) |
| United Kingdom | 31.06 (21.82, 43.04) | 21.97 (15.63, 29.95) | -1.36 (-1.48, -1.24) | 391.00 (326.24, 465.23) | 318.27 (271.84, 371.58) | -0.77 (-0.81, -0.72) | 640.67 (606.74, 671.99) | 313.33 (285.04, 338.26) | -2.41 (-2.59, -2.24) | 25.87 (24.30, 27.03) | 13.94 (12.29, 15.01) | -1.98 (-2.17, -1.78) |
| United Republic of Tanzania | 20.07 (12.86, 29.59) | 18.28 (12.02, 26.54) | -0.34 (-0.36, -0.32) | 314.17 (277.63, 353.52) | 285.87 (257.82, 314.82) | -0.35 (-0.37, -0.33) | 250.30 (115.34, 610.60) | 208.52 (107.78, 480.77) | -0.56 (-0.63, -0.48) | 9.13 (3.24, 25.44) | 7.67 (3.06, 20.20) | -0.44 (-0.57, -0.31) |
| United States of America | 21.73 (14.41, 31.53) | 19.17 (13.17, 26.76) | -0.59 (-0.77, -0.40) | 328.81 (271.62, 396.19) | 357.23 (309.72, 410.78) | 0.15 (0.07, 0.22) | 418.63 (389.34, 446.83) | 339.04 (306.72, 367.27) | -0.82 (-0.97, -0.67) | 16.05 (14.66, 17.11) | 14.03 (12.23, 15.23) | -0.50 (-0.69, -0.31) |
| United States Virgin Islands | 36.50 (25.64, 51.07) | 26.37 (18.75, 35.61) | -1.22 (-1.30, -1.15) | 572.56 (518.16, 627.75) | 464.50 (425.94, 502.50) | -0.76 (-0.80, -0.72) | 597.74 (368.46, 917.71) | 235.56 (146.84, 357.03) | -2.91 (-3.11, -2.72) | 24.51 (14.17, 39.03) | 8.55 (4.76, 14.01) | -3.23 (-3.45, -3.01) |
| Uruguay | 49.47 (36.36, 65.55) | 42.57 (31.40, 56.23) | -0.51 (-0.64, -0.38) | 604.08 (514.56, 697.89) | 535.16 (459.06, 624.23) | -0.09 (-0.28, 0.11) | 1018.43 (836.27, 1230.34) | 700.60 (581.52, 824.81) | -1.31 (-1.47, -1.15) | 40.16 (32.29, 49.12) | 29.08 (23.53, 34.83) | -1.16 (-1.34, -0.98) |
| Uzbekistan | 14.68 (9.57, 22.06) | 16.71 (12.55, 21.90) | 0.72 (0.55, 0.89) | 168.05 (145.70, 193.12) | 168.05 (150.28, 186.35) | -0.05 (-0.10, -0.01) | 159.24 (118.72, 206.73) | 354.97 (284.28, 439.16) | 2.08 (1.20, 2.97) | 5.67 (3.88, 7.97) | 16.26 (12.82, 20.24) | 2.78 (1.65, 3.92) |
| Vanuatu | 56.60 (39.83, 76.86) | 48.10 (34.94, 64.31) | -0.66 (-0.73, -0.60) | 552.89 (480.61, 629.09) | 540.41 (479.10, 603.29) | -0.09 (-0.11, -0.07) | 1152.25 (552.90, 2005.40) | 845.84 (418.12, 1441.15) | -1.16 (-1.30, -1.03) | 52.74 (23.15, 95.24) | 37.30 (16.33, 66.97) | -1.28 (-1.39, -1.17) |
| Venezuela (Bolivarian Republic of) | 38.84 (27.93, 52.27) | 34.40 (25.08, 45.68) | -0.47 (-0.53, -0.41) | 709.19 (650.32, 769.91) | 638.72 (592.70, 687.86) | -0.36 (-0.39, -0.33) | 552.76 (447.98, 674.18) | 574.03 (408.93, 767.77) | -0.17 (-0.33, -0.01) | 20.70 (16.32, 25.86) | 22.98 (15.65, 31.50) | 0.09 (-0.07, 0.26) |
| Viet Nam | 35.58 (24.10, 50.37) | 27.15 (19.29, 37.21) | -1.10 (-1.19, -1.02) | 391.58 (343.31, 442.21) | 335.34 (301.60, 370.59) | -0.57 (-0.61, -0.52) | 712.12 (393.31, 1428.11) | 446.11 (253.92, 835.75) | -1.30 (-1.40, -1.20) | 32.43 (16.14, 70.36) | 20.02 (9.86, 42.53) | -1.35 (-1.44, -1.26) |
| Yemen | 23.65 (15.73, 33.41) | 20.16 (14.30, 27.40) | -0.73 (-0.81, -0.65) | 250.28 (212.19, 291.36) | 215.34 (185.83, 247.13) | -0.55 (-0.59, -0.51) | 597.95 (213.30, 1502.76) | 469.95 (198.51, 1062.88) | -1.11 (-1.26, -0.96) | 25.98 (8.26, 70.64) | 21.42 (8.12, 51.40) | -0.94 (-1.10, -0.78) |
| Zambia | 21.07 (13.82, 30.57) | 18.44 (12.48, 26.21) | -0.59 (-0.64, -0.54) | 331.49 (294.64, 368.40) | 292.05 (263.15, 320.05) | -0.47 (-0.49, -0.45) | 374.83 (181.28, 869.27) | 310.83 (159.17, 699.26) | -0.91 (-1.04, -0.77) | 14.66 (5.84, 37.46) | 12.15 (5.44, 29.71) | -0.93 (-1.08, -0.78) |
| Zimbabwe | 18.28 (12.12, 26.15) | 21.21 (14.62, 29.37) | 0.89 (0.74, 1.05) | 284.72 (253.75, 316.70) | 351.70 (317.16, 385.31) | 0.75 (0.71, 0.79) | 355.44 (216.60, 606.59) | 542.84 (303.47, 919.34) | 2.54 (1.78, 3.31) | 15.09 (8.52, 27.36) | 22.82 (11.96, 40.46) | 2.56 (1.78, 3.35) |

Note. Rates are expressed per 100,000 women and were age-standardized to the WHO World Standard Population (WHO 2000–2025), renormalized within ages 55 years and older. Abbreviations: ASDR, age-standardized DALY rate; ASIR, age-standardized incidence rate; ASMR, age-standardized mortality rate; ASPR, age-standardized prevalence rate; CI, confidence interval; EAPC, estimated annual percentage change; UI, uncertainty interval.
