# Supplementary material for: Global burden and trends of subarachnoid hemorrhage in women aged 55 years and older: A repeated cross-sectional analysis of the GBD 2021 study
Source: Medicine (Baltimore). 2026 Nov 6;105(45):e51457. doi: 10.1097/MD.0000000000051457 (PMC13747508; doi:10.1097/MD.0000000000051457)
Supplement: Supplementary file 4 [file medi-105-e51457-s008.docx]

Table S4. Spearman correlation between age-standardized rates of subarachnoid hemorrhage (SAH) and the Sociodemographic Index (SDI) among postmenopausal women, 1990–2021 (21 GBD regions and the global level; 32 years; n = 704)

| Measure | rho | P value | Method |
| --- | --- | --- | --- |
| Deaths Rate | 0.010 | 0.792 | Spearman's rank correlation rho |
| DALYs Rate | -0.021 | 0.572 | Spearman's rank correlation rho |
| Prevalence Rate | 0.066 | 0.082 | Spearman's rank correlation rho |
| Incidence Rate | 0.064 | 0.091 | Spearman's rank correlation rho |

Note. Abbreviations: DALYs, disability-adjusted life-years; rho, Spearman rank correlation coefficient.
