# Supplementary material for: Global burden and trends of subarachnoid hemorrhage in women aged 55 years and older: A repeated cross-sectional analysis of the GBD 2021 study
Source: Medicine (Baltimore). 2026 Nov 6;105(45):e51457. doi: 10.1097/MD.0000000000051457 (PMC13747508; doi:10.1097/MD.0000000000051457)
Supplement: Supplementary file 5 [file medi-105-e51457-s010.docx]

# Table S5. Decomposition of SAH burden among postmenopausal women, 1990–2021

Decomposition of changes in incidence, prevalence, DALYs, and deaths of subarachnoid hemorrhage (SAH) among postmenopausal women from 1990 to 2021, stratified by Sociodemographic Index (SDI) regions. The decomposition analysis quantifies the contributions of three components: (1) aging effect, (2) population growth effect, and (3) epidemiological change. Results are presented as absolute differences and percentages across global and five SDI regions.

## (A) Incidence decomposition

| **Location** | **Overall difference** | **Aging effect** | **Population growth effect** | **Epidemiological change** | **Aging effect (%)** | **Population growth effect (%)** | **Epidemiological change (%)** |
| --- | --- | --- | --- | --- | --- | --- | --- |
| Global | 64551.96 | 5111.96 | 125596.54 | -66156.54 | 7.92% | 194.57% | -102.49% |
| High SDI | 15809.40 | 1615.32 | 24680.34 | -10486.26 | 10.22% | 156.11% | -66.33% |
| High-middle SDI | 8280.38 | 2244.06 | 24361.31 | -18324.99 | 27.10% | 294.21% | -221.31% |
| Middle SDI | 23263.75 | 3052.86 | 54480.67 | -34269.78 | 13.12% | 234.19% | -147.31% |
| Low-middle SDI | 13735.75 | 520.67 | 19306.67 | -6091.60 | 3.79% | 140.56% | -44.35% |
| Low SDI | 3404.93 | 53.20 | 4716.26 | -1364.53 | 1.56% | 138.51% | -40.08% |

## (B) Prevalence decomposition

| **Location** | **Overall difference** | **Aging effect** | **Population growth effect** | **Epidemiological change** | **Aging effect (%)** | **Population growth effect (%)** | **Epidemiological change (%)** |
| --- | --- | --- | --- | --- | --- | --- | --- |
| Global | 1128683.27 | 7943.02 | 1408317.55 | -287577.30 | 0.70% | 124.78% | -25.48% |
| High SDI | 395735.06 | 6089.96 | 365769.88 | 23875.22 | 1.54% | 92.43% | 6.03% |
| High-middle SDI | 148438.13 | -2607.25 | 223674.93 | -72629.55 | -1.76% | 150.69% | -48.93% |
| Middle SDI | 366307.78 | 921.43 | 517543.83 | -152157.48 | 0.25% | 141.29% | -41.54% |
| Low-middle SDI | 169278.39 | 13.97 | 207267.68 | -38003.27 | 0.01% | 122.44% | -22.45% |
| Low SDI | 48228.04 | 191.63 | 61251.84 | -13215.44 | 0.40% | 127.00% | -27.40% |

## (C) DALYs decomposition

| **Location** | **Overall difference** | **Aging effect** | **Population growth effect** | **Epidemiological change** | **Aging effect (%)** | **Population growth effect (%)** | **Epidemiological change (%)** |
| --- | --- | --- | --- | --- | --- | --- | --- |
| Global | -104003.34 | 42036.25 | 2785210.79 | -2931250.38 | -40.42% | -2678.00% | 2818.42% |
| High SDI | 39420.05 | 13665.59 | 365752.60 | -339998.15 | 34.67% | 927.83% | -862.50% |
| High-middle SDI | -151952.99 | 21804.47 | 498862.69 | -672620.15 | -14.35% | -328.30% | 442.65% |
| Middle SDI | -334473.18 | 45321.86 | 1703625.50 | -2083420.55 | -13.55% | -509.35% | 622.90% |
| Low-middle SDI | 278528.77 | 762.26 | 415948.65 | -138182.14 | 0.27% | 149.34% | -49.61% |
| Low SDI | 64032.43 | -99.91 | 92282.02 | -28149.68 | -0.16% | 144.12% | -43.96% |

## (D) Deaths decomposition

| **Location** | **Overall difference** | **Aging effect** | **Population growth effect** | **Epidemiological change** | **Aging effect (%)** | **Population growth effect (%)** | **Epidemiological change (%)** |
| --- | --- | --- | --- | --- | --- | --- | --- |
| Global | -3239.23 | 7921.73 | 125934.47 | -137095.43 | -244.56% | -3887.79% | 4232.35% |
| High SDI | 4143.85 | 2537.67 | 16601.38 | -14995.20 | 61.24% | 400.63% | -361.87% |
| High-middle SDI | -5320.01 | 3257.72 | 23678.26 | -32255.99 | -61.24% | -445.08% | 606.31% |
| Middle SDI | -16191.95 | 6251.58 | 79392.99 | -101836.51 | -38.61% | -490.32% | 628.93% |
| Low-middle SDI | 11611.61 | 681.33 | 16480.33 | -5550.05 | 5.87% | 141.93% | -47.80% |
| Low SDI | 2488.44 | 66.91 | 3446.85 | -1025.32 | 2.69% | 138.51% | -41.20% |

Note. (A) Incidence; (B) Prevalence; (C) DALYs; (D) Deaths. Bars show the decomposition of the overall difference into aging, population growth, and epidemiological change; the percentage columns express each component relative to the overall difference. Abbreviations: SDI, Sociodemographic Index.
