# Supplementary material for: Global burden and trends of subarachnoid hemorrhage in women aged 55 years and older: A repeated cross-sectional analysis of the GBD 2021 study
Source: Medicine (Baltimore). 2026 Nov 6;105(45):e51457. doi: 10.1097/MD.0000000000051457 (PMC13747508; doi:10.1097/MD.0000000000051457)
Supplement: Supplementary file 6 [file medi-105-e51457-s012.docx]

# Table S6. Inequality in SAH burden among postmenopausal women, 1990 vs. 2021

Concentration Index (CI) and Slope Index of Inequality (SII) for incidence, prevalence, DALYs, and deaths of subarachnoid hemorrhage (SAH) among postmenopausal women in 1990 and 2021, computed on the age-standardized rates of 203 countries weighted by the female population aged 55 years and older. The CI reflects relative inequality: negative values indicate a higher burden in low-SDI countries and positive values a higher burden in high-SDI countries. The SII reflects absolute inequality across the SDI distribution and is expressed in the same units as the rate.

| **Indicator** | **Year** | **Concentration Index (CI)** | **Slope Index of Inequality (SII)** |
| --- | --- | --- | --- |
| Incidence | 1990 | -0.028 | -6.14 |
| Incidence | 2021 | 0.043 | 6.05 |
| Prevalence | 1990 | 0.092 | 194.91 |
| Prevalence | 2021 | 0.110 | 200.27 |
| DALYs | 1990 | -0.142 | -796.00 |
| DALYs | 2021 | -0.053 | -126.78 |
| Deaths | 1990 | -0.164 | -42.54 |
| Deaths | 2021 | -0.049 | -5.05 |

Note. Abbreviations: CI, concentration index (not confidence interval); DALYs, disability-adjusted life-years; SII, slope index of inequality.
