# Supplementary material for: Global burden and trends of subarachnoid hemorrhage in women aged 55 years and older: A repeated cross-sectional analysis of the GBD 2021 study
Source: Medicine (Baltimore). 2026 Nov 6;105(45):e51457. doi: 10.1097/MD.0000000000051457 (PMC13747508; doi:10.1097/MD.0000000000051457)
Supplement: Supplementary file 7 [file medi-105-e51457-s013.docx]

Table S7. Sensitivity of the global trends in the burden of subarachnoid hemorrhage to the age cut-point used to define the postmenopausal population, 1990–2021

| **Age cut-point** | **Rate in 1990 (95% UI)** | **Rate in 2021 (95% UI)** | **EAPC (95% CI)** | **Trend** |
| --- | --- | --- | --- | --- |
| **A. Incidence** | | | | |
| 50+ | 31.73 (22.12, 43.96) | 21.21 (15.23, 28.73) | -1.65 (-1.78, -1.52) | declining |
| 55+ | 34.39 (24.10, 47.71) | 22.99 (16.61, 31.02) | -1.65 (-1.77, -1.52) | declining |
| 60+ | 37.97 (26.87, 52.29) | 25.57 (18.78, 34.09) | -1.65 (-1.78, -1.52) | declining |
| **B. Prevalence** | | | | |
| 50+ | 329.80 (280.91, 385.80) | 276.58 (242.61, 315.55) | -0.63 (-0.65, -0.60) | declining |
| 55+ | 353.63 (299.81, 413.99) | 301.73 (263.73, 345.08) | -0.56 (-0.58, -0.53) | declining |
| 60+ | 371.11 (312.58, 437.18) | 324.84 (282.59, 372.76) | -0.46 (-0.48, -0.44) | declining |
| **C. Deaths** | | | | |
| 50+ | 33.22 (22.26, 41.81) | 13.95 (11.92, 16.52) | -3.30 (-3.56, -3.03) | declining |
| 55+ | 39.86 (26.35, 50.32) | 16.78 (14.28, 19.85) | -3.31 (-3.58, -3.04) | declining |
| 60+ | 47.36 (30.55, 60.02) | 20.00 (16.90, 23.63) | -3.31 (-3.58, -3.04) | declining |
| **D. DALYs** | | | | |
| 50+ | 808.00 (574.13, 996.54) | 351.58 (309.08, 407.54) | -3.12 (-3.36, -2.88) | declining |
| 55+ | 898.63 (627.81, 1116.06) | 390.85 (342.53, 450.60) | -3.15 (-3.40, -2.89) | declining |
| 60+ | 965.16 (653.60, 1206.31) | 419.33 (365.50, 482.22) | -3.17 (-3.43, -2.91) | declining |

Note. Rates are per 100,000 women and were age-standardized to the WHO World Standard Population (WHO 2000–2025), renormalized within each age range, so that only the age range entering the standardization varies across columns. Across the 21 GBD regions, the five SDI regions and the global level, the direction of the EAPC agreed at all three cut-points in 104 of 108 location-outcome combinations; in the four discordant combinations the absolute EAPC did not exceed 0.51%. Abbreviations: CI, confidence interval; EAPC, estimated annual percentage change (obtained from a log-linear regression fitted to all annual values from 1990 to 2021); SDI, Sociodemographic Index; UI, uncertainty interval.
