# Supplementary material for: Global burden and trends of subarachnoid hemorrhage in women aged 55 years and older: A repeated cross-sectional analysis of the GBD 2021 study
Source: Medicine (Baltimore). 2026 Nov 6;105(45):e51457. doi: 10.1097/MD.0000000000051457 (PMC13747508; doi:10.1097/MD.0000000000051457)
Supplement: Supplementary file 8 [file medi-105-e51457-s015.docx]

Table S8. Association between GBD 2021 risk-factor exposure and the burden of subarachnoid hemorrhage among women aged 55 years and older, 1990–2021

| **Outcome** | **Exposure** | **Analysis set** | **n** | **Unadjusted** | | **Adjusted for SDI and year** | |
| --- | --- | --- | --- | --- | --- | --- | --- |
|  |  |  |  | **rho (95% CI)** | **P** | **rho (95% CI)** | **P** |
| **A. Cross-sectional association, Global and 21 GBD regions (1990–2021)** | | | | | | | |
| Incidence | Smoking | Pooled, unadjusted | 704 | 0.225 (0.153, 0.294) | < 0.001 | 0.147 (0.074, 0.219) | < 0.001 |
| Incidence | High systolic blood pressure | Pooled, unadjusted | 704 | -0.447 (-0.504, -0.386) | < 0.001 | -0.504 (-0.558, -0.447) | < 0.001 |
| Prevalence | Smoking | Pooled, unadjusted | 704 | 0.349 (0.282, 0.412) | < 0.001 | 0.340 (0.273, 0.403) | < 0.001 |
| Prevalence | High systolic blood pressure | Pooled, unadjusted | 704 | -0.559 (-0.608, -0.506) | < 0.001 | -0.603 (-0.648, -0.554) | < 0.001 |
| Deaths | Smoking | Pooled, unadjusted | 704 | 0.182 (0.109, 0.252) | < 0.001 | 0.129 (0.056, 0.201) | < 0.001 |
| Deaths | High systolic blood pressure | Pooled, unadjusted | 704 | -0.334 (-0.398, -0.266) | < 0.001 | -0.375 (-0.437, -0.310) | < 0.001 |
| DALYs | Smoking | Pooled, unadjusted | 704 | 0.213 (0.141, 0.283) | < 0.001 | 0.184 (0.112, 0.254) | < 0.001 |
| DALYs | High systolic blood pressure | Pooled, unadjusted | 704 | -0.377 (-0.439, -0.312) | < 0.001 | -0.422 (-0.481, -0.359) | < 0.001 |
| **B. Cross-sectional association, 204 countries and territories (1990–2021)** | | | | | | | |
| Incidence | Smoking | Pooled, unadjusted | 6496 | 0.321 (0.299, 0.342) | < 0.001 | 0.287 (0.264, 0.309) | < 0.001 |
| Incidence | High systolic blood pressure | Pooled, unadjusted | 6496 | -0.306 (-0.328, -0.283) | < 0.001 | -0.319 (-0.341, -0.297) | < 0.001 |
| Prevalence | Smoking | Pooled, unadjusted | 6496 | 0.226 (0.203, 0.249) | < 0.001 | 0.221 (0.198, 0.244) | < 0.001 |
| Prevalence | High systolic blood pressure | Pooled, unadjusted | 6496 | -0.415 (-0.435, -0.395) | < 0.001 | -0.422 (-0.442, -0.402) | < 0.001 |
| Deaths | Smoking | Pooled, unadjusted | 6496 | 0.217 (0.194, 0.240) | < 0.001 | 0.247 (0.224, 0.269) | < 0.001 |
| Deaths | High systolic blood pressure | Pooled, unadjusted | 6496 | -0.195 (-0.218, -0.171) | < 0.001 | -0.192 (-0.215, -0.169) | < 0.001 |
| DALYs | Smoking | Pooled, unadjusted | 6496 | 0.201 (0.177, 0.224) | < 0.001 | 0.254 (0.231, 0.276) | < 0.001 |
| DALYs | High systolic blood pressure | Pooled, unadjusted | 6496 | -0.205 (-0.228, -0.181) | < 0.001 | -0.199 (-0.222, -0.176) | < 0.001 |
| **C. Association of change across locations (EAPC, 1990–2021)** | | | | | | | |
| Incidence | Smoking | Change across locations | 21 | 0.127 (-0.322, 0.530) | 0.582 | — | — |
| Incidence | High systolic blood pressure | Change across locations | 21 | -0.083 (-0.497, 0.361) | 0.720 | — | — |
| Prevalence | Smoking | Change across locations | 21 | 0.068 (-0.375, 0.485) | 0.771 | — | — |
| Prevalence | High systolic blood pressure | Change across locations | 21 | -0.493 (-0.763, -0.079) | 0.023 | — | — |
| Deaths | Smoking | Change across locations | 21 | 0.027 (-0.409, 0.454) | 0.907 | — | — |
| Deaths | High systolic blood pressure | Change across locations | 21 | 0.016 (-0.419, 0.444) | 0.947 | — | — |
| DALYs | Smoking | Change across locations | 21 | -0.017 (-0.445, 0.418) | 0.942 | — | — |
| DALYs | High systolic blood pressure | Change across locations | 21 | 0.114 (-0.334, 0.520) | 0.622 | — | — |

Note. Exposure is expressed as the summary exposure value (SEV) for the 55 years and older age group, from the GBD 2021 risk-factor release (IHME-GBD_2021_DATA); a higher SEV indicates greater population exposure. Ranges are given at 95% confidence intervals (CI) obtained by Fisher z transformation. Spearman rank correlations are computed between the SEV and the age-standardized SAH rate, both for women aged 55 years and older; rates were standardized to the WHO World Standard Population renormalized within the 55 years and older age range. Unadjusted correlations are pooled across all location-years. Adjusted correlations control for the Sociodemographic Index (SDI) and year by rank partial correlation. Panel C correlates the estimated annual percentage change (EAPC) of the SEV with the EAPC of the SAH rate across the 21 GBD regions; the global level is not included in this panel because it is itself an aggregate of those regions, and the adjusted columns do not apply to it. In panel C the direction of the EAPC was concordant in 19 of 21 regions for smoking and in 11 of 21 regions for high systolic blood pressure. Abbreviations: CI, confidence interval; DALYs, disability-adjusted life-years; EAPC, estimated annual percentage change; SAH, subarachnoid hemorrhage; SDI, Sociodemographic Index; SEV, summary exposure value.
